# Association between vitamin intake and biological aging: evidence from NHANES 2007–2018

**DOI:** 10.1016/j.jnha.2026.100776

**Published:** 2026-01-14

**Authors:** Xinyu Zhang, Yujie Xu, Xiaoyu Wang, Mengxue Chen, Jingyuan Xiong, Guo Cheng

**Affiliations:** a West China School of Nursing, Sichuan University, Chengdu 610041, Sichuan, China; b Laboratory of Molecular Translational Medicine, Center for Translational Medicine, Key Laboratory of Birth Defects and Related Diseases of Women and Children, Ministry of Education, Maternal & Child Nutrition Center, West China Second University Hospital, Sichuan University, Chengdu 610041, Sichuan, China; c Department of Occupational and Environmental Health, Healthy Food Evaluation Research Center, West China School of Public Health and West China Fourth Hospital, Sichuan University, Chengdu 610041, Sichuan, China; d Health Promotion and Food Nutrition and Safety Key Laboratory of Sichuan Province, Chengdu, Sichuan, China; e Children's Medicine Key Laboratory of Sichuan Province, Chengdu 610041, Sichuan, China

**Keywords:** Vitamins, Biological aging, Mixture analysis, NHANES, Quantile g-computation

## Abstract

**Background:**

The combined effect of vitamin mixture on biological aging, along with the specific contribution of individual components, remains unclear. This study investigated the associations between a mixture of 11 dietary vitamins and biological aging.

**Methods:**

This cross-sectional study included 15050 adults from NHANES 2007–2018. Daily intakes of 11 vitamins were estimated using the multiple source method to account for within-person variation from two 24 -h recalls, incorporating both food and supplement contributions. Total vitamin intake was calculated as their sum. Biological aging was assessed using three established indicators: KDM-acceleration and PhenoAge-acceleration (derived as regression residuals of biological age on chronological age), and homeostatic dysregulation (HD, a composite physiological score). Multiple linear regression, restricted cubic spline regression, and quantile g-computation were used to assess individual and joint associations.

**Results:**

The median age was 51.0 years, and 51.5% were female. Higher total vitamin intake was significantly associated with reduced biological aging (KDM-acceleration: β = −1.281; PhenoAge-acceleration: β = −1.379; HD: β = −0.046). Dose-response relationships were linear (all *P*_nonlinear_ > 0.05). Stratified analyses revealed stronger associations in males and individuals with comorbidity. Vitamin C was the primary protective component, followed by vitamin B2.

**Conclusions:**

Higher intake of dietary vitamin mixture was associated with slower biological aging, with vitamin C as the key protective driver. These findings support recommending vitamin-rich diets to promote healthy aging.

## 1 Introduction

As global population aging accelerates, promoting healthy aging has become a public health priority worldwide [1]. Consequently, the focus of modern medicine is shifting from merely extending lifespan to prolonging health span by delaying biological aging [2]. Aging is a complex process characterized by degenerative changes in the structure and function of the body. It is influenced by genetic, environmental, and lifestyle factors, leading to considerable heterogeneity in aging trajectories among individuals [3]. In this context, biological age, which integrates various physiological parameters such as blood biomarkers, offers a more accurate assessment of an individual's functional status and rate of aging than chronological age alone [4]. Common established measures of biological ages include the Klemera–Doubal method biological age (KDM-BA) [5], PhenoAge [6], and homeostatic dysregulation (HD) [7].

There is growing scientific interest in identifying modifiable factors, particularly nutritional factors, that can slow the rate of biological aging [4]. Numerous cellular and animal studies suggest that various vitamins may attenuate the aging process through mechanisms such as antioxidant activity, anti-inflammatory effects, participation in energy metabolism, and epigenetic regulation [[8], [9], [10]]. However, current epidemiological evidence has several key limitations. Firstly, most studies have examined vitamins in isolation [11,12], neglecting the reality that they are consumed as a mixture where interactive effects may occur. Secondly, those investigating multivitamin mixtures have not quantified the relative contribution of individual components and not identified the key protective drivers [13,14]. Thirdly, the majority of prior research has focused on specific aging-related outcomes like telomere length, cognitive function, immune response, physical performance, or disease incidence [10,11,15], rather than utilizing integrated biomarkers like KDM-BA, PhenoAge, or HD that capture the multisystemic nature of aging. Thus, the overall effect of vitamins on biological aging remains incompletely understood.

To address these knowledge gaps, we utilized data from the National Health and Nutrition Examination Survey (NHANES) 2007–2018 to comprehensively investigate the relationship of vitamins with biological aging using three established biomarkers: KDM-acceleration, PhenoAge-acceleration, and HD. We aimed to evaluate both the individual contributions and the joint effect of the vitamin mixture on three established biomarkers of accelerated biological aging.

## 2 Methods

### 2.1 Data source and study population

This study included 15050 participants from NHANES 2007–2018, who met the following eligible criteria: non-pregnant adults (≥18 years); plausible daily energy intake (male: 500−8000 kcal/day; female: 500−5000 kcal/day) [16]; possessed complete data for biological aging biomarkers, dietary intake, and relevant covariates. NHANES has been approved by the Ethics Review Board of the National Center for Health Statistics. A participants’ selection flowchart is in Figure S1.

### 2.2 Assessment of vitamin intakes

NHANES collected dietary data using two 24 -h dietary recalls. The first interview was conducted in-person at the Mobile Examination Center (MEC), and the second was performed via telephone 3–10 days later [17]. In our study, we examined vitamins A, B1, B2, B3, B6, B9, B12, C, D, E, and K. These comprised all vitamin types for which data were captured in NHANES. For each participant, daily intake of each vitamin was calculated by summing daily dietary intake and supplement intake and then averaging the intakes across the two recall days. Total vitamin intake was calculated as the sum of daily intakes of these 11 vitamins [4,16,18].

### 2.3 Construction of biological aging markers

We estimated biological age using three established algorithms: KDM-BA [5], Levine-method PhenoAge [6], and HD [7]. KDM-BA is derived by regressing multiple biomarkers on chronological age, while PhenoAge incorporates mortality risk factors. HD quantifies the Mahalanobis distance of an individual's biomarker profile from a healthy reference population (aged 20–30). Calculations were performed using the "BioAge" R package, with algorithms trained on NHANES III (1988–1994) biomarker data as per Nakazato et al. [19]. We selected 12 blood biomarkers for these calculations: albumin, alkaline phosphatase, C-reactive protein, total cholesterol, glycated hemoglobin, creatinine, uric acid, blood urea nitrogen, white blood cell count, lymphocyte percent, mean cell volume, and red cell distribution width (Table S1) [20]. Blood samples were collected in MEC on the same day as the in-person dietary recall.

To represent accelerated biological aging, we calculated KDM-acceleration and PhenoAge-acceleration using the residual method to fully account for chronological age effects. Positive values indicate accelerated aging relative to chronological age peers. For HD, which directly quantifies the pace of biological aging, we used the untransformed HD values, with higher values indicating faster aging.

### 2.4 Assessment of covariates

Covariates included age (continuous, years), sex (male, female), race (non-Hispanic White, non-Hispanic Black, Mexican American, other race), educational level (<high school, high school, >high school), marital status (coupled, single/separated), poverty-income ratio (PIR; <1.0, 1.0−3.0, ≥3.0), body mass index (BMI; <25 kg/m^2^, 25−30 kg/m^2^, ≥30 kg/m^2^), smoking status (never smoked, former smoker, current smoker), alcohol consumption (yes, no), physical activity level (inactive, moderate, active, highly active), daily energy intake (continuous, kcal/day), use of any supplements (yes, no), comorbidity (yes, no; including hypertension, diabetes, cardiovascular diseases, and cancer).

### 2.5 Statistical analysis

Habitual intake was estimated using the multiple source method (MSM), which applies linear mixed-effects models to two 24 -h recalls to account for within-person variation. The validity of the estimates was supported by satisfactory shrinkage factors (range: 0.321−0.670, mean: 0.507) and high Spearman correlations with arithmetic mean of the two recalls (most vitamins: r = 0.956−0.991). Vitamin B1 required special handling (median of two recalls) due to minimal between-person variation. Details are in Table S2.

We incorporated the NHANES-recommended complex survey design variables for analyses: WTDR2D, SDMVPSU, and SDMVSTRA. All habitual vitamin intakes underwent natural log (ln) transformation to approximate a normal distribution [18]. In the descriptive analysis of baseline characteristics, continuous variables with a normal distribution were presented as mean ± standard deviation, whereas those with a non-normal distribution were expressed as median (interquartile range). Categorical variables were presented as number (percentage). Group differences were assessed using analysis of variance for continuous variables and the chi-square test for categorical variables.

We employed weighted multiple linear regression to examine the associations between habitual vitamin intake (categorized into quartiles, Q1-Q4) and the three biological aging indicators [21]. Results were presented as β coefficients with 95% confidence intervals (95% CI), reflecting the extent of accelerated biological aging. Four adjusted models were constructed: Model 1 adjusted for age, sex, and race; Model 2 further adjusted for educational level, marital status, and PIR; Model 3 added adjustments for BMI, smoking status, alcohol consumption, physical activity level, daily energy intake, and supplement use; Model 4 additionally accounted for comorbidity. A trend test was also conducted within Model 4 by treating vitamin quartiles as a continuous variable.

Subsequently, restricted cubic spline (RCS) regression was conducted to assess the potential nonlinear relationship between vitamin intake and biological aging indicators. Four knots were positioned at the 5th, 35th, 65th, and 95th percentiles of ln-transformed vitamin intake, with the median intake level used as the reference [18,20].

Stratified analyses were performed to evaluate potential effect modification by the following factors: age (<60 years, ≥60 years), sex (male, female), race (non-Hispanic White, non-Hispanic Black, Mexican American, other race), educational level (<high school, high school, >high school), marital status (coupled, single/separated), PIR (<1.0, 1.0−3.0, ≥3.0), BMI (<25 kg/m^2^, 25−30 kg/m^2^, ≥30 kg/m^2^), smoking status (never smoked, former smoker, current smoker), alcohol consumption (yes, no), physical activity level (inactive, moderate, active, highly active), daily energy intake (<1500 kcal/day, 1500−2500 kcal/day, ≥2500 kcal/day), use of any supplements (yes, no), comorbidity (yes, no).

Furthermore, quantile g-computation (QGC) was used to evaluate the joint effect of all 11 vitamins, estimating the change in biological aging indicators when all vitamins increased simultaneously by one quantile, and decomposing the contribution of each vitamin [22].

Finally, we performed sensitivity analyses using vitamins from dietary sources only to assess the robustness of our primary findings. Multiple linear regression, RCS, and QGC were repeated with dietary-only intake variables.

All analyses were conducted using R software (version 4.4.1). with a two-sided *P* value < 0.05 considered statistically significant.

## 3 Results

### 3.1 Basic characteristics

The median age of 15050 participants was 51.0 years, and 51.5% were female. The median KDM-BA was 48.5 years, PhenoAge was 49.1 years, and the HD was 0.94. Participants in higher quartiles (Q4) of total vitamin intake were generally older, had higher socioeconomic status, healthier lifestyles, and exhibited less accelerated biological aging across all three indicators compared to those in Q1 (Table 1).

**Table 1:** Basic characteristics according to the quartiles of ln-transformed total vitamin intake.

	Total vitamin intake (N = 15050)					
Variables	All N = 15050	Q1: ≤4.33 N = 3763	Q2: 4.33−4.69 N = 3762	Q3: 4.69−5.07 N = 3762	Q4: >5.07 N = 3763	*P*
Age (years)	51.0 (36.0, 65.0)	46.0 (33.0, 61.0)	47.0 (33.0, 61.0)	50.0 (35.0, 64.0)	59.0 (45.0, 71.0)	<0.001
Sex, n (%)						<0.001
Male	7292 (48.5%)	1679 (44.6%)	1840 (48.9%)	1881 (50.0%)	1892 (50.3%)	
Female	7758 (51.5%)	2084 (55.4%)	1922 (51.1%)	1881 (50.0%)	1871 (49.7%)	
Race, n (%)						<0.001
Non-Hispanic White	6860 (45.6%)	1611 (42.8%)	1549 (41.2%)	1655 (44.0%)	2045 (54.3%)	
Non-Hispanic Black	2899 (19.3%)	850 (22.6%)	752 (20.0%)	719 (19.1%)	578 (15.4%)	
Mexican American	2377 (15.8%)	614 (16.3%)	693 (18.4%)	618 (16.4%)	452 (12.0%)	
Other Race	2914 (19.4%)	688 (18.3%)	768 (20.4%)	770 (20.5%)	688 (18.3%)	
Educational level, n (%)						<0.001
<High school	1436 (9.54%)	477 (12.7%)	399 (10.6%)	306 (8.13%)	254 (6.75%)	
High school	5558 (36.9%)	1726 (45.9%)	405 (37.3%)	1306 (34.7%)	1121 (29.8%)	
>High school	8056 (53.5%)	1560 (41.5%)	1958 (52.0%)	2150 (57.2%)	2388 (63.5%)	
Marital status, n (%)						<0.001
Coupled	9200 (61.1%)	2115 (56.2%)	2343 (62.3%)	2338 (62.1%)	2404 (63.9%)	
Single/Separated	5850 (38.9%)	1648 (43.8%)	1419 (37.7%)	1424 (37.9%)	1359 (36.1%)	
PIR, n (%)						<0.001
<1.0	2953 (19.6%)	1062 (28.2%)	765 (20.3%)	663 (17.6%)	463 (12.3%)	
1.0−3.0	6558 (43.6%)	1764 (46.9%)	1644 (43.7%)	1624 (43.2%)	1526 (40.6%)	
≥3.0	5539 (36.8%)	937 (24.9%)	1353 (36.0%)	1475 (39.2%)	1774 (47.1%)	
BMI, n (%)						<0.001
<25 kg/m^2^	3994 (26.5%)	949 (25.2%)	947 (25.2%)	1061 (28.2%)	1037 (27.6%)	
25−30 kg/m^2^	6035 (40.1%)	1645 (43.7%)	1627 (43.2%)	1384 (36.8%)	1379 (36.6%)	
≥30 kg/m^2^	5021 (33.4%)	1169 (31.1%)	1188 (31.6%)	1317 (35.0%)	1347 (35.8%)	
Smoking status, n (%)						<0.001
Never	8237 (54.7%)	1808 (48.0%)	2044 (54.3%)	2219 (59.0%)	2166 (57.6%)	
Former	3863 (25.7%)	758 (20.1%)	969 (25.8%)	973 (25.9%)	1163 (30.9%)	
Current	2950 (19.6%)	1197 (31.8%)	749 (19.9%)	570 (15.2%)	434 (11.5%)	
Alcohol consumption, n (%)						<0.001
No	10632 (70.6%)	2832 (75.3%)	2610 (69.4%)	2572 (68.4%)	2618 (69.6%)	
Yes	4418 (29.4%)	931 (24.7%)	1152 (30.6%)	1190 (31.6%)	1145 (30.4%)	
Physical activity level, n (%)						0.003
Inactive	8396 (55.8%)	2127 (56.5%)	2103 (55.9%)	2053 (54.6%)	2113 (56.2%)	
Moderate	3476 (23.1%)	809 (21.5%)	840 (22.3%)	912 (24.2%)	915 (24.3%)	
Active	578 (3.84%)	173 (4.60%)	146 (3.88%)	143 (3.80%)	116 (3.08%)	
Highly active	2600 (17.3%)	654 (17.4%)	673 (17.9%)	654 (17.4%)	619 (16.4%)	
Daily energy intake (kcal/day)	1902 (1452, 2449)	1597 (1214, 2055)	1952 (1524,2482)	2029 (1561, 2622)	2046 (1580, 2624)	<0.001
Supplement use, n (%)						0.000
Yes	7823 (52.0%)	970 (25.8%)	1418 (37.7%)	2152 (57.2%)	3283 (87.2%)	
No	7227 (48.0%)	2793 (74.2%)	2344 (62.3%)	1610 (42.8%)	480 (12.8%)	
Comorbidity, n (%)						<0.001
No	7772 (51.6%)	2012 (53.5%)	2070 (55.0%)	2054 (54.6%)	1636 (43.5%)	
Yes	7278 (48.4%)	1751 (46.5%)	1692 (45.0%)	1708 (45.4%)	2127 (56.5%)	
KDM-BA	48.5 (33.9, 62.3)	45.1 (31.2, 60.2)	45.1 (31.8, 59.5)	47.2 (33.2, 61.1)	55.0 (41.0, 66.2)	<0.001
KDM-acceleration	−1.35 (−6.47, 4.31)	−0.35 (−5.77, 5.12)	−1.13 (−5.85, 4.67)	−1.72 (−6.80, 3.70)	−2.40 (−7.48, 3.65)	<0.001
PhenoAge	49.1 (33.0, 64.8)	46.0 (31.3, 62.3)	45.7 (30.9, 61.5)	47.4 (32.0, 63.7)	56.8 (41.7, 70.0)	<0.001
PhenoAge-acceleration	−0.80 (−4.18, 3.14)	0.49 (−2.89, 4.55)	−0.54 (−3.81, 3.52)	−1.33 (−4.60, 2.24)	−1.88 (−5.22, 1.92)	<0.001
HD	0.94 (0.76, 1.23)	0.95 (0.77, 1.28)	0.91 (0.74, 1.20)	0.91 (0.74, 1.18)	0.97 (0.79, 1.26)	<0.001

Abbreviations: BMI, body mass index; PIR, poverty-income ratio; KDM-BA, Klemera and Doubal Model biological age; KDM-acceleration, residual-based acceleration of KDM-BA relative to chronological age; PhenoAge-acceleration, residual-based acceleration of PhenoAge relative to chronological age; HD, homeostatic dysregulation.

### 3.2 Associations between vitamin intake and biological aging

Higher total vitamin intake was consistently associated with reduced biological aging. In the fully adjusted model (Model 4), participants in Q4 showed significantly lower KDM-acceleration (β = −1.281, *P* = 0.001) and PhenoAge-acceleration (β = −1.379, *P* < 0.001) compared to Q1, while HD showed a non-significant trend toward reduction (β = −0.046, *P* = 0.099) (Table 2 and Figure S2). For individual vitamins in Model 4, KDM-acceleration was significantly lower in Q4 of vitamins B2, B9, and C; PhenoAge-acceleration was significantly lower in Q4 for all vitamins; no significant reductions in HD were observed in Q4 for any vitamin (Tables S3-S13).

**Table 2:** Associations between ln-transformed total vitamin intake and biological aging indicators.

		Q1	Q2		Q3		Q4		
Biological aging	Model	Ref	β (95% CI)	*P*	β (95% CI)	*P*	β (95% CI)	*P*	*P* for trend
KDM-acceleration	Model1	Ref	−1.118 (−1.751, −0.486)	<0.001	−1.709 (−2.361, −1.057)	<0.001	−1.890 (−2.501, −1.279)	<0.001	
	Model2	Ref	−0.926 (−1.532, −0.320)	0.003	−1.479 (−2.122, −0.837)	<0.001	−1.613 (−2.199, −1.027)	<0.001	
	Model3	Ref	−0.849 (−1.452, −0.247)	0.007	−1.216 (−1.922, −0.510)	0.001	−1.349 (−2.116, −0.582)	0.001	
	Model4	Ref	−0.751 (−1.338, −0.165)	0.013	−1.117 (−1.815, −0.419)	0.002	−1.281 (−2.033, −0.530)	0.001	0.002
PhenoAge-acceleration	Model1	Ref	−1.154 (−1.603, −0.705)	<0.001	−2.026 (−2.496, −1.555)	<0.001	−2.554 (−3.035, −2.074)	<0.001	
	Model2	Ref	−0.867 (−1.266, −0.468)	<0.001	−1.676 (−2.117, −1.235)	<0.001	−2.116 (−2.545, −1.688)	<0.001	
	Model3	Ref	−0.574 (−0.942, −0.207)	0.003	−1.069 (−1.521, −0.617)	<0.001	−1.424 (−1.886, −0.963)	<0.001	
	Model4	Ref	−0.508 (−0.872, −0.144)	0.007	−1.002 (−1.454, −0.550)	<0.001	−1.379 (−1.842, −0.916)	<0.001	<0.001
HD	Model1	Ref	−0.092 (−0.140, −0.043)	<0.001	−0.095 (−0.146, −0.043)	<0.001	−0.101 (−0.144, −0.058)	<0.001	
	Model2	Ref	−0.072 (−0.118, −0.026)	0.003	−0.071 (−0.122, −0.019)	0.009	−0.071 (−0.114, −0.027)	0.002	
	Model3	Ref	−0.057 (−0.100, −0.013)	0.012	−0.046 (−0.102, 0.010)	0.103	−0.051 (−0.107, 0.004)	0.068	
	Model4	Ref	−0.048 (−0.090, −0.006)	0.025	−0.038 (−0.091, 0.016)	0.161	−0.046 (−0.100, 0.009)	0.099	0.187

Abbreviations: KDM-acceleration, residual-based acceleration of Klemera and Doubal Model biological age (KDM-BA) relative to chronological age; PhenoAge-acceleration, residual-based acceleration of PhenoAge relative to chronological age; HD, homeostatic dysregulation.

• Model 1: adjustment for age, sex, and race.

• Model 2: Model 1 + adjustment for educational level, marital status, and poverty income ratio.

• Model 3: Model 2 + adjustment for body mass index, smoking status, alcohol consumption, physical activity level, daily energy intake, and supplement use.

• Model 4: Model 3 + adjustment for comorbidity (including hypertension,diabetes, cardiovascular diseases, and cancer).

Note: The trend test is based on the Model 4 and uses quartiles as continuous variables for analysis.

### 3.3 Non-linear association assessment

RCS analyses revealed predominantly linear relationships between vitamin intake and aging indicators. Total vitamin intake showed significant linear inverse associations with all three aging indicators (*P*_nonlinear_ = 0.135 for KDM-acceleration, *P*_nonlinear_ = 0.058 for PhenoAge-acceleration, *P*_nonlinear_ = 0.280 for HD, Figure S3). The analysis of individual vitamins revealed widespread linear associations. While all 11 vitamins exhibited significant linear relationships with PhenoAge-acceleration, significant linear associations with KDM-acceleration were observed for vitamins B9, B12, and C, and with HD for vitamins B3, C, and E (Figures S4-S14).

### 3.4 Stratified analyses

Stratification by demographic and health characteristics revealed significant interaction effects for sex and daily energy intake (*P*_interaction_<0.05), with several additional factors showing borderline significant interactions (0.05<*P*_interaction_<0.10). The protective effects of total vitamin intake varied across subgroups: For KDM-acceleration, stronger inverse associations were observed in males, individuals with BMI 25−30 kg/m^2^, and current alcohol drinkers. For PhenoAge-acceleration, more pronounced effects were found in those with lower education levels and individuals with BMI < 25 kg/m^2^. For HD, stronger protective associations were evident in participants with daily energy intake <1500 kcal/day and those with comorbidity (Table S14 and Figure S15).

### 3.5 Joint effects of vitamin mixture

**Fig. 1:**
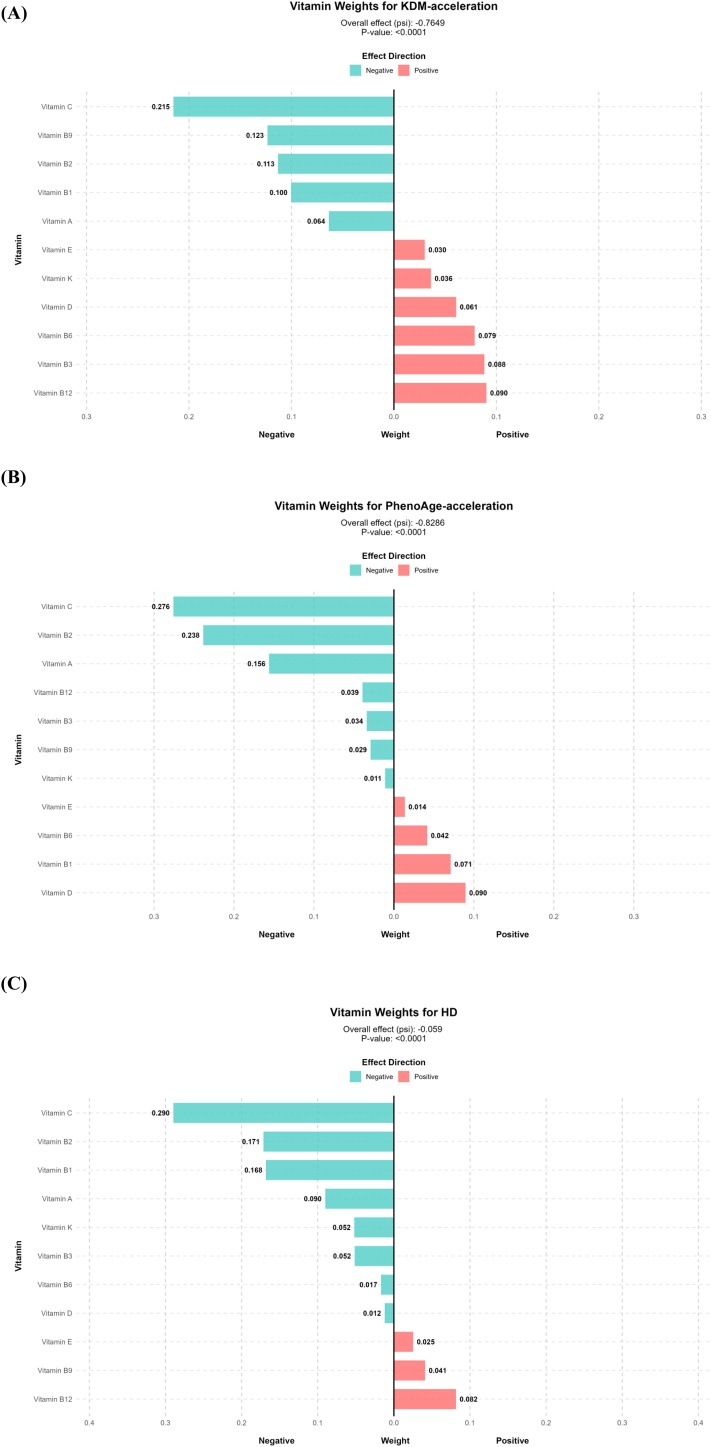
Weights and individual effects of each vitamin on biological aging indicators by quantile g-computation model. Biological aging indicators included KDM-acceleration (A), PhenoAge-acceleration (B), HD (C). Abbreviations: KDM-acceleration, residual-based acceleration of Klemera and Doubal Model biological age (KDM-BA) relative to chronological age; PhenoAge-acceleration, residual-based acceleration of PhenoAge relative to chronological age; HD, homeostatic dysregulation.

QGC analysis demonstrated significant joint protective effects of the 11-vitamin mixture against all aging indicators (all *P* < 0.05). Each one-quantile increase in the vitamin mixture was associated with a reduction of 0.76 years in KDM-acceleration, 0.83 years in PhenoAge-acceleration, and 0.06 units in HD (Table S15). Vitamin C was identified as the primary protective driver, with the highest positive weights across all aging indicators (KDM-acceleration: 0.215; PhenoAge-acceleration: 0.276; HD: 0.290), followed by vitamin B2. Conversely, vitamins B12 and D exhibited negative weights, indicating potential antagonistic effects (Fig. 1 and Tables S16-S18).

### 3.6 Sensitivity analyses

Results from sensitivity analyses using dietary-only vitamins were consistent with the primary findings. (1) In multiple linear regression, total dietary vitamin intake remained inversely associated with all three biological aging indicators (all *P* < 0.05, Table S19). (2) RCS analyses indicated approximately linear inverse dose-response relationships between dietary vitamin intake and both KDM-acceleration and HD, and a nonlinear inverse relationship with PhenoAge-acceleration (Figure S16). (3) QGC showed that vitamin C contributed the largest weight to the joint protective effect against both KDM-acceleration and PhenoAge-acceleration, while vitamin B6 was the top contributor for HD, followed by vitamin C (Figure S17).

## 4 Discussion

This study investigated the associations between a mixture of 11 vitamins and three biological aging indicators in a nationally representative sample from NHANES 2007–2018. We found that higher total vitamin intake was consistently associated with decelerated biological aging. This protective association was observed for most individual vitamins and was robust in sensitivity analyses restricted to dietary sources. Notably, vitamin C was identified as the primary contributor to the overall protective effect of the vitamin mixture.

The overall protective association we observed is consistent with established evidence on the anti-aging roles of vitamins, with dietary sources representing the primary and recommended means of intake. For instance, higher adherence to vitamin-rich dietary patterns like the Mediterranean diet, plant-based diet, and dietary approaches to stop hypertension diet, has been associated with reduced biological aging and lower mortality [23,24]. Our sensitivity analyses also confirmed that the protective associations remained robust when considering only dietary vitamin sources. These findings underscore the importance of obtaining vitamins through whole foods. In addition, some studies indicated that multivitamin intake may improve lipids levels, enhance memory performance in older adults, reduce the incidence of age-related macular degeneration, and lower cause-specific mortality in cancer patients [13,14,25]. Collectively, these earlier findings provided a coherent scientific backdrop that supported our results. By linking vitamin intake with integrative biomarkers of biological aging, our study helped to consolidate previously fragmented insights into a unified framework, suggesting a systemic anti-aging effect.

Beyond establishing this overall association, our study revealed that the protective effect was more pronounced in specific subgroups, including males, current alcohol drinkers, and those with comorbidity. This suggests that individuals with higher underlying physiological stress or inflammation might derive greater benefit from adequate vitamin intake [26]. Some studies have demonstrated that the health benefits of vitamins are more pronounced among these groups [18,26,27].

A central strength of our analysis was quantifying the relative contribution of each vitamin. The QGC analysis identified vitamin C as the most influential protective component across all three aging indicators. As a potent antioxidant, vitamin C plays a crucial role in protecting against oxidative damage, a key mechanism in aging [8]. Importantly, a recent NHANES-based study observed a nonlinear relationship between serum vitamin C and PhenoAge acceleration, with benefits plateauing beyond a certain concentration [28]. This suggests that while ensuring adequate intake is crucial, the benefits of vitamin C on biological aging may not increase indefinitely with higher doses. This finding strongly reinforces dietary recommendations prompting adequate vitamin C intake. Substantial positive weight was also observed for vitamin B2, consistent with their established roles in supporting metabolic and immune health [29].

An interesting finding was the negative weights observed for vitamins B12 and D, suggesting their associations with biological aging were in the opposite direction to the overall protective effect of the mixture. This observation could be explained by several mechanisms. First, antagonistic interactions within the vitamin mixture, such as competition for shared absorption transporters or metabolic pathways, might attenuate the net protective effect [30]. Second, and perhaps more likely, these negative weights may indicate nonlinear dose-response relationships [31]. This interpretation is supported by our RCS analysis, which suggested that higher intake of vitamin D might be associated with an increase in KDM-acceleration and PhenoAge-acceleration.

The source and level of vitamin intake are crucial for interpreting our findings. Sensitivity analyses using dietary-only vitamins revealed that the protective associations remained significant, and vitamin C persisted as the primary contributor. Importantly, supplementary analyses indicated that higher intake in our study primarily corresponds to achieving nutritionally adequate levels: over 90% of participants in the highest intake quartile met the Recommended Dietary Allowance for most vitamins. This suggests that the observed anti-aging benefits are associated with adequate intake attainable through diet [23]. However, the benefits likely reflect the combined effect of vitamins and correlated dietary components within whole foods, which our data cannot fully disentangle.

This study has several strengths, including being the first to investigate vitamin mixture effects on biological aging, identifying vitamin C's key role, and utilizing a large, nationally representative sample. However, this study also has unavoidable limitations. First, as a cross-sectional survey, it cannot establish causality, and the associations may be subject to residual confounding. Second, although we used the MSM to estimate habitual vitamin intake from two 24 -h recalls, self-reported dietary data remain subject to measurement errors including recall bias. Third, the QGC model robustly estimates joint effects, its assumptions of linearity and additivity may not capture the full complexity of nutrient interactions. Furthermore, independent validation in other cohorts is warranted.

## 5 Conclusions

Our findings from the NHANES cohort demonstrated that higher intake of a dietary vitamin mixture was significantly associated with decelerated biological aging. These results strengthen the evidence for dietary recommendations promoting vitamin-rich diets for healthy aging. Notably, vitamin C showed the strongest protective effect against systematic biological aging. These observations highlight the potential of targeted nutritional interventions in modulating the aging process. Future prospective and mechanistic studies are needed to establish causality and unravel the underlying biological pathways.

## CRediT authorship contribution statement

XYZ, JYX, and GC designed research; XYZ, YJX, XYW, and MXC analyzed data; XYZ wrote paper; GC had primary responsibility for final content. All authors read and approved the final manuscript.

## Consent for publication

Not applicable.

## Ethics approval and consent to participate

The study was conducted according to the guidelines of the Declaration of Helsinki and received ethical clearance from the Institutional Review Board of National Center for Health Statistics, and all subjects agreed to participate after signing the required documentation.

## Funding

This study was supported by National Natural Science Foundation of China (Grant number: 82173512). The funder had no role in the design and conduct of the study.

## Availability of data and materials

All data generated or analyzed during this study are included in this published article and its supplementary information files.

## Declaration of competing interest

The authors declare no competing interests.

## References

[1] Christensen K., Doblhammer G., Rau R., Vaupel J.W. (2009). Ageing populations: the challenges ahead. Lancet..

[2] Tessier A.-J., Wang F., Korat A.A., Eliassen A.H., Chavarro J., Grodstein F. (2025). Optimal dietary patterns for healthy aging. Nat Med..

[3] López-Otín C., Blasco M.A., Partridge L., Serrano M., Kroemer G. (2013). The Hallmarks of Aging. Cell..

[4] Xing W., Gao W., Zhao Z., Xu X., Bu H., Su H. (2023). Dietary flavonoids intake contributes to delay biological aging process: analysis from NHANES dataset. J Transl Med..

[5] Klemera P., Doubal S. (2006). A new approach to the concept and computation of biological age. Mech Ageing Dev..

[6] Levine M.E., Lu A.T., Quach A., Chen B.H., Assimes T.L., Bandinelli S. (2018). An epigenetic biomarker of aging for lifespan and healthspan. Aging.

[7] Cohen A.A., Milot E., Yong J., Seplaki C.L., Fülöp T., Bandeen-Roche K. (2013). A novel statistical approach shows evidence for multi-system physiological dysregulation during aging. Mech Ageing Dev..

[8] Monacelli F., Acquarone E., Giannotti C., Borghi R., Nencioni A. (2017). Vitamin C, aging and Alzheimer’s disease. Nutrients..

[9] Fraczek P.M., Duran P., Yang B.A., Ferre V., Alawieh L., Castor-Macias J.A. (2025). Vitamin A retinoic acid contributes to muscle stem cell and mitochondrial function loss in old age. JCI Insight..

[10] Kaźmierczak-Barańska J., Karwowski B.T. (2024). The protective role of vitamin K in aging and age-related diseases. Nutrients..

[11] Cai Y., Zhong Y., Zhang H., Lu P., Liang Y., Hu B. (2023). Association between dietary vitamin C and telomere length: a cross-sectional study. Front Nutr..

[12] Molavi Vasei F., Zamanian M.Y., Golmohammadi M., Mahmoodi M., Khademalhosseini M., Tavakoli T. (2024). The impact of Vitamin E supplementation on oxidative stress, cognitive functions, and aging‐related gene expression in aged mice. Food Sci Nutr..

[13] Harris E., Macpherson H., Pipingas A. (2015). Improved blood biomarkers but no cognitive effects from 16 weeks of multivitamin supplementation in healthy older adults. Nutrients..

[14] Yeung L.-K., Alschuler D.M., Wall M., Luttmann-Gibson H., Copeland T., Hale C. (2023). Multivitamin supplementation improves memory in older adults: a randomized clinical trial. Am J Clin Nutr..

[15] Shea M.K., Barger K., Dawson‐Hughes B., Leurgans S.E., Fu X., James B.D. (2023). Brain Vitamin D forms, cognitive decline, and neuropathology in community‐dwelling older adults. Alzheimers Dement..

[16] Liang F., Lu M., Zhou Y. (2024). Associations between single and multiple dietary vitamins and the risk of periodontitis: results from NHANES 2009–2014. Front Nutr..

[17] Ahluwalia N., Dwyer J., Terry A., Moshfegh A., Johnson C. (2016). Update on NHANES dietary data: focus on collection, release, analytical considerations, and uses to inform public policy. Adv Nutr..

[18] Qi X., Wang X., Cheng L., Li Y., Dang K., Yang S. (2025). Dietary carotenoid intakes and biological aging among US adults, NHANES 1999–2018. Nutr J..

[19] Kwon D., Belsky D.W. (2021). A toolkit for quantification of biological age from blood chemistry and organ function test data: BioAge. GeroScience..

[20] An S., Qin J., Gong X., Li S., Ding H., Zhao X. (2024). The mediating role of body mass index in the association between dietary index for gut microbiota and biological age: a study based on NHANES 2007–2018. Nutrients..

[21] Barr D.B., Wilder L.C., Caudill S.P., Gonzalez A.J., Needham L.L., Pirkle J.L. (2005). Urinary creatinine concentrations in the U.S. population: implications for urinary biologic monitoring measurements. Environ Health Perspect..

[22] Keil A.P., Buckley J.P., O’Brien K.M., Ferguson K.K., Zhao S., White A.J. (2020). A quantile-based g-computation approach to addressing the effects of exposure mixtures. Environ Health Perspect..

[23] Canudas S., Becerra-Tomás N., Hernández-Alonso P., Galié S., Leung C., Crous-Bou M. (2020). Mediterranean diet and telomere length: a systematic review and meta-analysis. Adv Nutr..

[24] Hu F.B. (2024). Diet strategies for promoting healthy aging and longevity: an epidemiological perspective. J Intern Med..

[25] Seddon J.M. (2007). Multivitamin-multimineral supplements and eye disease: age-related macular degeneration and cataract. Am J Clin Nutr..

[26] Wang L., Wang X., Su H., Xu J. (2024). Association between Vitamin A intake and depression among patients with heart failure. ESC Heart Fail..

[27] Kim S.-M., Lim S.-M., Yoo J.-A., Woo M.-J., Cho K.-H. (2015). Consumption of high-dose vitamin C (1250 mg per day) enhances functional and structural properties of serum lipoprotein to improve anti-oxidant, anti-atherosclerotic, and anti-aging effects via regulation of anti-inflammatory microRNA. Food Funct.

[28] Zhang Y., Gong F., Zhang A.-H., Wang G.-W., Liu Y., Li T. (2025). Serum Vitamin C concentrations are inversely related to biological aging: a population-based cross-sectional study. Eur J Med Res..

[29] Peterson C.T., Rodionov D.A., Osterman A.L., Peterson S.N. (2020). B Vitamins and their role in immune regulation and cancer. Nutrients..

[30] McNulty H., Ward M., Hoey L., Hughes C.F., Pentieva K. (2019). Addressing optimal folate and related B-Vitamin status through the lifecycle: health impacts and challenges. Proc Nutr Soc..

[31] Yong Y., Dong H., Zhou Z., Zhu Y., Gu M., Li W. (2024). Serum 25-hydroxyvitamin D concentrations and their impact on all-cause mortality in Parkinson’s disease: insights from National Health and Nutrition Examination Survey 1999–2020 data. Front Nutr..

