# Supplementary material for: Association between vitamin intake and biological aging: evidence from NHANES 2007–2018
Source: J Nutr Health Aging. 2026 Jan 14;30(2):100776. doi: 10.1016/j.jnha.2026.100776 (PMC12835586; doi:10.1016/j.jnha.2026.100776)
Supplement: Supplementary file 1 [file mmc1.docx]

**Article title:** Association between vitamin intake and biological aging: evidence from NHANES 2007-2018

**Journal name:** Journal of nutrition, health, and aging

**Author names:** Xinyu Zhang, Yujie Xu, Xiaoyu Wang, Mengxue Chen, Jingyuan Xiong^*^, and Guo Cheng^*^

**Corresponding Authors:**

Guo Cheng, PhD, No.17, Section 3 Renmin Nan Road, Chengdu 610041, Sichuan, China. Email: gcheng@scu.edu.cn.

Jingyuan Xiong, PhD, No.17, Section 3 Renmin Nan Road, Chengdu 610041, Sichuan, China. Email: jzx0004@tigermail.auburn.edu.

* Contributed equally as co-corresponding authors.

**Supplementary Materials**

Table S1. Basic characteristics of 12 blood biomarkers according to the quartiles of ln-transformed total vitamin intake 3

Table S2. Multiple Source Method -derived habitual vitamin intake estimates and validation metrics 4

Table S3. Associations between ln-transformed vitamin A intake and biological aging indicators 5

Table S4. Associations between ln-transformed vitamin B1 intake and biological aging indicators 6

Table S5. Associations between ln-transformed vitamin B2 intake and biological aging indicators 7

Table S6. Associations between ln-transformed vitamin B3 intake and biological aging indicators 8

Table S7. Associations between ln-transformed vitamin B6 intake and biological aging indicators 9

Table S8. Associations between ln-transformed vitamin B9 intake and biological aging indicators 10

Table S9. Associations between ln-transformed vitamin B12 intake and biological aging indicators 11

Table S10. Associations between ln-transformed vitamin C intake and biological aging indicators 12

Table S11. Associations between ln-transformed vitamin D intake and biological aging indicators 13

Table S12. Associations between ln-transformed vitamin E intake and biological aging indicators 14

Table S13. Associations between ln-transformed vitamin K intake and biological aging indicators 15

Table S14. Stratified analysis of associations between ln-transformed total vitamin and biological aging indicators 16

Table S15. Joint effects of 11 vitamins on biological aging indicators by quantile g-computation model 19

Table S16. Weights and individual effects of each vitamin on KDM-acceleration by quantile g-computation model 19

Table S17. Weights and individual effects of each vitamin on PhenoAge-acceleration by quantile g-computation model 19

Table S18. Weights and individual effects of each vitamin on HD by quantile g-computation model 20

Table S19. Associations between dietary-only vitamin intake and biological aging indicators (sensitivity analysis) 21

Figure S1. Flowchart of the participants’ selection from NHANES 22

Figure S2. Forest plot of associations between ln-transformed total vitamin intake and biological aging indicators 23

Figure S3. Associations between ln-transformed total vitamin intake and biological aging indicators by restricted cubic spline 24

Figure S4. Associations between ln-transformed vitamin A intake and biological aging indicators by restricted cubic spline 26

Figure S5. Associations between ln-transformed vitamin B1 intake and biological aging indicators by restricted cubic spline 28

Figure S6. Associations between ln-transformed vitamin B2 intake and biological aging indicators by restricted cubic spline 30

Figure S7. Associations between ln-transformed vitamin B3 intake and biological aging indicators by restricted cubic spline 32

Figure S8. Associations between ln-transformed vitamin B6 intake and biological aging indicators by restricted cubic spline 34

Figure S9. Associations between ln-transformed vitamin B9 intake and biological aging indicators by restricted cubic spline 37

Figure S10. Associations between ln-transformed vitamin B12 intake and biological aging indicators by restricted cubic spline 38

Figure S11. Associations between ln-transformed vitamin C intake and biological aging indicators by restricted cubic spline 40

Figure S12. Associations between ln-transformed vitamin D intake and biological aging indicators by restricted cubic spline 42

Figure S13. Associations between ln-transformed vitamin E intake and biological aging indicators by restricted cubic spline 44

Figure S14. Associations between ln-transformed vitamin K intake and biological aging indicators by restricted cubic spline 46

Figure S15. Forest plot of the stratified analysis of associations between ln-transformed total vitamin and biological aging indicators 48

Figure S16. Associations between dietary-only vitamin intake and biological aging indicators by restricted cubic spline (sensitivity analysis) 49

Figure S17.Weights and individual effects of each dietary-only vitamin on biological aging indicators by quantile g-computation model (sensitivity analysis) 51

**Table S1. Basic characteristics of 12 blood biomarkers according to the quartiles of ln-transformed total vitamin intake**

| **Variables** | **Total vitamin intake (*N=15050*)** | | | | | ***P*** |
| --- | --- | --- | --- | --- | --- | --- |
|  | **All** | **Q1: ≤4.36** | **Q2: 4.36-4.92** | **Q3: 4.92-5.45** | **Q4: >5.45** |  |
|  | ***N=15050*** | ***N=3763*** | ***N=3762*** | ***N=3762*** | ***N=3763*** |  |
| Albumin, g/dL | 4.20 (4.00,4.40) | 4.20 (4.00,4.40) | 4.20 (4.00,4.40) | 4.20 (4.00,4.50) | 4.30 (4.00,4.50) | <0.001 |
| Alkaline phosphatase, U/L | 68.00 (56.00,83.00) | 71.00 (58.00,86.00) | 69.00 (56.00,84.00) | 67.00 (55.00,83.00) | 66.00 (55.00,80.00) | <0.001 |
| Blood urea nitrogen, mg/dL | 13.00 (10.00,17.00) | 12.00 (10.00,16.00) | 13.00 (10.00,16.00) | 13.00 (11.00,17.00) | 14.00 (11.00,18.00) | <0.001 |
| Creatinine, mg/dL | 0.84 (0.72,1.01) | 0.83 (0.71,1.00) | 0.84 (0.71,1.00) | 0.85 (0.72,1.00) | 0.86 (0.72,1.02) | <0.001 |
| C-reactive protein, mg/dL | 0.20 (0.08,0.46) | 0.24 (0.09,0.55) | 0.20 (0.09,0.46) | 0.18 (0.08,0.42) | 0.17 (0.07,0.39) | <0.001 |
| Uric acid, mg/dL | 5.40 (4.40,6.40) | 5.40 (4.40,6.40) | 5.40 (4.50,6.50) | 5.30 (4.40,6.40) | 5.30 (4.40,6.30) | 0.109 |
| Glycated hemoglobin, % | 5.50 (5.30,5.90) | 5.50 (5.30,5.90) | 5.50 (5.30,5.90) | 5.50 (5.23,5.90) | 5.60 (5.30,5.90) | 0.096 |
| Total cholesterol, mg/dL | 190.00 (164.00,218.00) | 189.00 (164.00,218.00) | 190.00 (164.00,218.00) | 190.00 (164.00,218.00) | 190.00 (164.00,219.00) | 0.911 |
| White blood cell count, 1000/uL | 6.90 (5.70,8.40) | 7.20 (5.90,8.60) | 7.05 (5.80,8.40) | 6.90 (5.70,8.30) | 6.80 (5.60,8.10) | <0.001 |
| Lymphocyte percent, % | 30.30 (25.00,36.00) | 30.10 (25.00,35.70) | 30.60 (25.42,36.20) | 30.70 (25.30,36.30) | 29.70 (24.60,35.60) | <0.001 |
| Mean (red) cell volume, fL | 89.30 (86.10,92.40) | 88.90 (85.50,92.20) | 89.00 (85.70,91.90) | 89.40 (86.40,92.60) | 90.00 (86.80,92.90) | <0.001 |
| Red cell distribution width, % | 13.10 (12.50,13.80) | 13.20 (12.60,14.00) | 13.10 (12.50,13.80) | 13.00 (12.50,13.80) | 13.00 (12.50,13.70) | <0.001 |

**Table S2. Multiple source method-derived habitual vitamin intake estimates and validation metrics**

| **Vitamin** | **Shrinkage Factor** | **Correlation with Simple Median*** | **Median of All (mg)** | **All Meet RDA (%)** | **All Above UL (%)** | **Median of Q4 (mg)** | **Q4 Meet RDA (%)** | **Q4 Above UL (%)** | **Diet Contribution (%)** |
| --- | --- | --- | --- | --- | --- | --- | --- | --- | --- |
| VA | 0.473 | 0.973 | 0.484 | 9.5 | 0.3 | 0.644 | 26.9 | 1 | 94.3 |
| VB1 | NA† | 0.740 | 3.686 | 94.8 | 5.2 | 15.593 | 99.4 | 20.6 | 42.4 |
| VB2 | 0.670 | 0.991 | 2.044 | 90.4 | 0 | 3.078 | 97.9 | 0 | 89.1 |
| VB3 | 0.478 | 0.988 | 23.754 | 93.4 | 9.3 | 30.436 | 98.1 | 28.1 | 86.3 |
| VB6 | 0.630 | 0.987 | 2.030 | 75.4 | 0 | 3.556 | 93.8 | 0 | 82.8 |
| VB9 | 0.531 | 0.986 | 0.508 | 72.5 | 5.4 | 0.781 | 91.8 | 17.1 | 86.2 |
| VB12 | 0.601 | 0.978 | 0.005 | 87.1 | 0 | 0.015 | 97.5 | 0 | 74.6 |
| VC | 0.473 | 0.956 | 66.792 | 37.5 | 0 | 134.134 | 90.3 | 0 | 81.6 |
| VD | 0.489 | 0.960 | 0.005 | 9 | 0.1 | 0.010 | 21.9 | 0.1 | 73.4 |
| VE | 0.403 | 0.980 | 6.030 | 0 | 0 | 6.656 | 0.1 | 0 | 100‡ |
| VK | 0.321 | 0.969 | 0.062 | 6.1 | 0 | 0.075 | 13 | 0 | 95.8 |

Abbreviations: RDA, recommended daily allowance; UL, tolerable upper intake level.

Notes:

* Spearman correlation between MSM-estimated habitual intake and simple arithmetic mean of two 24-hour recalls.

† Vitamin B1: median of two recalls used due to singular fit (shrinkage factor = 0).

‡ Vitamin E: no supplement data available in NHANES; estimates represent dietary intake only RDA and UL values based on Institute of Medicine recommendations for adults.

**Table S3. Associations between ln-transformed vitamin A intake and biological aging indicators**

| **Biological aging** | **Model** | **Q1** | | **Q2** | | **Q3** | | **Q4** | | **P for trend** |
| --- | --- | --- | --- | --- | --- | --- | --- | --- | --- | --- |
|  |  | **Ref** |  | **β (95% CI)** | **P value** | **β (95% CI)** | **P value** | **β (95% CI)** | **P value** |  |
| KDM-acceleration | Model1 | Ref |  | -0.275 (-0.743, 0.194) | 0.245 | -1.050 (-1.656, -0.444) | 0.001 | -1.179 (-1.903, -0.456) | 0.002 |  |
|  | Model2 | Ref |  | -0.140 (-0.613, 0.333) | 0.554 | -0.834 (-1.445, -0.224) | 0.008 | -0.906 (-1.601, -0.211) | 0.012 |  |
|  | Model3 | Ref |  | -0.144 (-0.608, 0.321) | 0.535 | -0.650 (-1.307, 0.007) | 0.052 | -0.523 (-1.345, 0.298) | 0.205 |  |
|  | Model4 | Ref |  | -0.168 (-0.638, 0.301) | 0.472 | -0.585 (-1.228, 0.059) | 0.074 | -0.479 (-1.276, 0.319) | 0.232 | 0.158 |
| PhenoAge-acceleration | Model1 | Ref |  | -0.734 (-1.094, -0.375) | <0.001 | -1.447 (-1.833, -1.061) | <0.001 | -2.224 (-2.733, -1.715) | <0.001 |  |
|  | Model2 | Ref |  | -0.562 (-0.930, -0.193) | 0.004 | -1.139 (-1.514, -0.764) | <0.001 | -1.820 (-2.304, -1.336) | <0.001 |  |
|  | Model3 | Ref |  | -0.451 (-0.809, -0.092) | 0.015 | -0.764 (-1.136, -0.392) | <0.001 | -1.138 (-1.642, -0.634) | <0.001 |  |
|  | Model4 | Ref |  | -0.467 (-0.817, -0.118) | 0.010 | -0.720 (-1.083, -0.356) | <0.001 | -1.108 (-1.601, -0.615) | <0.001 | <0.001 |
| HD | Model1 | Ref |  | -0.056 (-0.099, -0.012) | 0.014 | -0.085 (-0.131, -0.040) | <0.001 | -0.095 (-0.151, -0.039) | 0.001 |  |
|  | Model2 | Ref |  | -0.043 (-0.088, 0.002) | 0.062 | -0.064 (-0.113, -0.015) | 0.011 | -0.068 (-0.127, -0.010) | 0.023 |  |
|  | Model3 | Ref |  | -0.031 (-0.077, 0.016) | 0.194 | -0.040 (-0.092, 0.013) | 0.132 | -0.036 (-0.101, 0.029) | 0.265 |  |
|  | Model4 | Ref |  | -0.033 (-0.079, 0.013) | 0.158 | -0.034 (-0.084, 0.016) | 0.176 | -0.032 (-0.095, 0.030) | 0.300 | 0.352 |

Abbreviations: KDM-acceleration, residual-based acceleration of Klemera and Doubal Model biological age (KDM-BA) relative to chronological age; PhenoAge-acceleration, residual-based acceleration of PhenoAge relative to chronological age; HD, homeostatic dysregulation.

• Model 1: adjustment for age, sex, and race.

• Model 2: Model 1 + adjustment for educational level, marital status, and poverty income ratio.

• Model 3: Model 2 + adjustment for body mass index, smoking status, alcohol consumption, physical activity level, daily energy intake, and supplement use.

• Model 4: Model 3 + adjustment for comorbidity (including hypertension,diabetes, cardiovascular diseases, and cancer).

Note: The trend test is based on the Model 4 and uses quartiles as continuous variables for analysis.

**Table S4. Associations between ln-transformed vitamin B1 intake and biological aging indicators**

| **Biological aging** | **Model** | **Q1** | | **Q2** | | **Q3** | | **Q4** | | **P for trend** |
| --- | --- | --- | --- | --- | --- | --- | --- | --- | --- | --- |
|  |  | **Ref** |  | **β (95% CI)** | **P value** | **β (95% CI)** | **P value** | **β (95% CI)** | **P value** |  |
| KDM-acceleration | Model1 | Ref |  | 0.151 (-0.446, 0.747) | 0.615 | -0.347 (-0.997, 0.303) | 0.290 | -0.798 (-1.426, -0.170) | 0.014 |  |
|  | Model2 | Ref |  | 0.228 (-0.364, 0.820) | 0.443 | -0.243 (-0.880, 0.394) | 0.447 | -0.601 (-1.185, -0.017) | 0.044 |  |
|  | Model3 | Ref |  | 0.299 (-0.238, 0.835) | 0.267 | -0.181 (-0.782, 0.420) | 0.546 | -0.335 (-0.896, 0.225) | 0.233 |  |
|  | Model4 | Ref |  | 0.307 (-0.230, 0.844) | 0.255 | -0.158 (-0.723, 0.407) | 0.575 | -0.259 (-0.820, 0.302) | 0.355 | 0.155 |
| PhenoAge-acceleration | Model1 | Ref |  | -0.645 (-1.036, -0.255) | 0.002 | -1.235 (-1.783, -0.688) | <0.001 | -1.780 (-2.284, -1.277) | <0.001 |  |
|  | Model2 | Ref |  | -0.528 (-0.928, -0.127) | 0.011 | -1.082 (-1.617, -0.546) | <0.001 | -1.475 (-1.948, -1.002) | <0.001 |  |
|  | Model3 | Ref |  | -0.418 (-0.797, -0.039) | 0.032 | -0.912 (-1.385, -0.439) | <0.001 | -1.027 (-1.467, -0.588) | <0.001 |  |
|  | Model4 | Ref |  | -0.412 (-0.793, -0.032) | 0.034 | -0.897 (-1.355, -0.439) | <0.001 | -0.976 (-1.433, -0.519) | <0.001 | <0.001 |
| HD | Model1 | Ref |  | -0.057 (-0.103, -0.012) | 0.015 | -0.085 (-0.138, -0.031) | 0.002 | -0.086 (-0.132, -0.040) | <0.001 |  |
|  | Model2 | Ref |  | -0.048 (-0.094, -0.002) | 0.041 | -0.072 (-0.125, -0.018) | 0.010 | -0.064 (-0.110, -0.019) | 0.006 |  |
|  | Model3 | Ref |  | -0.032 (-0.076, 0.013) | 0.160 | -0.054 (-0.108, -0.001) | 0.047 | -0.051 (-0.102, -0.001) | 0.047 |  |
|  | Model4 | Ref |  | -0.031 (-0.074, 0.013) | 0.158 | -0.052 (-0.102, -0.002) | 0.041 | -0.045 (-0.095, 0.006) | 0.082 | 0.089 |

Abbreviations: KDM-acceleration, residual-based acceleration of Klemera and Doubal Model biological age (KDM-BA) relative to chronological age; PhenoAge-acceleration, residual-based acceleration of PhenoAge relative to chronological age; HD, homeostatic dysregulation.

• Model 1: adjustment for age, sex, and race.

• Model 2: Model 1 + adjustment for educational level, marital status, and poverty income ratio.

• Model 3: Model 2 + adjustment for body mass index, smoking status, alcohol consumption, physical activity level, daily energy intake, and supplement use.

• Model 4: Model 3 + adjustment for comorbidity (including hypertension,diabetes, cardiovascular diseases, and cancer).

Note: The trend test is based on the Model 4 and uses quartiles as continuous variables for analysis.

**Table S5. Associations between ln-transformed vitamin B2 intake and biological aging indicators**

| **Biological aging** | **Model** | **Q1** | | **Q2** | | **Q3** | | **Q4** | | **P for trend** |
| --- | --- | --- | --- | --- | --- | --- | --- | --- | --- | --- |
|  |  | **Ref** |  | **β (95% CI)** | **P value** | **β (95% CI)** | **P value** | **β (95% CI)** | **P value** |  |
| KDM-acceleration | Model1 | Ref |  | -0.214 (-0.864, 0.437) | 0.513 | -0.439 (-1.061, 0.182) | 0.162 | -1.218 (-1.810, -0.626) | <0.001 |  |
|  | Model2 | Ref |  | -0.063 (-0.706, 0.579) | 0.844 | -0.258 (-0.866, 0.349) | 0.397 | -0.974 (-1.531, -0.417) | <0.001 |  |
|  | Model3 | Ref |  | -0.074 (-0.727, 0.579) | 0.820 | -0.223 (-0.830, 0.384) | 0.462 | -0.825 (-1.470, -0.180) | 0.014 |  |
|  | Model4 | Ref |  | -0.020 (-0.672, 0.631) | 0.950 | -0.110 (-0.695, 0.474) | 0.704 | -0.709 (-1.347, -0.070) | 0.031 | 0.018 |
| PhenoAge-acceleration | Model1 | Ref |  | -0.724 (-1.151, -0.298) | 0.001 | -1.061 (-1.546, -0.576) | <0.001 | -2.059 (-2.536, -1.581) | <0.001 |  |
|  | Model2 | Ref |  | -0.501 (-0.921, -0.081) | 0.020 | -0.793 (-1.253, -0.332) | 0.001 | -1.680 (-2.120, -1.240) | <0.001 |  |
|  | Model3 | Ref |  | -0.535 (-0.923, -0.147) | 0.008 | -0.756 (-1.167, -0.345) | <0.001 | -1.488 (-1.902, -1.074) | <0.001 |  |
|  | Model4 | Ref |  | -0.499 (-0.895, -0.103) | 0.015 | -0.681 (-1.088, -0.275) | 0.002 | -1.410 (-1.839, -0.982) | <0.001 | <0.001 |
| HD | Model1 | Ref |  | -0.053 (-0.098, -0.008) | 0.021 | -0.090 (-0.131, -0.049) | <0.001 | -0.095 (-0.138, -0.051) | <0.001 |  |
|  | Model2 | Ref |  | -0.037 (-0.081, 0.007) | 0.094 | -0.071 (-0.111, -0.030) | <0.001 | -0.068 (-0.111, -0.026) | 0.002 |  |
|  | Model3 | Ref |  | -0.021 (-0.062, 0.020) | 0.305 | -0.045 (-0.088, -0.003) | 0.038 | -0.045 (-0.095, 0.005) | 0.074 |  |
|  | Model4 | Ref |  | -0.016 (-0.058, 0.025) | 0.430 | -0.036 (-0.077, 0.006) | 0.093 | -0.035 (-0.084, 0.014) | 0.155 | 0.158 |

Abbreviations: KDM-acceleration, residual-based acceleration of Klemera and Doubal Model biological age (KDM-BA) relative to chronological age; PhenoAge-acceleration, residual-based acceleration of PhenoAge relative to chronological age; HD, homeostatic dysregulation.

• Model 1: adjustment for age, sex, and race.

• Model 2: Model 1 + adjustment for educational level, marital status, and poverty income ratio.

• Model 3: Model 2 + adjustment for body mass index, smoking status, alcohol consumption, physical activity level, daily energy intake, and supplement use.

• Model 4: Model 3 + adjustment for comorbidity (including hypertension,diabetes, cardiovascular diseases, and cancer).

Note: The trend test is based on the Model 4 and uses quartiles as continuous variables for analysis.

**Table S6. Associations between ln-transformed vitamin B3 intake and biological aging indicators**

| **Biological aging** | **Model** | **Q1** | | **Q2** | | **Q3** | | **Q4** | | **P for trend** |
| --- | --- | --- | --- | --- | --- | --- | --- | --- | --- | --- |
|  |  | **Ref** |  | **β (95% CI)** | **P value** | **β (95% CI)** | **P value** | **β (95% CI)** | **P value** |  |
| KDM-acceleration | Model1 | Ref |  | -0.011 (-0.718, 0.695) | 0.974 | -0.333 (-0.956, 0.290) | 0.288 | -0.619 (-1.186, -0.052) | 0.033 |  |
|  | Model2 | Ref |  | 0.150 (-0.551, 0.851) | 0.669 | -0.149 (-0.751, 0.453) | 0.621 | -0.369 (-0.896, 0.158) | 0.165 |  |
|  | Model3 | Ref |  | 0.237 (-0.502, 0.976) | 0.520 | 0.054 (-0.619, 0.726) | 0.873 | -0.009 (-0.738, 0.721) | 0.981 |  |
|  | Model4 | Ref |  | 0.209 (-0.526, 0.943) | 0.568 | 0.009 (-0.635, 0.654) | 0.977 | -0.008 (-0.742, 0.727) | 0.983 | 0.784 |
| PhenoAge-acceleration | Model1 | Ref |  | -0.432 (-0.849, -0.014) | 0.043 | -1.128 (-1.577, -0.679) | <0.001 | -1.733 (-2.180, -1.287) | <0.001 |  |
|  | Model2 | Ref |  | -0.195 (-0.590, 0.201) | 0.327 | -0.863 (-1.279, -0.447) | <0.001 | -1.351 (-1.734, -0.967) | <0.001 |  |
|  | Model3 | Ref |  | -0.099 (-0.507, 0.308) | 0.625 | -0.640 (-1.020, -0.259) | 0.002 | -0.925 (-1.279, -0.570) | <0.001 |  |
|  | Model4 | Ref |  | -0.119 (-0.514, 0.277) | 0.547 | -0.670 (-1.042, -0.297) | <0.001 | -0.924 (-1.291, -0.557) | <0.001 | <0.001 |
| HD | Model1 | Ref |  | -0.061 (-0.104, -0.017) | 0.008 | -0.103 (-0.146, -0.059) | <0.001 | -0.099 (-0.141, -0.057) | <0.001 |  |
|  | Model2 | Ref |  | -0.044 (-0.088, 0.001) | 0.054 | -0.083 (-0.127, -0.039) | <0.001 | -0.072 (-0.115, -0.030) | 0.001 |  |
|  | Model3 | Ref |  | -0.027 (-0.079, 0.025) | 0.304 | -0.056 (-0.110, -0.003) | 0.038 | -0.041 (-0.102, 0.021) | 0.187 |  |
|  | Model4 | Ref |  | -0.029 (-0.079, 0.020) | 0.241 | -0.060 (-0.112, -0.009) | 0.023 | -0.041 (-0.100, 0.019) | 0.176 | 0.137 |

Abbreviations: KDM-acceleration, residual-based acceleration of Klemera and Doubal Model biological age (KDM-BA) relative to chronological age; PhenoAge-acceleration, residual-based acceleration of PhenoAge relative to chronological age; HD, homeostatic dysregulation.

• Model 1: adjustment for age, sex, and race.

• Model 2: Model 1 + adjustment for educational level, marital status, and poverty income ratio.

• Model 3: Model 2 + adjustment for body mass index, smoking status, alcohol consumption, physical activity level, daily energy intake, and supplement use.

• Model 4: Model 3 + adjustment for comorbidity (including hypertension,diabetes, cardiovascular diseases, and cancer).

Note: The trend test is based on the Model 4 and uses quartiles as continuous variables for analysis.

**Table S7. Associations between ln-transformed vitamin B6 intake and biological aging indicators**

| **Biological aging** | **Model** | **Q1** | | **Q2** | | **Q3** | | **Q4** | | **P for trend** |
| --- | --- | --- | --- | --- | --- | --- | --- | --- | --- | --- |
|  |  | **Ref** |  | **β (95% CI)** | **P value** | **β (95% CI)** | **P value** | **β (95% CI)** | **P value** |  |
| KDM-acceleration | Model1 | Ref |  | -0.546 (-1.129, 0.036) | 0.066 | -0.769 (-1.388, -0.150) | 0.016 | -1.054 (-1.634, -0.474) | <0.001 |  |
|  | Model2 | Ref |  | -0.346 (-0.918, 0.226) | 0.230 | -0.557 (-1.150, 0.036) | 0.065 | -0.792 (-1.337, -0.248) | 0.005 |  |
|  | Model3 | Ref |  | -0.150 (-0.758, 0.457) | 0.620 | -0.226 (-0.837, 0.385) | 0.459 | -0.289 (-0.945, 0.366) | 0.378 |  |
|  | Model4 | Ref |  | -0.184 (-0.775, 0.407) | 0.533 | -0.151 (-0.737, 0.434) | 0.604 | -0.284 (-0.943, 0.374) | 0.388 | 0.445 |
| PhenoAge-acceleration | Model1 | Ref |  | -0.893 (-1.323, -0.463) | <0.001 | -1.693 (-2.165, -1.222) | <0.001 | -2.035 (-2.511, -1.559) | <0.001 |  |
|  | Model2 | Ref |  | -0.637 (-1.058, -0.216) | 0.004 | -1.410 (-1.849, -0.970) | <0.001 | -1.650 (-2.088, -1.213) | <0.001 |  |
|  | Model3 | Ref |  | -0.337 (-0.767, 0.093) | 0.121 | -0.963 (-1.367, -0.559) | <0.001 | -0.971 (-1.377, -0.565) | <0.001 |  |
|  | Model4 | Ref |  | -0.359 (-0.778, 0.059) | 0.090 | -0.913 (-1.296, -0.530) | <0.001 | -0.968 (-1.386, -0.549) | <0.001 | <0.001 |
| HD | Model1 | Ref |  | -0.069 (-0.111, -0.028) | 0.002 | -0.098 (-0.138, -0.058) | <0.001 | -0.098 (-0.138, -0.059) | <0.001 |  |
|  | Model2 | Ref |  | -0.050 (-0.090, -0.009) | 0.017 | -0.077 (-0.116, -0.037) | <0.001 | -0.071 (-0.110, -0.032) | <0.001 |  |
|  | Model3 | Ref |  | -0.027 (-0.068, 0.013) | 0.184 | -0.046 (-0.085, -0.007) | 0.023 | -0.044 (-0.090, 0.003) | 0.064 |  |
|  | Model4 | Ref |  | -0.030 (-0.070, 0.010) | 0.134 | -0.039 (-0.076, -0.003) | 0.036 | -0.043 (-0.090, 0.003) | 0.065 | 0.088 |

Abbreviations: KDM-acceleration, residual-based acceleration of Klemera and Doubal Model biological age (KDM-BA) relative to chronological age; PhenoAge-acceleration, residual-based acceleration of PhenoAge relative to chronological age; HD, homeostatic dysregulation.

• Model 1: adjustment for age, sex, and race.

• Model 2: Model 1 + adjustment for educational level, marital status, and poverty income ratio.

• Model 3: Model 2 + adjustment for body mass index, smoking status, alcohol consumption, physical activity level, daily energy intake, and supplement use.

• Model 4: Model 3 + adjustment for comorbidity (including hypertension,diabetes, cardiovascular diseases, and cancer).

Note: The trend test is based on the Model 4 and uses quartiles as continuous variables for analysis.

**Table S8. Associations between ln-transformed vitamin B9 intake and biological aging indicators**

| **Biological aging** | **Model** | **Q1** | | **Q2** | | **Q3** | | **Q4** | | **P for trend** |
| --- | --- | --- | --- | --- | --- | --- | --- | --- | --- | --- |
|  |  | **Ref** |  | **β (95% CI)** | **P value** | **β (95% CI)** | **P value** | **β (95% CI)** | **P value** |  |
| KDM-acceleration | Model1 | Ref |  | -0.453 (-1.131, 0.224) | 0.186 | -1.029 (-1.709, -0.350) | 0.004 | -1.621 (-2.256, -0.985) | <0.001 |  |
|  | Model2 | Ref |  | -0.326 (-1.003, 0.351) | 0.338 | -0.843 (-1.505, -0.181) | 0.014 | -1.385 (-2.012, -0.758) | <0.001 |  |
|  | Model3 | Ref |  | -0.313 (-0.983, 0.358) | 0.351 | -0.834 (-1.534, -0.135) | 0.021 | -1.166 (-1.961, -0.371) | 0.005 |  |
|  | Model4 | Ref |  | -0.255 (-0.943, 0.433) | 0.458 | -0.759 (-1.443, -0.075) | 0.031 | -1.140 (-1.936, -0.344) | 0.006 | 0.003 |
| PhenoAge-acceleration | Model1 | Ref |  | -0.704 (-1.131, -0.277) | 0.002 | -1.283 (-1.847, -0.718) | <0.001 | -2.109 (-2.629, -1.589) | <0.001 |  |
|  | Model2 | Ref |  | -0.506 (-0.919, -0.093) | 0.017 | -1.008 (-1.527, -0.489) | <0.001 | -1.734 (-2.209, -1.258) | <0.001 |  |
|  | Model3 | Ref |  | -0.317 (-0.641, 0.007) | 0.055 | -0.682 (-1.143, -0.220) | 0.005 | -1.114 (-1.587, -0.641) | <0.001 |  |
|  | Model4 | Ref |  | -0.278 (-0.618, 0.062) | 0.106 | -0.631 (-1.084, -0.178) | 0.008 | -1.097 (-1.582, -0.612) | <0.001 | <0.001 |
| HD | Model1 | Ref |  | -0.030 (-0.081, 0.020) | 0.231 | -0.086 (-0.128, -0.044) | <0.001 | -0.078 (-0.113, -0.042) | <0.001 |  |
|  | Model2 | Ref |  | -0.016 (-0.067, 0.034) | 0.520 | -0.066 (-0.108, -0.025) | 0.002 | -0.052 (-0.089, -0.016) | 0.006 |  |
|  | Model3 | Ref |  | 0.005 (-0.043, 0.052) | 0.836 | -0.037 (-0.081, 0.007) | 0.094 | -0.024 (-0.074, 0.026) | 0.335 |  |
|  | Model4 | Ref |  | 0.010 (-0.038, 0.058) | 0.676 | -0.031 (-0.072, 0.011) | 0.145 | -0.022 (-0.071, 0.027) | 0.370 | 0.197 |

Abbreviations: KDM-acceleration, residual-based acceleration of Klemera and Doubal Model biological age (KDM-BA) relative to chronological age; PhenoAge-acceleration, residual-based acceleration of PhenoAge relative to chronological age; HD, homeostatic dysregulation.

• Model 1: adjustment for age, sex, and race.

• Model 2: Model 1 + adjustment for educational level, marital status, and poverty income ratio.

• Model 3: Model 2 + adjustment for body mass index, smoking status, alcohol consumption, physical activity level, daily energy intake, and supplement use.

• Model 4: Model 3 + adjustment for comorbidity (including hypertension,diabetes, cardiovascular diseases, and cancer).

Note: The trend test is based on the Model 4 and uses quartiles as continuous variables for analysis.

**Table S9. Associations between ln-transformed vitamin B12 intake and biological aging indicators**

| **Biological aging** | **Model** | **Q1** | | **Q2** | | **Q3** | | **Q4** | | **P for trend** |
| --- | --- | --- | --- | --- | --- | --- | --- | --- | --- | --- |
|  |  | **Ref** |  | **β (95% CI)** | **P value** | **β (95% CI)** | **P value** | **β (95% CI)** | **P value** |  |
| KDM-acceleration | Model1 | Ref |  | -0.094 (-0.607, 0.419) | 0.715 | -0.370 (-1.035, 0.295) | 0.269 | -0.925 (-1.461, -0.388) | 0.001 |  |
|  | Model2 | Ref |  | -0.030 (-0.540, 0.480) | 0.907 | -0.283 (-0.934, 0.368) | 0.386 | -0.735 (-1.264, -0.206) | 0.007 |  |
|  | Model3 | Ref |  | -0.011 (-0.566, 0.544) | 0.967 | -0.216 (-0.889, 0.457) | 0.520 | -0.541 (-1.149, 0.067) | 0.080 |  |
|  | Model4 | Ref |  | -0.047 (-0.593, 0.500) | 0.864 | -0.203 (-0.850, 0.444) | 0.529 | -0.488 (-1.084, 0.107) | 0.105 | 0.097 |
| PhenoAge-acceleration | Model1 | Ref |  | -0.667 (-1.099, -0.235) | 0.003 | -1.249 (-1.774, -0.723) | <0.001 | -1.872 (-2.315, -1.429) | <0.001 |  |
|  | Model2 | Ref |  | -0.566 (-1.009, -0.123) | 0.013 | -1.115 (-1.624, -0.606) | <0.001 | -1.571 (-2.007, -1.135) | <0.001 |  |
|  | Model3 | Ref |  | -0.504 (-0.939, -0.069) | 0.024 | -0.970 (-1.436, -0.504) | <0.001 | -1.202 (-1.637, -0.768) | <0.001 |  |
|  | Model4 | Ref |  | -0.528 (-0.954, -0.102) | 0.017 | -0.961 (-1.409, -0.514) | <0.001 | -1.167 (-1.609, -0.726) | <0.001 | <0.001 |
| HD | Model1 | Ref |  | -0.061 (-0.104, -0.017) | 0.007 | -0.074 (-0.128, -0.021) | 0.007 | -0.091 (-0.135, -0.046) | <0.001 |  |
|  | Model2 | Ref |  | -0.052 (-0.097, -0.008) | 0.022 | -0.063 (-0.117, -0.009) | 0.023 | -0.069 (-0.116, -0.023) | 0.005 |  |
|  | Model3 | Ref |  | -0.038 (-0.084, 0.008) | 0.106 | -0.044 (-0.101, 0.013) | 0.129 | -0.058 (-0.114, -0.001) | 0.046 |  |
|  | Model4 | Ref |  | -0.041 (-0.086, 0.005) | 0.077 | -0.043 (-0.097, 0.012) | 0.121 | -0.053 (-0.109, 0.003) | 0.065 | 0.096 |

Abbreviations: KDM-acceleration, residual-based acceleration of Klemera and Doubal Model biological age (KDM-BA) relative to chronological age; PhenoAge-acceleration, residual-based acceleration of PhenoAge relative to chronological age; HD, homeostatic dysregulation.

• Model 1: adjustment for age, sex, and race.

• Model 2: Model 1 + adjustment for educational level, marital status, and poverty income ratio.

• Model 3: Model 2 + adjustment for body mass index, smoking status, alcohol consumption, physical activity level, daily energy intake, and supplement use.

• Model 4: Model 3 + adjustment for comorbidity (including hypertension,diabetes, cardiovascular diseases, and cancer).

Note: The trend test is based on the Model 4 and uses quartiles as continuous variables for analysis.

**Table S10. Associations between ln-transformed vitamin C intake and biological aging indicators**

| **Biological aging** | **Model** | **Q1** | | **Q2** | | **Q3** | | **Q4** | | **P for trend** |
| --- | --- | --- | --- | --- | --- | --- | --- | --- | --- | --- |
|  |  | **Ref** |  | **β (95% CI)** | **P value** | **β (95% CI)** | **P value** | **β (95% CI)** | **P value** |  |
| KDM-acceleration | Model1 | Ref |  | -1.102 (-1.729, -0.475) | <0.001 | -1.959 (-2.577, -1.341) | <0.001 | -2.186 (-2.842, -1.529) | <0.001 |  |
|  | Model2 | Ref |  | -0.938 (-1.544, -0.332) | 0.003 | -1.739 (-2.352, -1.126) | <0.001 | -1.942 (-2.592, -1.291) | <0.001 |  |
|  | Model3 | Ref |  | -0.837 (-1.406, -0.268) | 0.005 | -1.486 (-2.147, -0.826) | <0.001 | -1.586 (-2.381, -0.791) | <0.001 |  |
|  | Model4 | Ref |  | -0.735 (-1.301, -0.169) | 0.012 | -1.424 (-2.079, -0.770) | <0.001 | -1.513 (-2.294, -0.732) | <0.001 | <0.001 |
| PhenoAge-acceleration | Model1 | Ref |  | -1.097 (-1.580, -0.614) | <0.001 | -2.068 (-2.542, -1.594) | <0.001 | -2.544 (-3.036, -2.051) | <0.001 |  |
|  | Model2 | Ref |  | -0.834 (-1.278, -0.390) | <0.001 | -1.724 (-2.167, -1.282) | <0.001 | -2.142 (-2.602, -1.683) | <0.001 |  |
|  | Model3 | Ref |  | -0.501 (-0.889, -0.113) | 0.013 | -1.075 (-1.508, -0.641) | <0.001 | -1.323 (-1.803, -0.843) | <0.001 |  |
|  | Model4 | Ref |  | -0.432 (-0.809, -0.055) | 0.026 | -1.033 (-1.464, -0.602) | <0.001 | -1.274 (-1.749, -0.799) | <0.001 | <0.001 |
| HD | Model1 | Ref |  | -0.066 (-0.118, -0.013) | 0.016 | -0.101 (-0.145, -0.057) | <0.001 | -0.094 (-0.139, -0.050) | <0.001 |  |
|  | Model2 | Ref |  | -0.048 (-0.099, 0.003) | 0.066 | -0.079 (-0.123, -0.035) | <0.001 | -0.068 (-0.113, -0.022) | 0.004 |  |
|  | Model3 | Ref |  | -0.036 (-0.083, 0.012) | 0.141 | -0.060 (-0.106, -0.014) | 0.013 | -0.049 (-0.102, 0.004) | 0.068 |  |
|  | Model4 | Ref |  | -0.027 (-0.072, 0.019) | 0.244 | -0.054 (-0.098, -0.011) | 0.016 | -0.043 (-0.093, 0.007) | 0.093 | 0.070 |

Abbreviations: KDM-acceleration, residual-based acceleration of Klemera and Doubal Model biological age (KDM-BA) relative to chronological age; PhenoAge-acceleration, residual-based acceleration of PhenoAge relative to chronological age; HD, homeostatic dysregulation.

• Model 1: adjustment for age, sex, and race.

• Model 2: Model 1 + adjustment for educational level, marital status, and poverty income ratio.

• Model 3: Model 2 + adjustment for body mass index, smoking status, alcohol consumption, physical activity level, daily energy intake, and supplement use.

• Model 4: Model 3 + adjustment for comorbidity (including hypertension,diabetes, cardiovascular diseases, and cancer).

Note: The trend test is based on the Model 4 and uses quartiles as continuous variables for analysis.

**Table S11. Associations between ln-transformed vitamin D intake and biological aging indicators**

| **Biological aging** | **Model** | **Q1** | | **Q2** | | **Q3** | | **Q4** | | **P for trend** |
| --- | --- | --- | --- | --- | --- | --- | --- | --- | --- | --- |
|  |  | **Ref** |  | **β (95% CI)** | **P value** | **β (95% CI)** | **P value** | **β (95% CI)** | **P value** |  |
| KDM-acceleration | Model1 | Ref |  | 0.557 (0.064, 1.049) | 0.027 | -0.196 (-0.775, 0.383) | 0.501 | -0.111 (-0.605, 0.382) | 0.652 |  |
|  | Model2 | Ref |  | 0.638 (0.150, 1.126) | 0.011 | -0.085 (-0.642, 0.472) | 0.760 | 0.106 (-0.338, 0.551) | 0.634 |  |
|  | Model3 | Ref |  | 0.581 (0.109, 1.053) | 0.017 | 0.164 (-0.348, 0.677) | 0.520 | 0.498 (-0.008, 1.003) | 0.054 |  |
|  | Model4 | Ref |  | 0.600 (0.127, 1.073) | 0.014 | 0.186 (-0.307, 0.680) | 0.449 | 0.436 (-0.061, 0.934) | 0.084 | 0.246 |
| PhenoAge-acceleration | Model1 | Ref |  | -0.205 (-0.589, 0.179) | 0.290 | -1.049 (-1.454, -0.645) | <0.001 | -1.336 (-1.783, -0.889) | <0.001 |  |
|  | Model2 | Ref |  | -0.130 (-0.501, 0.240) | 0.483 | -0.914 (-1.284, -0.545) | <0.001 | -1.023 (-1.428, -0.619) | <0.001 |  |
|  | Model3 | Ref |  | -0.078 (-0.422, 0.265) | 0.647 | -0.515 (-0.871, -0.159) | 0.006 | -0.460 (-0.842, -0.078) | 0.020 |  |
|  | Model4 | Ref |  | -0.066 (-0.413, 0.281) | 0.704 | -0.500 (-0.854, -0.146) | 0.007 | -0.501 (-0.881, -0.122) | 0.011 | 0.003 |
| HD | Model1 | Ref |  | -0.007 (-0.049, 0.036) | 0.759 | -0.046 (-0.092, -0.000) | 0.048 | -0.046 (-0.086, -0.007) | 0.022 |  |
|  | Model2 | Ref |  | -0.002 (-0.045, 0.041) | 0.929 | -0.038 (-0.081, 0.006) | 0.089 | -0.026 (-0.065, 0.013) | 0.189 |  |
|  | Model3 | Ref |  | 0.007 (-0.036, 0.049) | 0.751 | -0.020 (-0.064, 0.023) | 0.351 | -0.017 (-0.065, 0.030) | 0.469 |  |
|  | Model4 | Ref |  | 0.008 (-0.034, 0.051) | 0.690 | -0.019 (-0.062, 0.025) | 0.395 | -0.023 (-0.070, 0.025) | 0.347 | 0.224 |

Abbreviations: KDM-acceleration, residual-based acceleration of Klemera and Doubal Model biological age (KDM-BA) relative to chronological age; PhenoAge-acceleration, residual-based acceleration of PhenoAge relative to chronological age; HD, homeostatic dysregulation.

• Model 1: adjustment for age, sex, and race.

• Model 2: Model 1 + adjustment for educational level, marital status, and poverty income ratio.

• Model 3: Model 2 + adjustment for body mass index, smoking status, alcohol consumption, physical activity level, daily energy intake, and supplement use.

• Model 4: Model 3 + adjustment for comorbidity (including hypertension,diabetes, cardiovascular diseases, and cancer).

Note: The trend test is based on the Model 4 and uses quartiles as continuous variables for analysis.

**Table S12. Associations between ln-transformed vitamin E intake and biological aging indicators**

| **Biological aging** | **Model** | **Q1** | | **Q2** | | **Q3** | | **Q4** | | **P for trend** |
| --- | --- | --- | --- | --- | --- | --- | --- | --- | --- | --- |
|  |  | **Ref** |  | **β (95% CI)** | **P value** | **β (95% CI)** | **P value** | **β (95% CI)** | **P value** |  |
| KDM-acceleration | Model1 | Ref |  | -0.394 (-0.996, 0.208) | 0.195 | -0.640 (-1.250, -0.031) | 0.040 | -0.791 (-1.439, -0.142) | 0.018 |  |
|  | Model2 | Ref |  | -0.192 (-0.778, 0.395) | 0.514 | -0.343 (-0.934, 0.247) | 0.248 | -0.436 (-1.084, 0.212) | 0.183 |  |
|  | Model3 | Ref |  | -0.141 (-0.695, 0.413) | 0.609 | -0.216 (-0.839, 0.407) | 0.488 | -0.142 (-0.916, 0.631) | 0.712 |  |
|  | Model4 | Ref |  | -0.130 (-0.677, 0.417) | 0.634 | -0.174 (-0.804, 0.455) | 0.579 | -0.138 (-0.897, 0.621) | 0.715 | 0.751 |
| PhenoAge-acceleration | Model1 | Ref |  | -0.943 (-1.291, -0.596) | <0.001 | -1.207 (-1.587, -0.828) | <0.001 | -1.789 (-2.208, -1.370) | <0.001 |  |
|  | Model2 | Ref |  | -0.664 (-1.012, -0.315) | <0.001 | -0.798 (-1.156, -0.440) | <0.001 | -1.284 (-1.677, -0.890) | <0.001 |  |
|  | Model3 | Ref |  | -0.541 (-0.899, -0.182) | 0.004 | -0.541 (-0.893, -0.188) | 0.004 | -0.864 (-1.300, -0.427) | <0.001 |  |
|  | Model4 | Ref |  | -0.533 (-0.895, -0.171) | 0.005 | -0.513 (-0.875, -0.151) | 0.007 | -0.861 (-1.290, -0.431) | <0.001 | 0.001 |
| HD | Model1 | Ref |  | -0.069 (-0.114, -0.023) | 0.004 | -0.098 (-0.143, -0.053) | <0.001 | -0.116 (-0.159, -0.074) | <0.001 |  |
|  | Model2 | Ref |  | -0.049 (-0.095, -0.004) | 0.035 | -0.069 (-0.112, -0.026) | 0.002 | -0.082 (-0.126, -0.039) | <0.001 |  |
|  | Model3 | Ref |  | -0.033 (-0.072, 0.007) | 0.103 | -0.038 (-0.082, 0.005) | 0.083 | -0.043 (-0.091, 0.006) | 0.084 |  |
|  | Model4 | Ref |  | -0.032 (-0.070, 0.007) | 0.107 | -0.035 (-0.077, 0.008) | 0.107 | -0.042 (-0.089, 0.004) | 0.075 | 0.144 |

Abbreviations: KDM-acceleration, residual-based acceleration of Klemera and Doubal Model biological age (KDM-BA) relative to chronological age; PhenoAge-acceleration, residual-based acceleration of PhenoAge relative to chronological age; HD, homeostatic dysregulation.

• Model 1: adjustment for age, sex, and race.

• Model 2: Model 1 + adjustment for educational level, marital status, and poverty income ratio.

• Model 3: Model 2 + adjustment for body mass index, smoking status, alcohol consumption, physical activity level, daily energy intake, and supplement use.

• Model 4: Model 3 + adjustment for comorbidity (including hypertension,diabetes, cardiovascular diseases, and cancer).

Note: The trend test is based on the Model 4 and uses quartiles as continuous variables for analysis.

**Table S13. Associations between ln-transformed vitamin K intake and biological aging indicators**

| **Biological aging** | **Model** | **Q1** | | **Q2** | | **Q3** | | **Q4** | | **P for trend** |
| --- | --- | --- | --- | --- | --- | --- | --- | --- | --- | --- |
|  |  | **Ref** |  | **β (95% CI)** | **P value** | **β (95% CI)** | **P value** | **β (95% CI)** | **P value** |  |
| KDM-acceleration | Model1 | Ref |  | -0.606 (-1.137, -0.075) | 0.026 | -0.963 (-1.592, -0.334) | 0.003 | -1.312 (-1.875, -0.748) | <0.001 |  |
|  | Model2 | Ref |  | -0.468 (-0.987, 0.052) | 0.076 | -0.735 (-1.328, -0.142) | 0.016 | -0.953 (-1.479, -0.428) | <0.001 |  |
|  | Model3 | Ref |  | -0.455 (-0.941, 0.030) | 0.065 | -0.692 (-1.315, -0.069) | 0.030 | -0.626 (-1.214, -0.037) | 0.038 |  |
|  | Model4 | Ref |  | -0.459 (-0.960, 0.043) | 0.072 | -0.604 (-1.200, -0.008) | 0.047 | -0.507 (-1.080, 0.066) | 0.081 | 0.131 |
| PhenoAge-acceleration | Model1 | Ref |  | -1.014 (-1.339, -0.689) | <0.001 | -1.167 (-1.671, -0.662) | <0.001 | -2.152 (-2.574, -1.730) | <0.001 |  |
|  | Model2 | Ref |  | -0.803 (-1.105, -0.502) | <0.001 | -0.828 (-1.292, -0.363) | <0.001 | -1.612 (-1.981, -1.243) | <0.001 |  |
|  | Model3 | Ref |  | -0.583 (-0.871, -0.295) | <0.001 | -0.459 (-0.915, -0.004) | 0.048 | -0.954 (-1.317, -0.592) | <0.001 |  |
|  | Model4 | Ref |  | -0.585 (-0.876, -0.295) | <0.001 | -0.400 (-0.840, 0.040) | 0.073 | -0.875 (-1.222, -0.527) | <0.001 | <0.001 |
| HD | Model1 | Ref |  | -0.031 (-0.067, 0.005) | 0.093 | -0.065 (-0.109, -0.021) | 0.004 | -0.099 (-0.139, -0.059) | <0.001 |  |
|  | Model2 | Ref |  | -0.014 (-0.050, 0.021) | 0.416 | -0.039 (-0.080, 0.003) | 0.066 | -0.061 (-0.102, -0.021) | 0.004 |  |
|  | Model3 | Ref |  | 0.005 (-0.029, 0.039) | 0.763 | -0.009 (-0.045, 0.027) | 0.613 | -0.026 (-0.065, 0.013) | 0.185 |  |
|  | Model4 | Ref |  | 0.005 (-0.031, 0.040) | 0.786 | -0.001 (-0.037, 0.035) | 0.935 | -0.016 (-0.054, 0.023) | 0.415 | 0.361 |

Abbreviations: KDM-acceleration, residual-based acceleration of Klemera and Doubal Model biological age (KDM-BA) relative to chronological age; PhenoAge-acceleration, residual-based acceleration of PhenoAge relative to chronological age; HD, homeostatic dysregulation.

• Model 1: adjustment for age, sex, and race.

• Model 2: Model 1 + adjustment for educational level, marital status, and poverty income ratio.

• Model 3: Model 2 + adjustment for body mass index, smoking status, alcohol consumption, physical activity level, daily energy intake, and supplement use.

• Model 4: Model 3 + adjustment for comorbidity (including hypertension,diabetes, cardiovascular diseases, and cancer).

Note: The trend test is based on the Model 4 and uses quartiles as continuous variables for analysis.

**Table S14. Stratified analysis of associations between ln-transformed total vitamin and biological aging indicators**

| **Subgroup** | **KDM-acceleration** | | | **PhenoAge-acceleration** | | | **HD** | | |
| --- | --- | --- | --- | --- | --- | --- | --- | --- | --- |
|  | **β (95% CI)** | **P_value_** | **P_interaction_** | **β (95% CI)** | **P_value_** | **P_interaction_** | **β (95% CI)** | **P_value_** | **P_interaction_** |
| **Age** |  |  |  |  |  |  |  |  |  |
| <60 | -0.568 (-1.066, -0.070) | 0.027 | 0.327 | -0.795 (-1.087, -0.503) | <0.001 | 0.257 | -0.019 (-0.059, 0.021) | 0.347 | 0.293 |
| ≥60 | -0.554 (-1.202, 0.095) | 0.092 |  | -0.531 (-0.902, -0.159) | 0.006 |  | -0.031 (-0.063, 0.002) | 0.063 |  |
| **Sex** |  |  |  |  |  |  |  |  |  |
| Female | -0.212 (-0.676, 0.253) | 0.363 | 0.002 | -0.520 (-0.837, -0.202) | 0.002 | 0.746 | -0.006 (-0.034, 0.023) | 0.687 | 0.915 |
| Male | -1.090 (-1.735, -0.446) | 0.001 |  | -0.805 (-1.145, -0.464) | <0.001 |  | -0.040 (-0.098, 0.017) | 0.162 |  |
| **Race** |  |  |  |  |  |  |  |  |  |
| Mexican American | 0.657 (-0.352, 1.665) | 0.195 | 0.158 | 0.061 (-0.554, 0.675) | 0.843 | 0.312 | -0.008 (-0.071, 0.055) | 0.793 | 0.605 |
| Non-Hispanic Black | -0.416 (-1.425, 0.593) | 0.409 |  | -0.790 (-1.484, -0.097) | 0.027 |  | -0.058 (-0.137, 0.021) | 0.147 |  |
| Non-Hispanic White | -0.541 (-1.059, -0.024) | 0.041 |  | -0.715 (-1.038, -0.391) | <0.001 |  | -0.008 (-0.038, 0.022) | 0.602 |  |
| Other Race | -1.429 (-2.955, 0.097) | 0.066 |  | -0.937 (-1.646, -0.228) | 0.011 |  | -0.096 (-0.237, 0.045) | 0.175 |  |
| **Educational level** |  |  |  |  |  |  |  |  |  |
| <High school | -1.416 (-2.878, 0.047) | 0.057 | 0.151 | -1.292 (-2.030, -0.553) | 0.001 | 0.081 | -0.077 (-0.170, 0.016) | 0.102 | 0.317 |
| >High school | -0.741 (-1.278, -0.205) | 0.008 |  | -0.646 (-0.962, -0.329) | <0.001 |  | -0.017 (-0.055, 0.021) | 0.369 |  |
| High school | -0.113 (-0.695, 0.469) | 0.698 |  | -0.680 (-1.089, -0.271) | 0.002 |  | -0.030 (-0.073, 0.013) | 0.165 |  |
| **Marital status** |  |  |  |  |  |  |  |  |  |
| Coupled | -0.521 (-0.945, -0.096) | 0.017 | 0.984 | -0.651 (-0.930, -0.371) | <0.001 | 0.887 | -0.010 (-0.037, 0.017) | 0.447 | 0.727 |
| Single/Separated | -0.678 (-1.376, 0.020) | 0.057 |  | -0.790 (-1.230, -0.351) | <0.001 |  | -0.047 (-0.113, 0.019) | 0.155 |  |
| **PIR** |  |  |  |  |  |  |  |  |  |
| <1.0 | -0.460 (-1.490, 0.571) | 0.373 | 0.884 | -0.635 (-1.390, 0.120) | 0.097 | 0.895 | -0.076 (-0.161, 0.009) | 0.078 | 0.195 |
| 1.0~3.0 | -0.756 (-1.506, -0.005) | 0.049 |  | -0.724 (-1.147, -0.301) | 0.001 |  | -0.060 (-0.116, -0.004) | 0.037 |  |
| ≥3.0 | -0.476 (-0.998, 0.047) | 0.073 |  | -0.692 (-0.989, -0.396) | <0.001 |  | 0.009 (-0.026, 0.045) | 0.601 |  |
| **BMI (kg/m^2^)** |  |  |  |  |  |  |  |  |  |
| <25 | -0.616 (-1.205, -0.027) | 0.041 | 0.051 | -0.908 (-1.255, -0.561) | <0.001 | 0.088 | -0.039 (-0.086, 0.008) | 0.099 | 0.775 |
| 25~30 | -1.001 (-1.817, -0.185) | 0.017 |  | -0.784 (-1.220, -0.347) | <0.001 |  | -0.032 (-0.097, 0.032) | 0.316 |  |
| ≥30 | -0.008 (-0.593, 0.576) | 0.977 |  | -0.368 (-0.671, -0.065) | 0.019 |  | -0.001 (-0.031, 0.029) | 0.966 |  |
| **Smoking status** |  |  |  |  |  |  |  |  |  |
| Current | -0.205 (-0.945, 0.534) | 0.578 | 0.520 | -0.786 (-1.381, -0.191) | 0.011 | 0.450 | -0.022 (-0.078, 0.035) | 0.444 | 0.585 |
| Former | -0.419 (-1.139, 0.302) | 0.248 |  | -0.476 (-0.881, -0.071) | 0.022 |  | -0.013 (-0.064, 0.039) | 0.621 |  |
| Never | -0.755 (-1.327, -0.182) | 0.011 |  | -0.762 (-1.062, -0.462) | <0.001 |  | -0.028 (-0.071, 0.015) | 0.193 |  |
| **Alcohol consumption** |  |  |  |  |  |  |  |  |  |
| No | -0.423 (-0.993, 0.147) | 0.142 | 0.099 | -0.617 (-0.955, -0.278) | <0.001 | 0.761 | -0.033 (-0.075, 0.009) | 0.118 | 0.359 |
| Yes | -0.905 (-1.544, -0.265) | 0.007 |  | -0.848 (-1.203, -0.493) | <0.001 |  | -0.002 (-0.043, 0.040) | 0.926 |  |
| **Physical activity level** |  |  |  |  |  |  |  |  |  |
| Inactive | -0.853 (-1.444, -0.263) | 0.006 | 0.601 | -0.826 (-1.198, -0.454) | <0.001 | 0.554 | -0.033 (-0.081, 0.014) | 0.162 | 0.166 |
| Moderate | -0.377 (-1.114, 0.361) | 0.309 |  | -0.720 (-1.163, -0.278) | 0.002 |  | -0.034 (-0.082, 0.014) | 0.160 |  |
| Active | -0.505 (-2.542, 1.532) | 0.619 |  | -1.145 (-1.855, -0.435) | 0.002 |  | -0.032 (-0.142, 0.078) | 0.558 |  |
| Highly active | -0.272 (-1.208, 0.664) | 0.561 |  | -0.271 (-0.767, 0.225) | 0.277 |  | 0.012 (-0.039, 0.063) | 0.642 |  |
| **Daily energy intake (kcal/day)** | | | | | | | | | |
| <1500 | -0.854 (-1.430, -0.278) | 0.005 | 0.346 | -0.773 (-1.157, -0.389) | <0.001 | 0.264 | -0.068 (-0.098, -0.039) | <0.001 | 0.022 |
| 1500-2500 | -0.206 (-0.724, 0.311) | 0.425 |  | -0.537 (-0.872, -0.201) | 0.002 |  | 0.015 (-0.014, 0.045) | 0.302 |  |
| ≥2500 | -1.024 (-2.006, -0.042) | 0.041 |  | -0.824 (-1.306, -0.341) | 0.001 |  | -0.037 (-0.139, 0.065) | 0.466 |  |
| **Supplement use** |  |  |  |  |  |  |  |  |  |
| No | -0.938 (-1.756, -0.120) | 0.026 | 0.234 | -1.078 (-1.652, -0.504) | <0.001 | 0.132 | -0.050 (-0.102, 0.003) | 0.062 | 0.296 |
| Yes | -0.426 (-0.955, 0.104) | 0.112 |  | -0.565 (-0.868, -0.261) | <0.001 |  | -0.013 (-0.051, 0.024) | 0.471 |  |
| **Comorbidity** |  |  |  |  |  |  |  |  |  |
| No | -0.407 (-0.861, 0.048) | 0.078 | 0.100 | -0.734 (-1.024, -0.445) | <0.001 | 0.848 | -0.007 (-0.033, 0.019) | 0.604 | 0.077 |
| Yes | -0.774 (-1.505, -0.042) | 0.039 |  | -0.699 (-1.068, -0.331) | <0.001 |  | -0.044 (-0.096, 0.007) | 0.088 |  |

Abbreviations: BMI, body mass index; PIR, poverty-income ratio; KDM-acceleration, residual-based acceleration of Klemera and Doubal Model biological age (KDM-BA) relative to chronological age; PhenoAge-acceleration, residual-based acceleration of PhenoAge relative to chronological age; HD, homeostatic dysregulation.

Vitamin intake was used as continuous variables for analysis.

Model adjusted for age, sex, race, educational level, marital status, PIR, BMI, smoking status, alcohol consumption, physical activity level, daily energy intake, supplement use, and comorbidity.

**Table S15. Joint effects of 11 vitamins on biological aging indicators by quantile g-computation model**

| **Outcomes** | **Joint effects** | **Standard error** | **P** |
| --- | --- | --- | --- |
| KDM-acceleration | -0.7649 | 0.1833 | <0.0001 |
| PhenoAge-acceleration | -0.8286 | 0.0994 | <0.0001 |
| HD | -0.0590 | 0.0138 | <0.0001 |

**Table S16. Weights and individual effects of each vitamin on KDM-acceleration by quantile g-computation model**

| **Vitamin** | **Weights** | **Single Effects** | **Direction** |
| --- | --- | --- | --- |
| Vitamin C | 0.2152 | -0.7124 | Negative |
| Vitamin B9 | 0.1233 | -0.4082 | Negative |
| Vitamin B2 | 0.1131 | -0.3744 | Negative |
| Vitamin B1 | 0.1003 | -0.3320 | Negative |
| Vitamin B12 | 0.0902 | 0.2986 | Positive |
| Vitamin B3 | 0.0882 | 0.2920 | Positive |
| Vitamin B6 | 0.0788 | 0.2608 | Positive |
| Vitamin A | 0.0636 | -0.2104 | Negative |
| Vitamin D | 0.0608 | 0.2013 | Positive |
| Vitamin K | 0.0362 | 0.1200 | Positive |
| Vitamin E | 0.0301 | 0.0997 | Positive |

**Table S17. Weights and individual effects of each vitamin on PhenoAge-acceleration by quantile g-computation model**

| **Vitamin** | **Weights** | **Single Effects** | **Direction** |
| --- | --- | --- | --- |
| Vitamin C | 0.2758 | -0.4022 | Negative |
| Vitamin B2 | 0.2385 | -0.3477 | Negative |
| Vitamin A | 0.1562 | -0.2278 | Negative |
| Vitamin D | 0.0897 | 0.1307 | Positive |
| Vitamin B1 | 0.0709 | 0.1034 | Positive |
| Vitamin B6 | 0.0416 | 0.0607 | Positive |
| Vitamin B12 | 0.0393 | -0.0573 | Negative |
| Vitamin B3 | 0.0341 | -0.0497 | Negative |
| Vitamin B9 | 0.0292 | -0.0426 | Negative |
| Vitamin E | 0.0137 | 0.0199 | Positive |
| Vitamin K | 0.0110 | -0.0161 | Negative |

**Table S18. Weights and individual effects of each vitamin on HD by quantile g-computation model**

| **Vitamin** | **Weights** | **Single Effects** | **Direction** |
| --- | --- | --- | --- |
| Vitamin C | 0.2898 | -0.0243 | Negative |
| Vitamin B2 | 0.1713 | -0.0143 | Negative |
| Vitamin B1 | 0.1680 | -0.0141 | Negative |
| Vitamin A | 0.0901 | -0.0075 | Negative |
| Vitamin B12 | 0.0816 | 0.0068 | Positive |
| Vitamin K | 0.0521 | -0.0044 | Negative |
| Vitamin B3 | 0.0516 | -0.0043 | Negative |
| Vitamin B9 | 0.0411 | 0.0034 | Positive |
| Vitamin E | 0.0254 | 0.0021 | Positive |
| Vitamin B6 | 0.0169 | -0.0014 | Negative |
| Vitamin D | 0.0122 | -0.0010 | Negative |

**Table S19. Associations between dietary-only vitamin intake and biological aging indicators (sensitivity analysis)**

| **Biological aging** | **Model** | **Q1** | | **Q2** | | **Q3** | | **Q4** | | **P for trend** |
| --- | --- | --- | --- | --- | --- | --- | --- | --- | --- | --- |
|  |  | **Ref** |  | **β (95% CI)** | **P value** | **β (95% CI)** | **P value** | **β (95% CI)** | **P value** |  |
| KDM-acceleration | Model1 | Ref |  | -0.735 (-1.358, -0.113) | 0.022 | -1.646 (-2.369, -0.923) | <0.001 | -2.191 (-2.769, -1.614) | <0.001 |  |
|  | Model2 | Ref |  | -0.574 (-1.185, 0.036) | 0.065 | -1.423 (-2.131, -0.715) | <0.001 | -1.910 (-2.465, -1.356) | <0.001 |  |
|  | Model3 | Ref |  | -0.535 (-1.166, 0.096) | 0.095 | -1.193 (-1.925, -0.462) | 0.002 | -1.581 (-2.265, -0.897) | <0.001 |  |
|  | Model4 | Ref |  | -0.519 (-1.146, 0.109) | 0.103 | -1.113 (-1.828, -0.398) | 0.003 | -1.539 (-2.212, -0.865) | <0.001 | <0.001 |
| PhenoAge-acceleration | Model1 | Ref |  | -1.020 (-1.418, -0.622) | <0.001 | -1.930 (-2.378, -1.481) | <0.001 | -2.576 (-3.023, -2.129) | <0.001 |  |
|  | Model2 | Ref |  | -0.771 (-1.152, -0.390) | <0.001 | -1.584 (-2.003, -1.165) | <0.001 | -2.147 (-2.567, -1.727) | <0.001 |  |
|  | Model3 | Ref |  | -0.486 (-0.875, -0.097) | 0.016 | -1.023 (-1.456, -0.591) | <0.001 | -1.403 (-1.879, -0.928) | <0.001 |  |
|  | Model4 | Ref |  | -0.475 (-0.855, -0.096) | 0.016 | -0.969 (-1.403, -0.536) | <0.001 | -1.375 (-1.828, -0.922) | <0.001 | <0.001 |
| HD | Model1 | Ref |  | -0.075 (-0.129, -0.021) | 0.007 | -0.117 (-0.166, -0.069) | <0.001 | -0.138 (-0.185, -0.092) | <0.001 |  |
|  | Model2 | Ref |  | -0.059 (-0.111, -0.006) | 0.030 | -0.095 (-0.143, -0.047) | <0.001 | -0.111 (-0.156, -0.067) | <0.001 |  |
|  | Model3 | Ref |  | -0.043 (-0.096, 0.010) | 0.112 | -0.069 (-0.119, -0.019) | 0.008 | -0.079 (-0.128, -0.029) | 0.003 |  |
|  | Model4 | Ref |  | -0.042 (-0.093, 0.010) | 0.112 | -0.062 (-0.109, -0.014) | 0.012 | -0.075 (-0.123, -0.028) | 0.003 | 0.001 |

Abbreviations: KDM-acceleration, residual-based acceleration of Klemera and Doubal Model biological age (KDM-BA) relative to chronological age; PhenoAge-acceleration, residual-based acceleration of PhenoAge relative to chronological age; HD, homeostatic dysregulation.

• Model 1: adjustment for age, sex, and race.

• Model 2: Model 1 + adjustment for educational level, marital status, and poverty income ratio.

• Model 3: Model 2 + adjustment for BMI, smoking status, alcohol consumption, physical activity level, daily energy intake, and supplement use.

• Model 4: Model 3 + adjustment for comorbidity (including hypertension,diabetes, cardiovascular diseases, and cancer).

Note: The trend test is based on the Model 4 and uses quartiles as continuous variables for analysis.

**Figure S1. Flowchart of the participants’ selection from NHANES**

**NHANES 2007-2018**

**(N=59842)**

**Adults without pregnancy**

**(N=36208)**

**Participants with plausible daily energy intake (N=31901)**

**Participants with complete vitamin intakes (N=27801)**

**Missing data about vitamin intakes (N=4100)**

**Missing data about biomarkers of biological aging (N=10275)**

**Participants with complete biomarkers of biological aging (N=17526)**

**Age < 18 years old (N=23262)**

**or pregnant women (N=372)**

**Implausible data about energy intake (N=4307)**

**Missing data about covariates (N=2476)**

**Final participants**

**(N=15050)**

NHANES, National Health and Nutrition Examination Survey.

**Figure S2. Forest plot of associations between ln-transformed total vitamin intake and biological aging indicators**


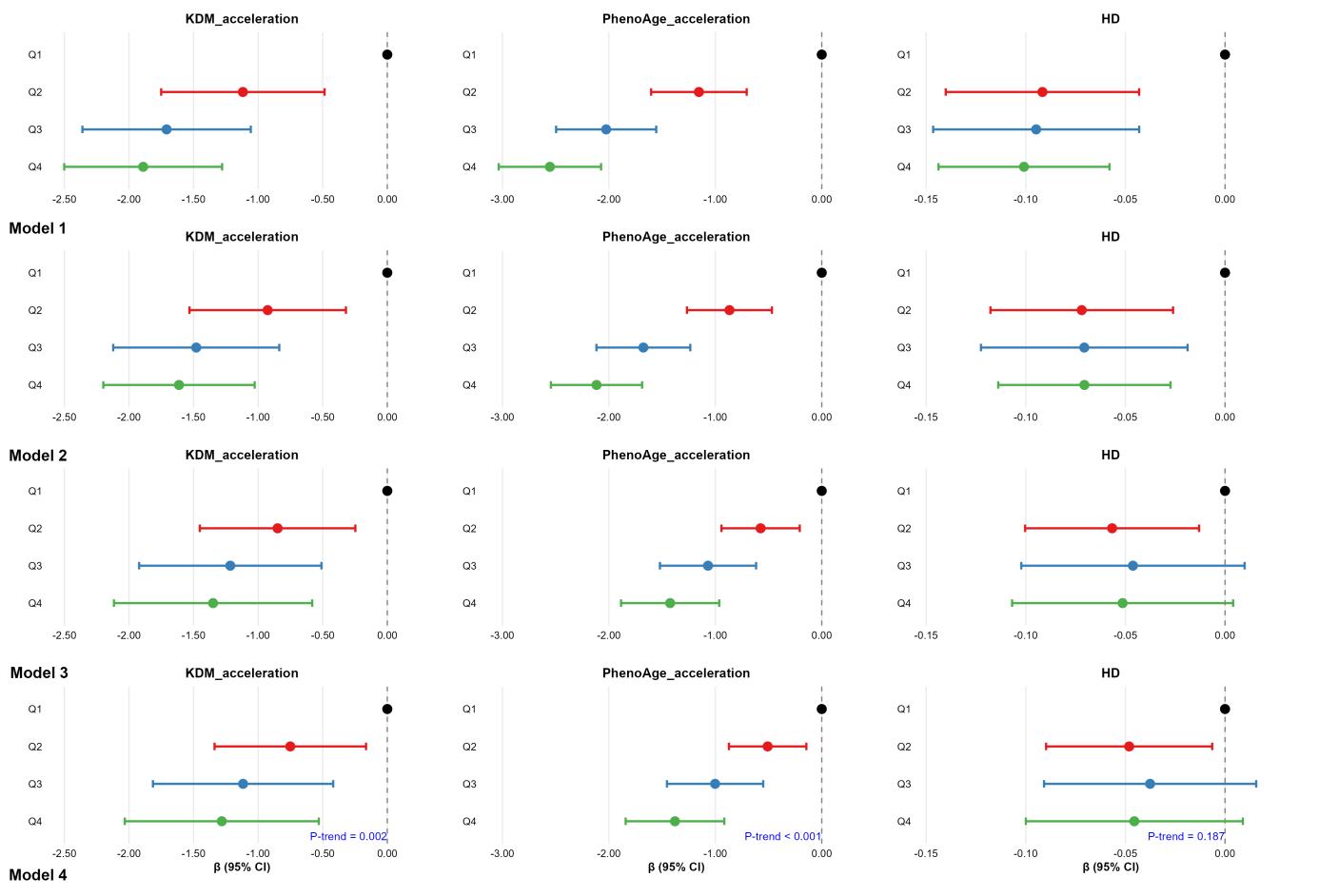


Abbreviations: KDM-acceleration, residual-based acceleration of Klemera and Doubal Model biological age (KDM-BA) relative to chronological age; PhenoAge-acceleration, residual-based acceleration of PhenoAge relative to chronological age; HD, homeostatic dysregulation.

• Model 1: adjustment for age, sex, and race.

• Model 2: Model 1 + adjustment for educational level, marital status, and poverty income ratio.

• Model 3: Model 2 + adjustment for body mass index, smoking status, alcohol consumption, physical activity level, daily energy intake, and supplement use.

• Model 4: Model 3 + adjustment for comorbidity (including hypertension,diabetes, cardiovascular diseases, and cancer).

Note: The trend test is based on the Model 4 and uses quartiles as continuous variables for analysis.

**Figure S3. Associations between ln-transformed total vitamin intake and biological aging indicators by restricted cubic spline**

**A.KDM-acceleration**


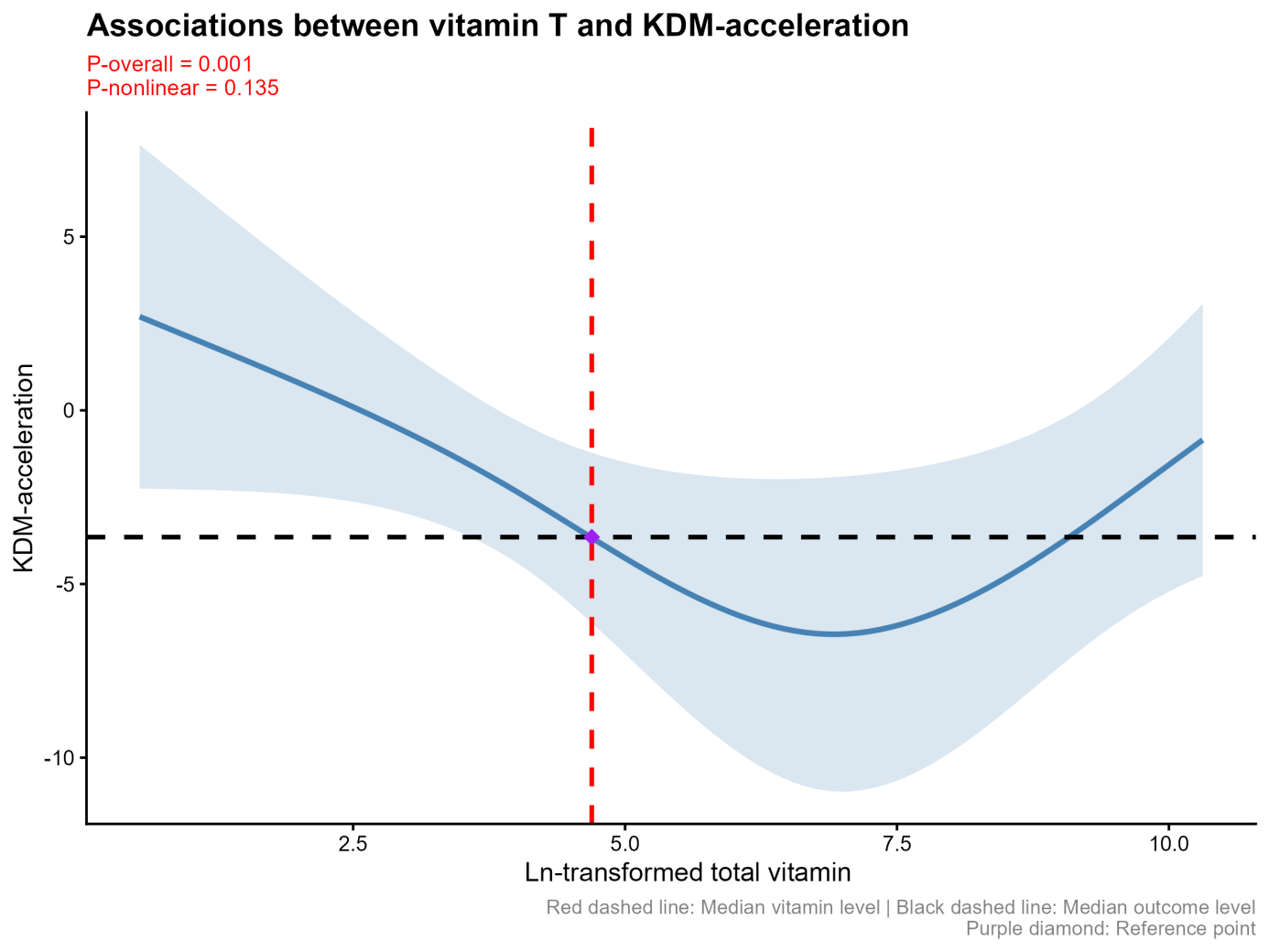


**B.PhenoAge-acceleration**


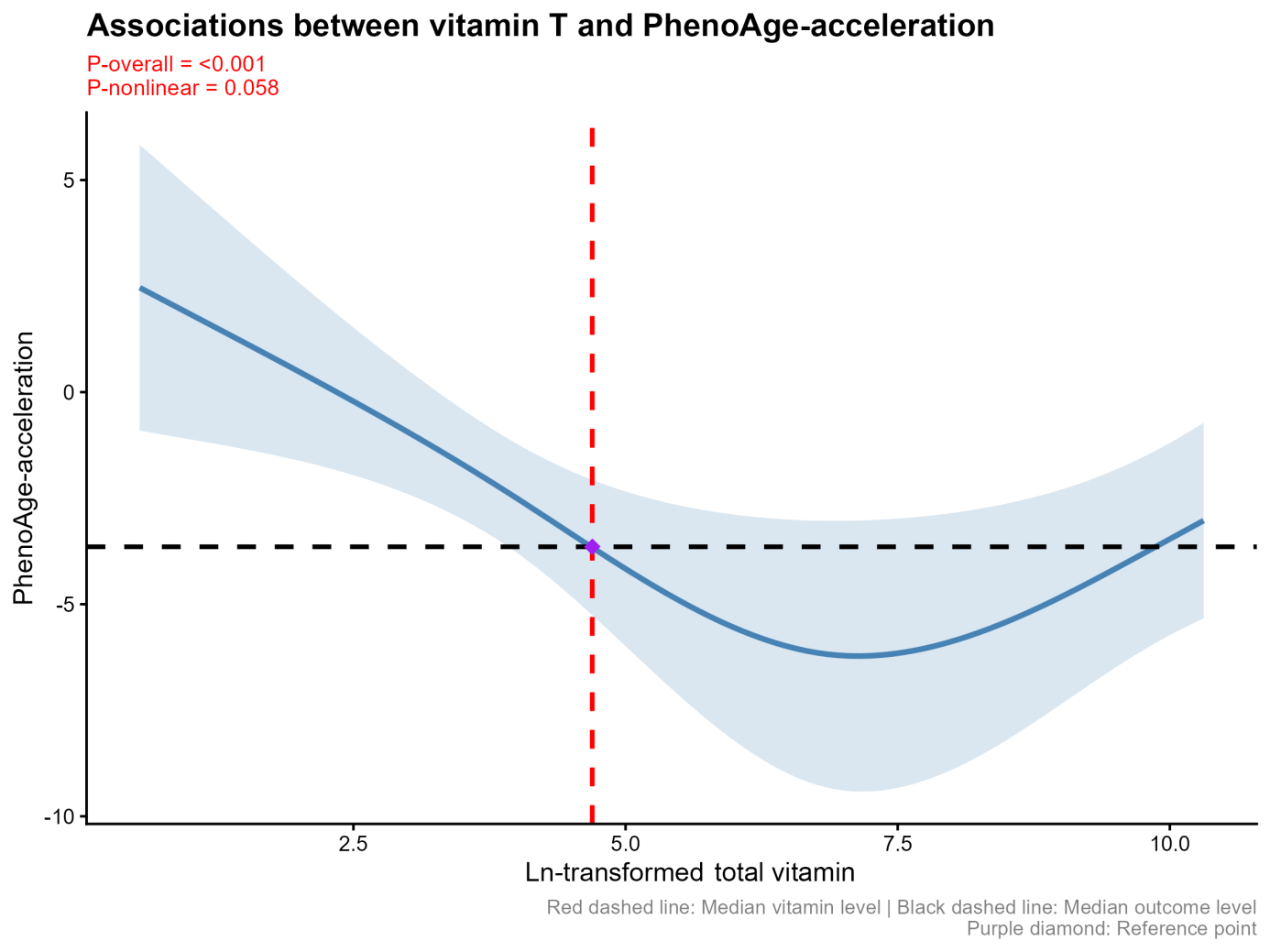


**C.HD**


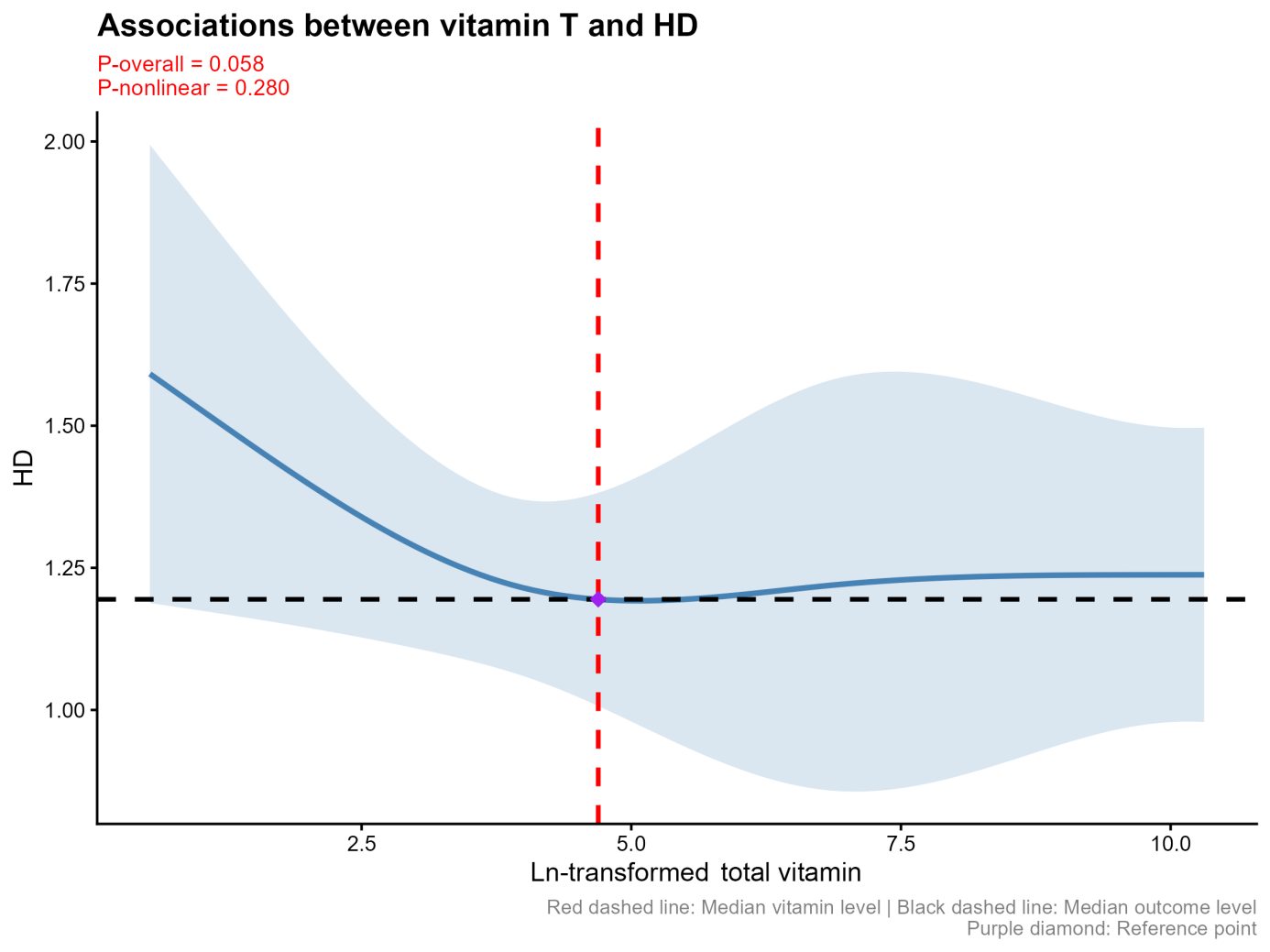


Abbreviations: KDM-acceleration, residual-based acceleration of Klemera and Doubal Model biological age (KDM-BA) relative to chronological age; PhenoAge-acceleration, residual-based acceleration of PhenoAge relative to chronological age; HD, homeostatic dysregulation.

Model adjusted for age, sex, race, educational level, marital status, poverty-income ratio, body mass index, smoking status, alcohol consumption, physical activity level, daily energy intake, supplement use, and comorbidity.

Red dashed line: Median vitamin level. Black dashed line: Median outcome level. Purple diamond: Reference point.

**Figure S4. Associations between ln-transformed vitamin A intake and biological aging indicators by restricted cubic spline**

**A.KDM-acceleration**

**
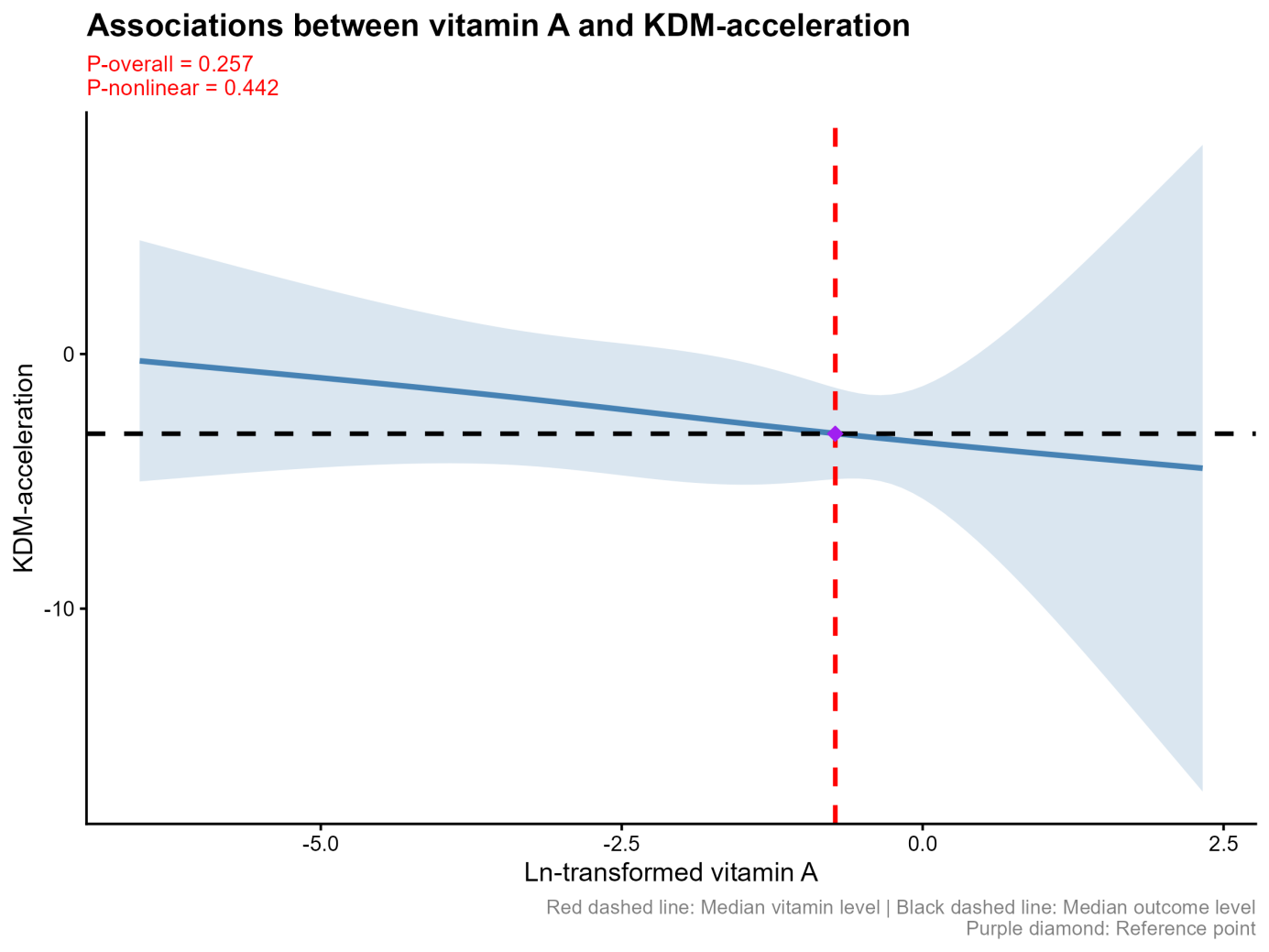
**

**B.PhenoAge-acceleration**

**
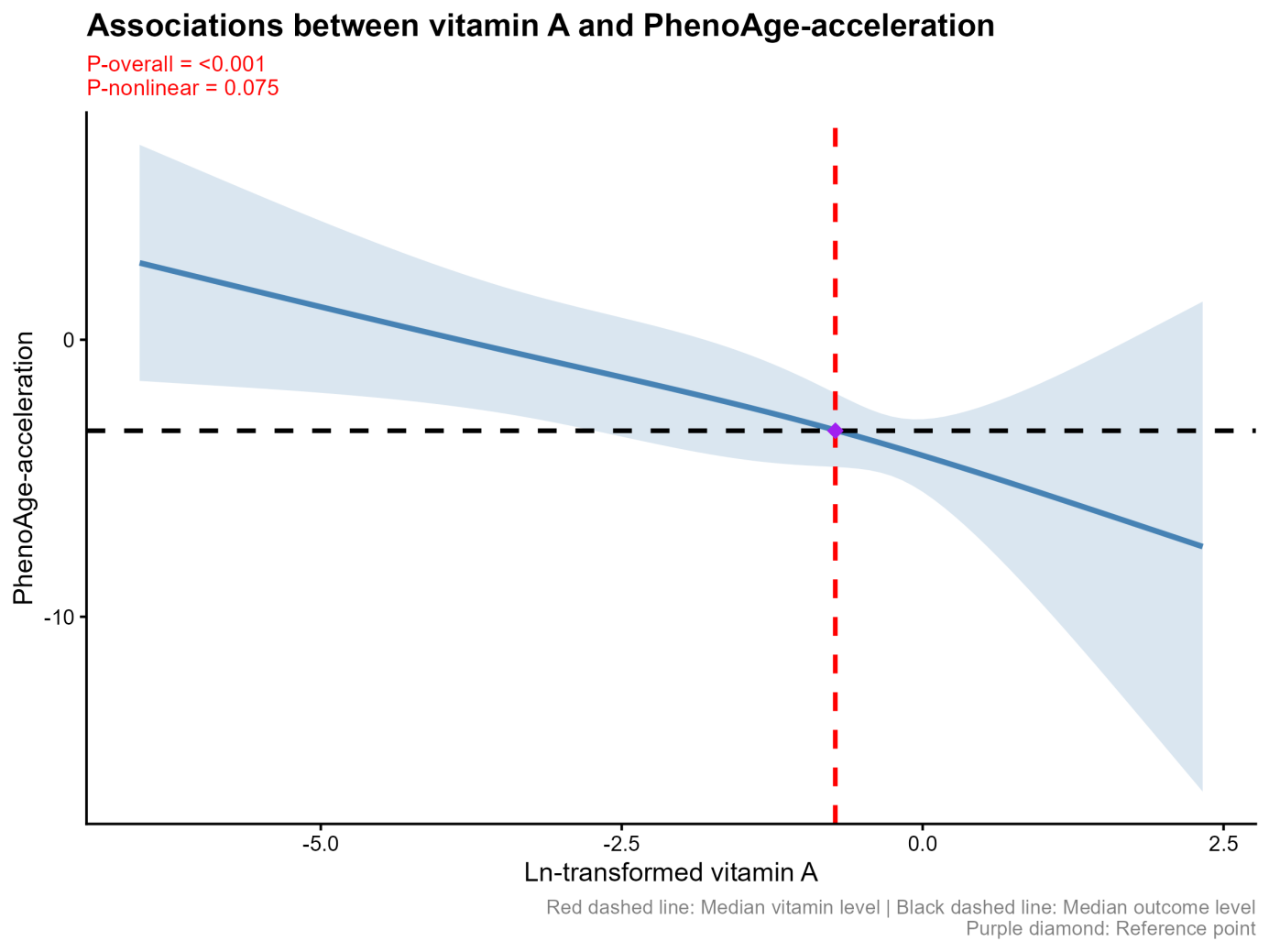
**

**C.HD**

**
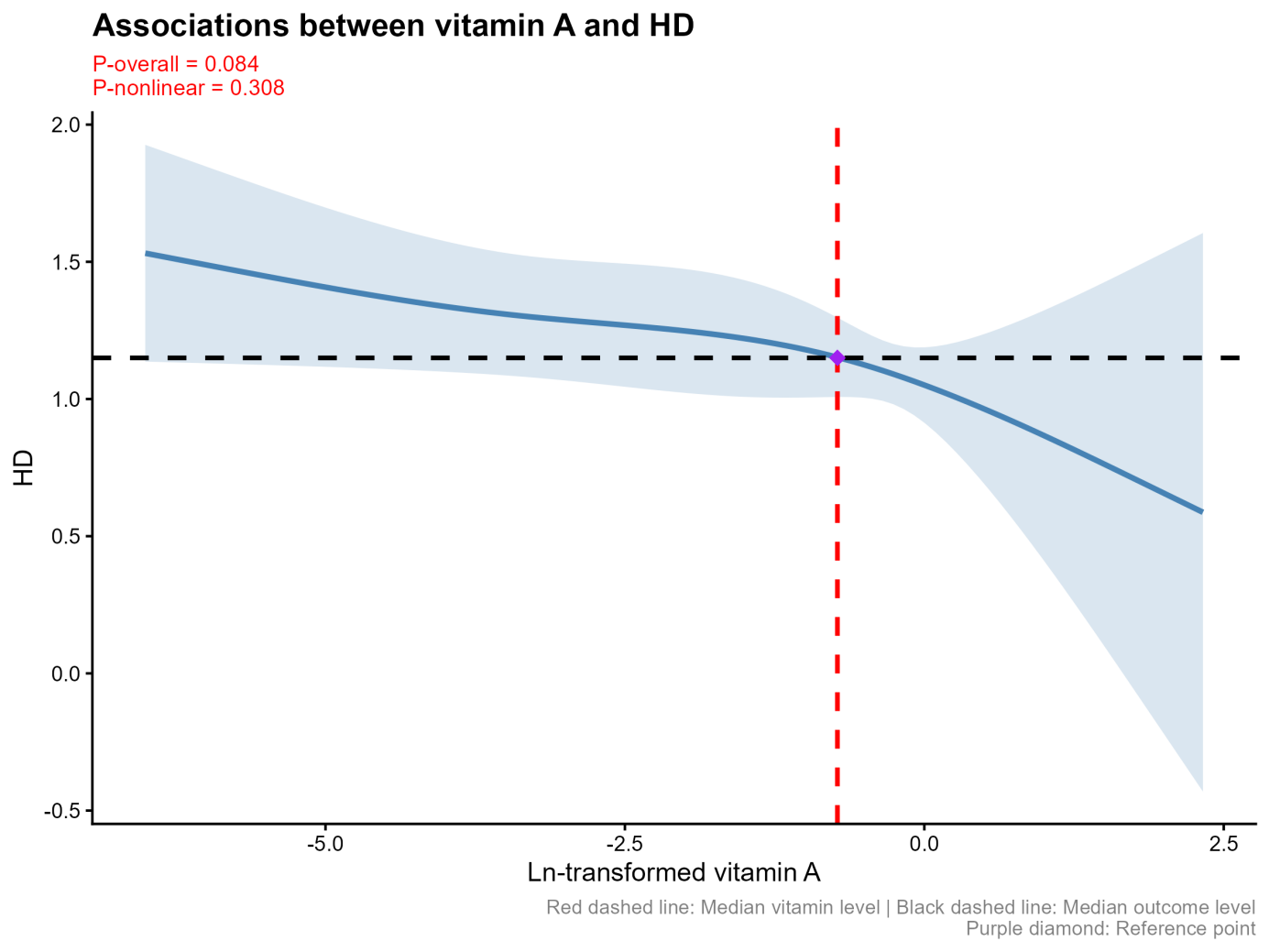
**

Abbreviations: KDM-acceleration, residual-based acceleration of Klemera and Doubal Model biological age (KDM-BA) relative to chronological age; PhenoAge-acceleration, residual-based acceleration of PhenoAge relative to chronological age; HD, homeostatic dysregulation.

Model adjusted for age, sex, race, educational level, marital status, poverty-income ratio, body mass index, smoking status, alcohol consumption, physical activity level, daily energy intake, supplement use, and comorbidity.

Red dashed line: Median vitamin level. Black dashed line: Median outcome level. Purple diamond: Reference point.

**Figure S5. Associations between ln-transformed vitamin B1 intake and biological aging indicators by restricted cubic spline**

**A.KDM-acceleration**

**
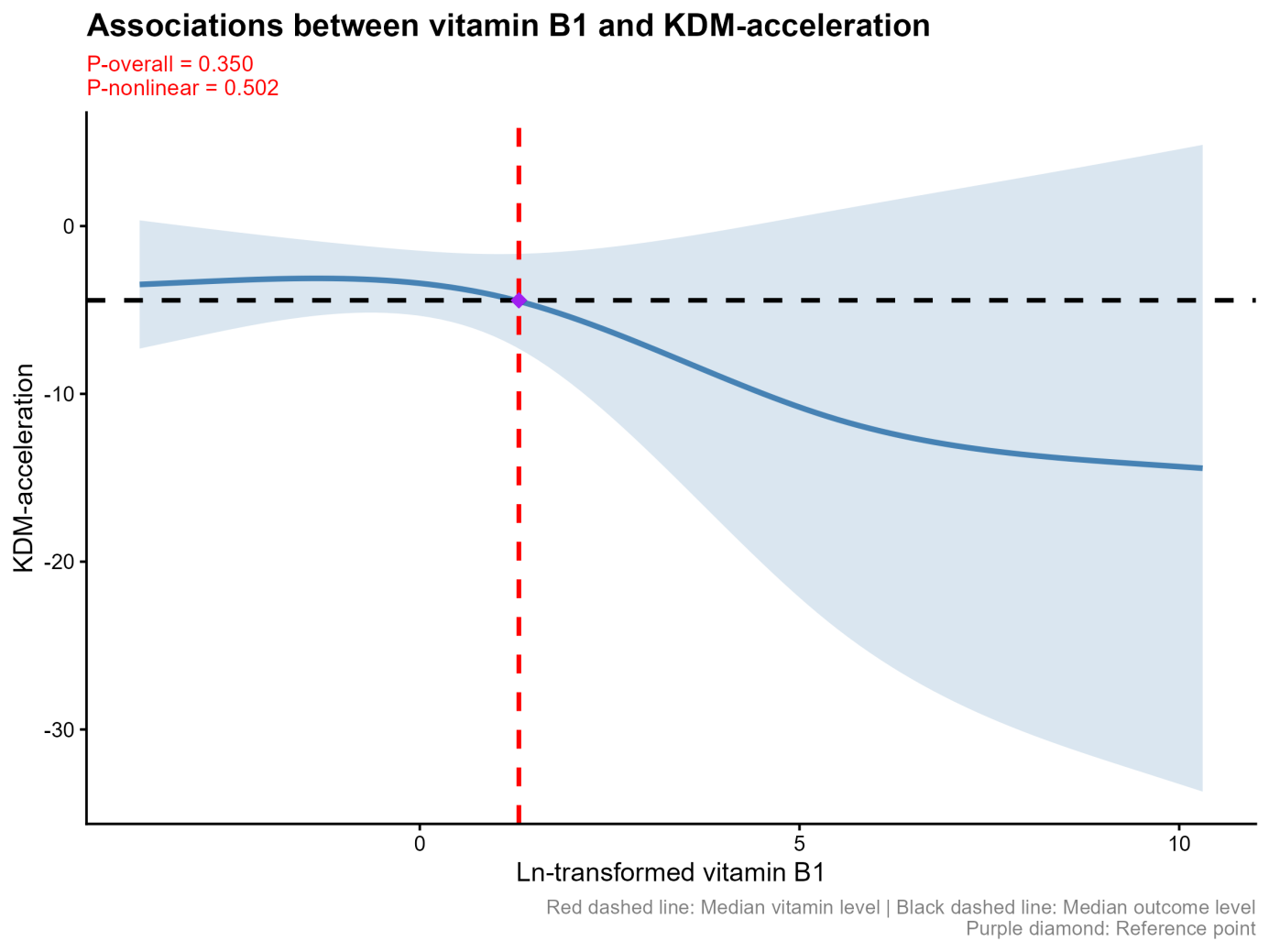
**

**B.PhenoAge-acceleration**

**
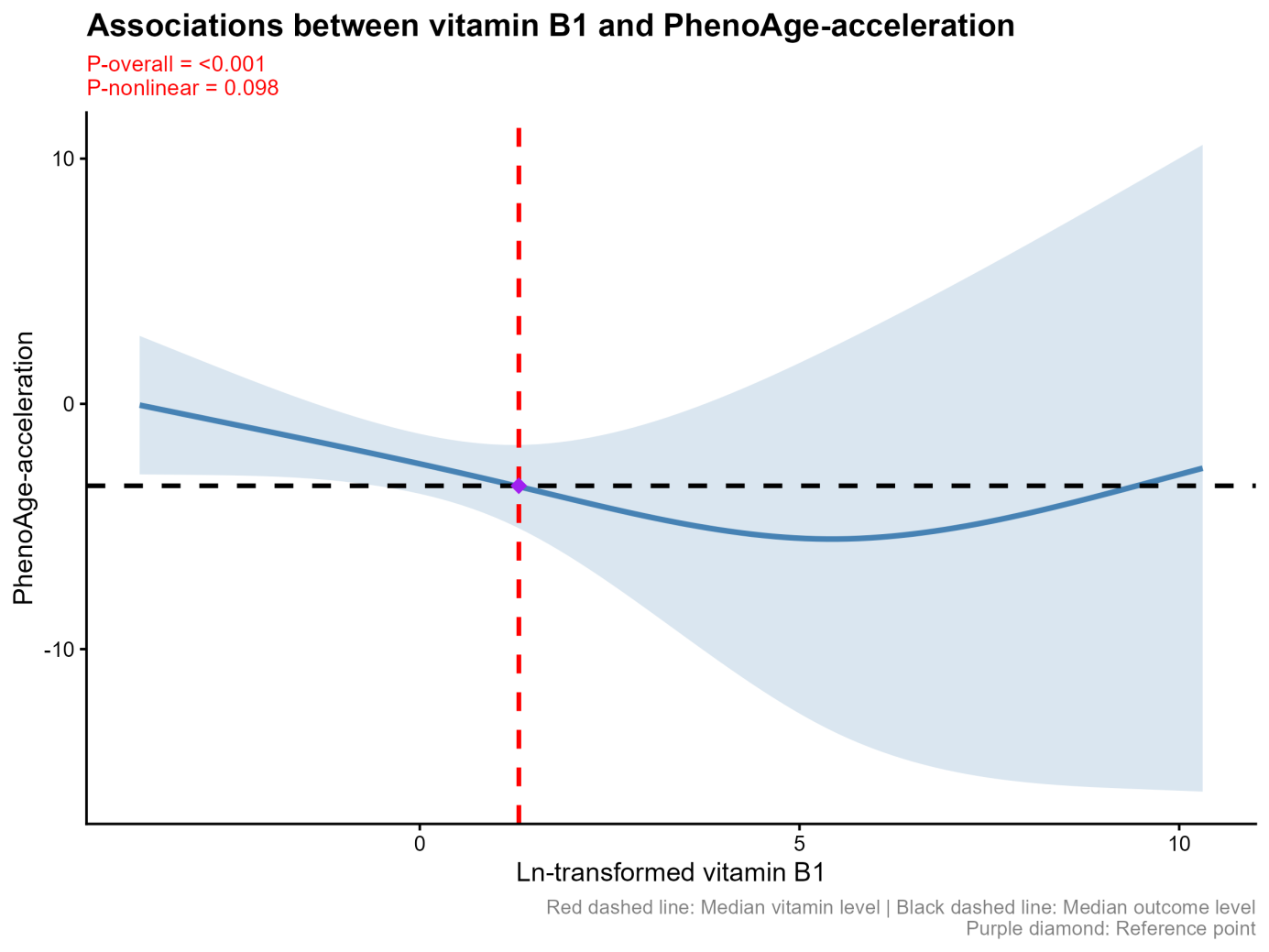
**

**C.HD**

**
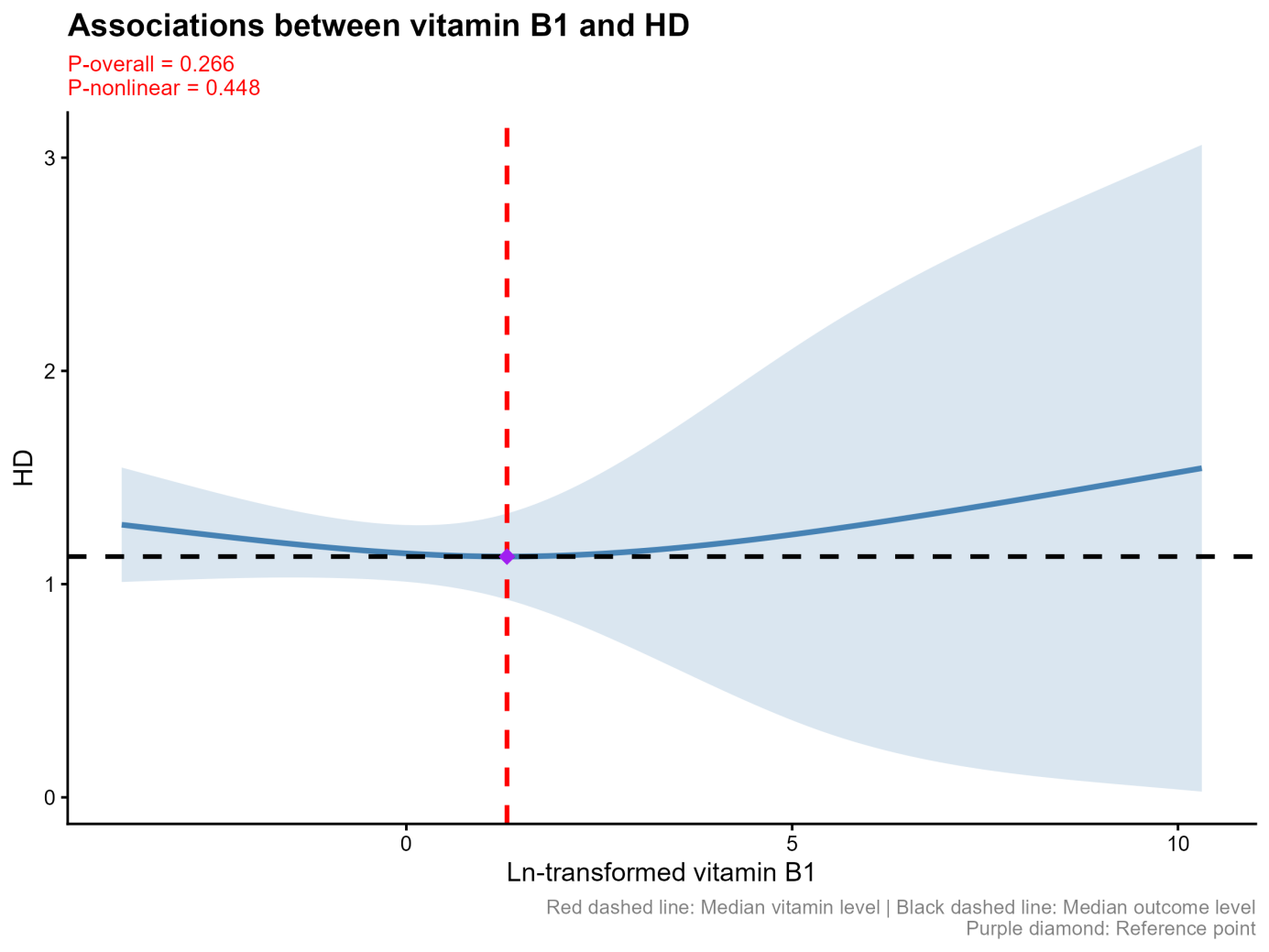
**

Abbreviations: KDM-acceleration, residual-based acceleration of Klemera and Doubal Model biological age (KDM-BA) relative to chronological age; PhenoAge-acceleration, residual-based acceleration of PhenoAge relative to chronological age; HD, homeostatic dysregulation.

Model adjusted for age, sex, race, educational level, marital status, poverty-income ratio, body mass index, smoking status, alcohol consumption, physical activity level, daily energy intake, supplement use, and comorbidity.

Red dashed line: Median vitamin level. Black dashed line: Median outcome level. Purple diamond: Reference point.

**Figure S6. Associations between ln-transformed vitamin B2 intake and biological aging indicators by restricted cubic spline**

**A.KDM-acceleration**

**
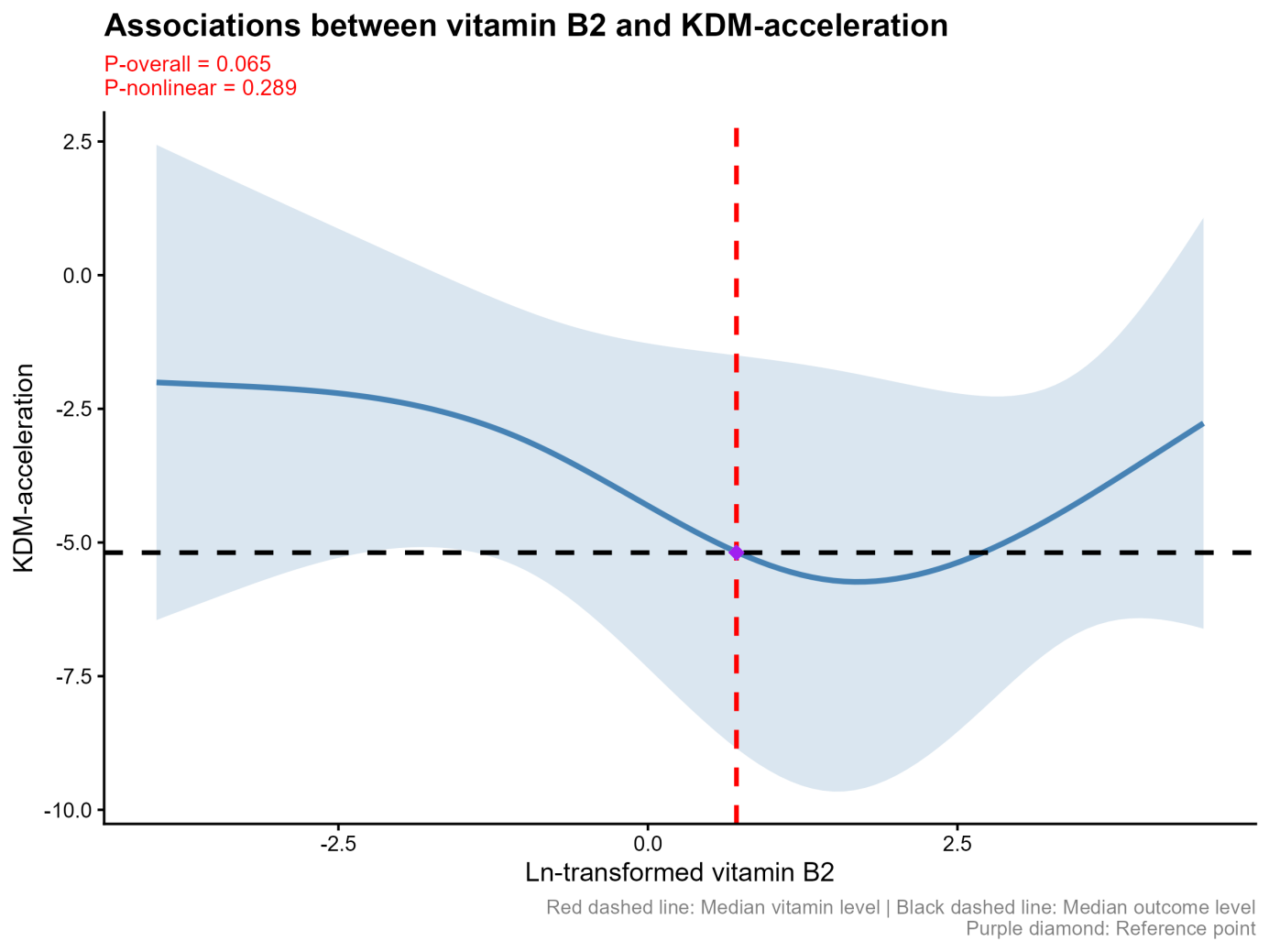
**

**B.PhenoAge-acceleration**

**
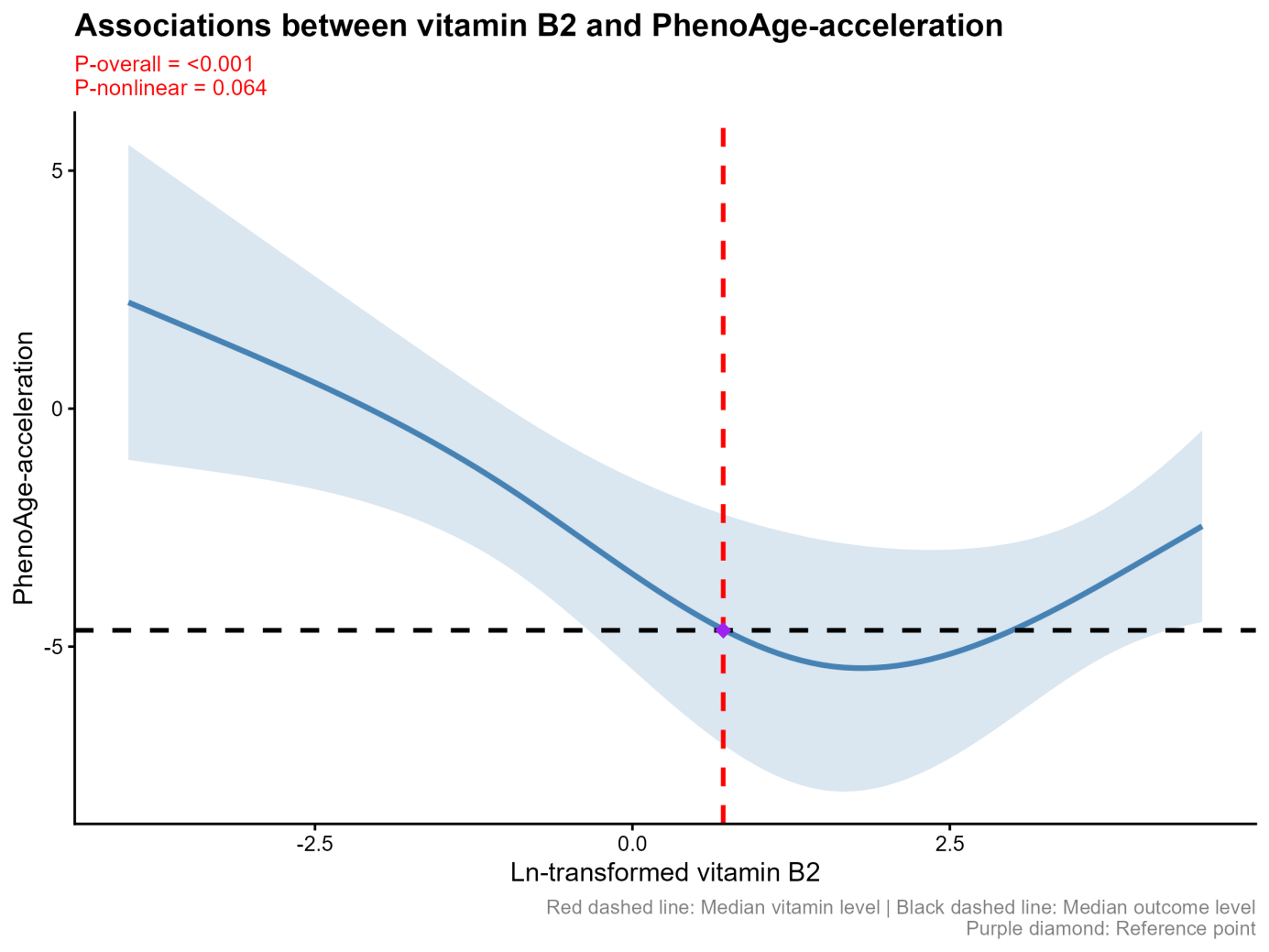
**

**C.HD**

**
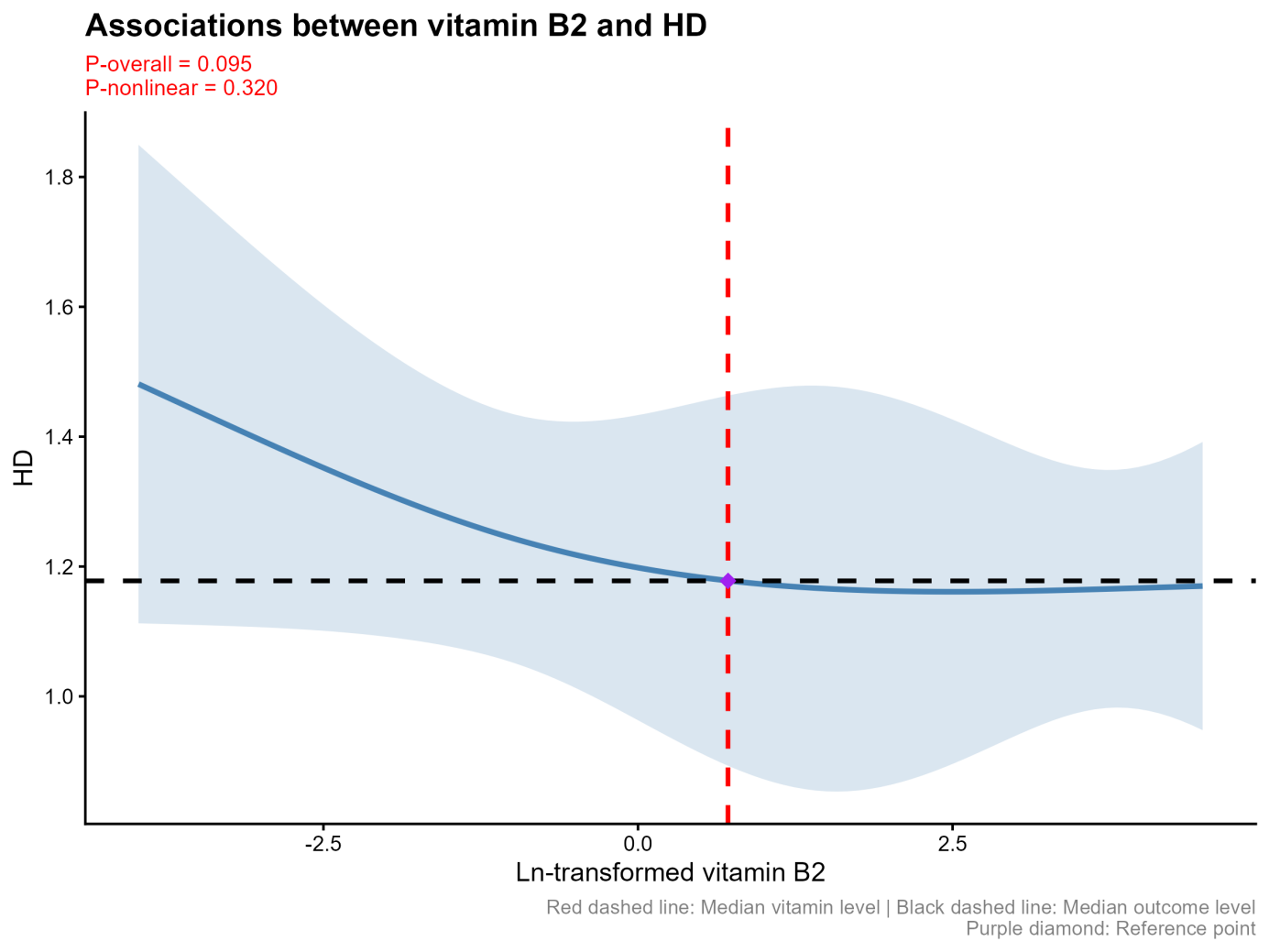
**

Abbreviations: KDM-acceleration, residual-based acceleration of Klemera and Doubal Model biological age (KDM-BA) relative to chronological age; PhenoAge-acceleration, residual-based acceleration of PhenoAge relative to chronological age; HD, homeostatic dysregulation.

Model adjusted for age, sex, race, educational level, marital status, poverty-income ratio, body mass index, smoking status, alcohol consumption, physical activity level, daily energy intake, supplement use, and comorbidity.

Red dashed line: Median vitamin level. Black dashed line: Median outcome level. Purple diamond: Reference point.

**Figure S7. Associations between ln-transformed vitamin B3 intake and biological aging indicators by restricted cubic spline**

**A.KDM-acceleration**

**
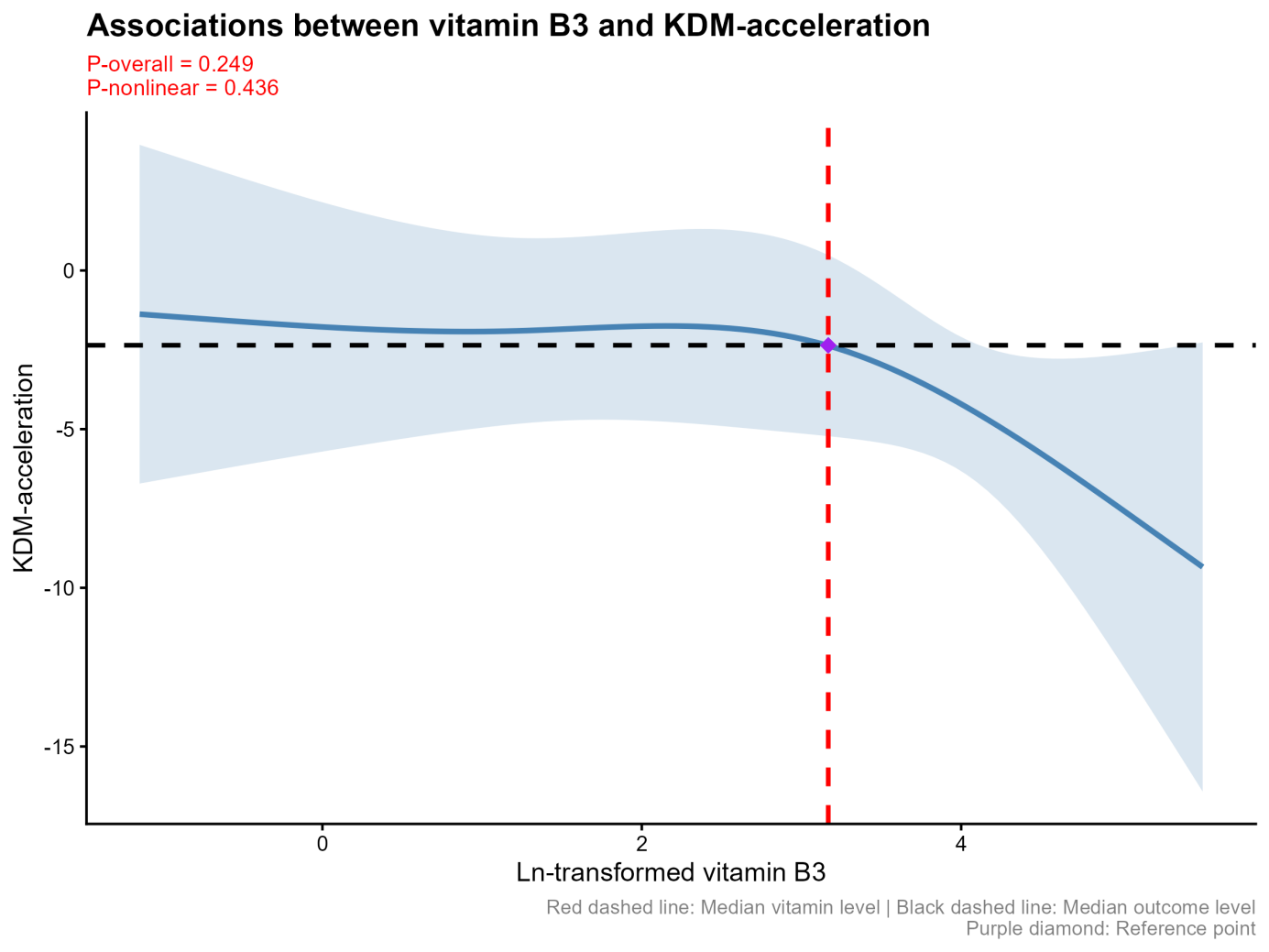
**

**B.PhenoAge-acceleration**

**
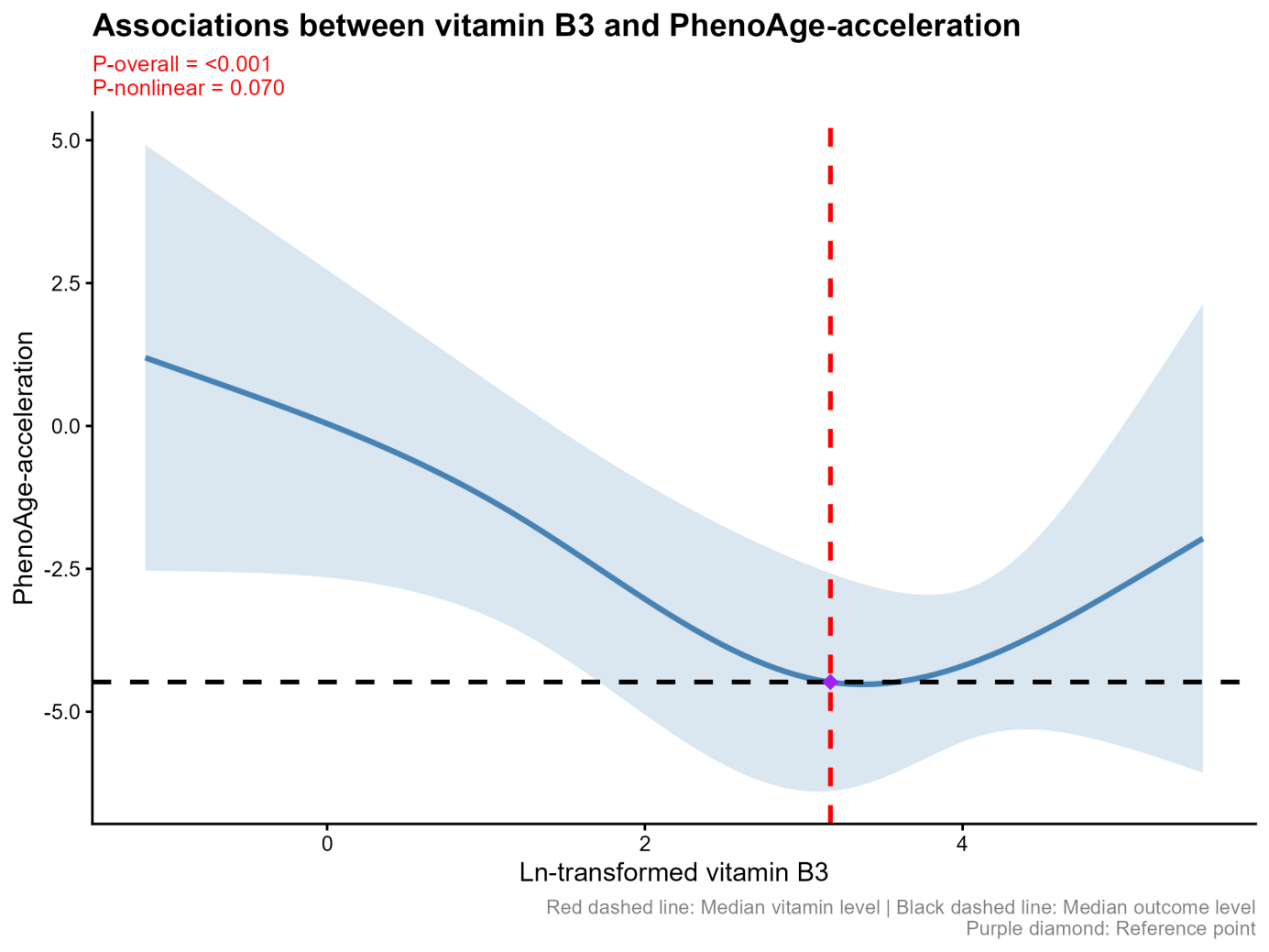
**

**C.HD**

**
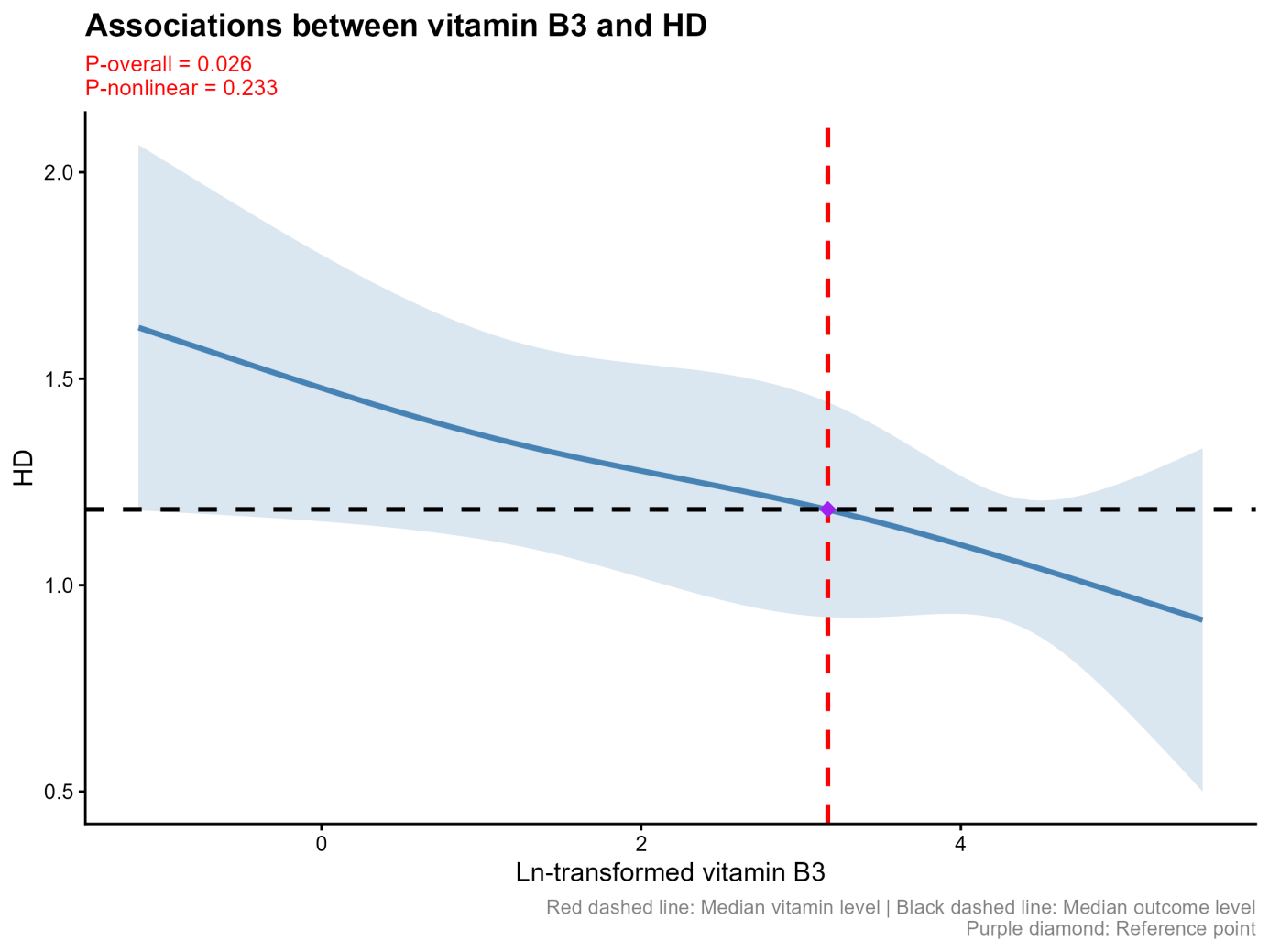
**

Abbreviations: KDM-acceleration, residual-based acceleration of Klemera and Doubal Model biological age (KDM-BA) relative to chronological age; PhenoAge-acceleration, residual-based acceleration of PhenoAge relative to chronological age; HD, homeostatic dysregulation.

Model adjusted for age, sex, race, educational level, marital status, poverty-income ratio, body mass index, smoking status, alcohol consumption, physical activity level, daily energy intake, supplement use, and comorbidity.

Red dashed line: Median vitamin level. Black dashed line: Median outcome level. Purple diamond: Reference point.

**Figure S8. Associations between ln-transformed vitamin B6 intake and biological aging indicators by restricted cubic spline**

**A.KDM-acceleration**

**
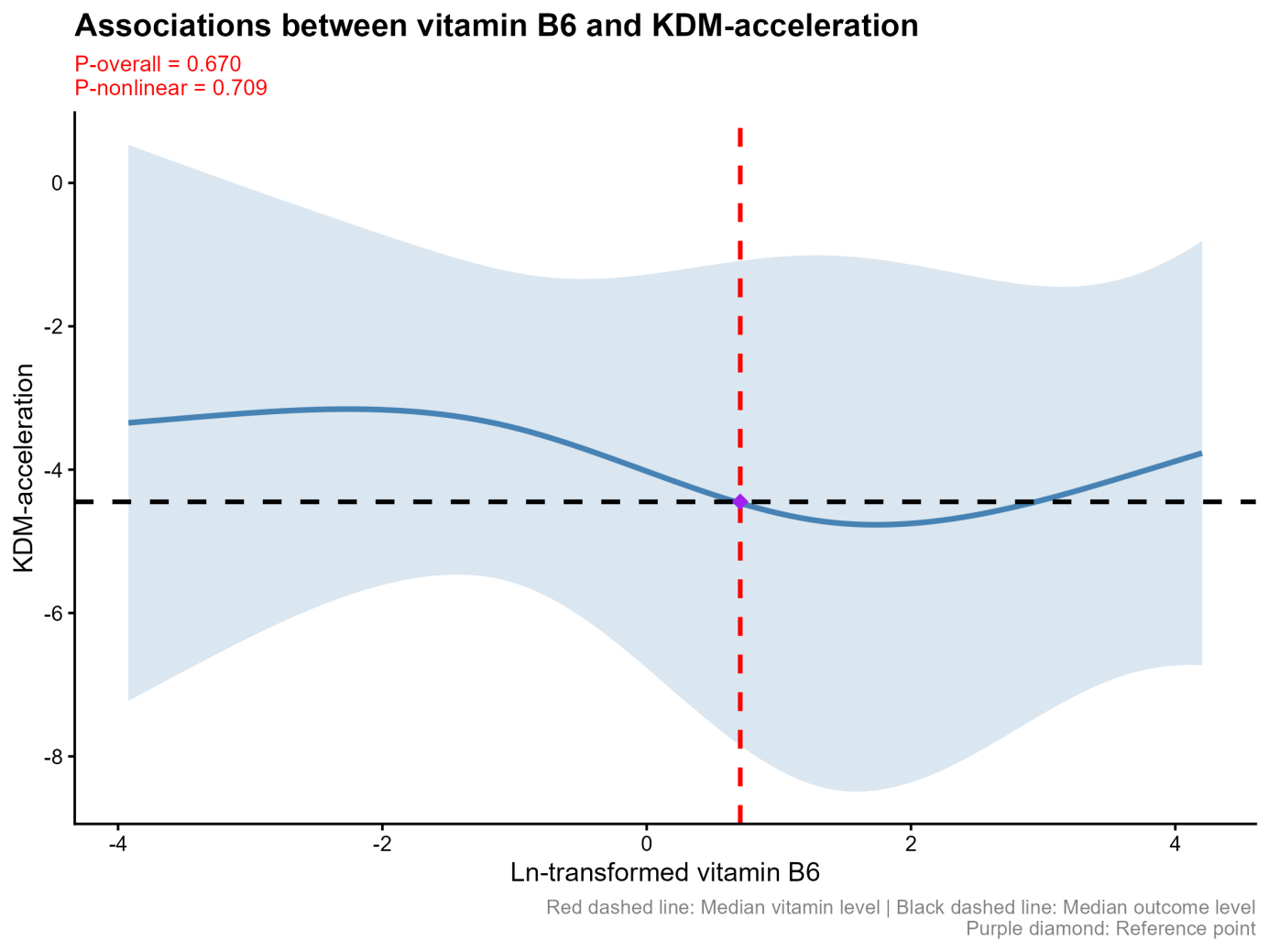
**

**B.PhenoAge-acceleration**

**
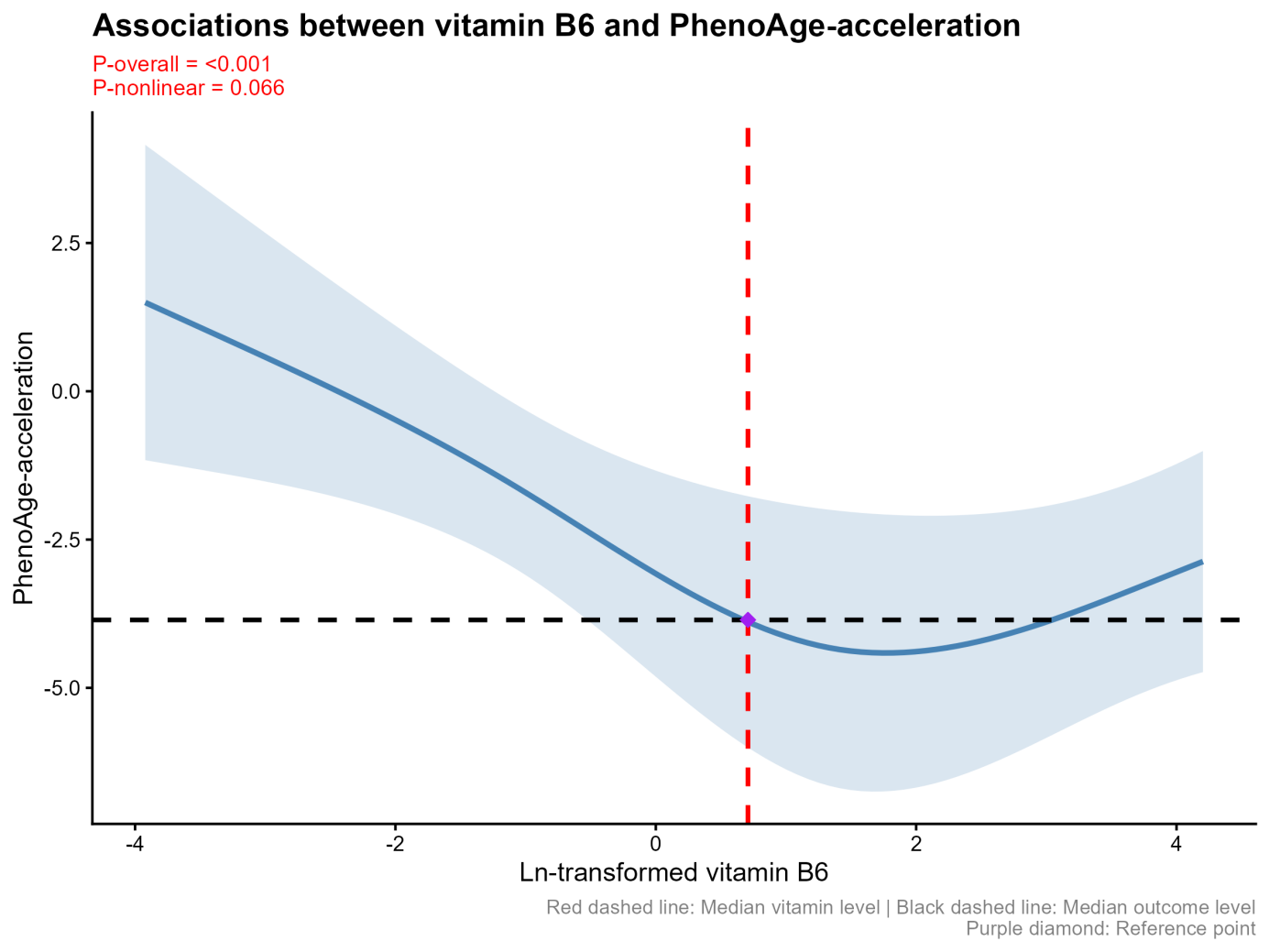
**

**C.HD**

**
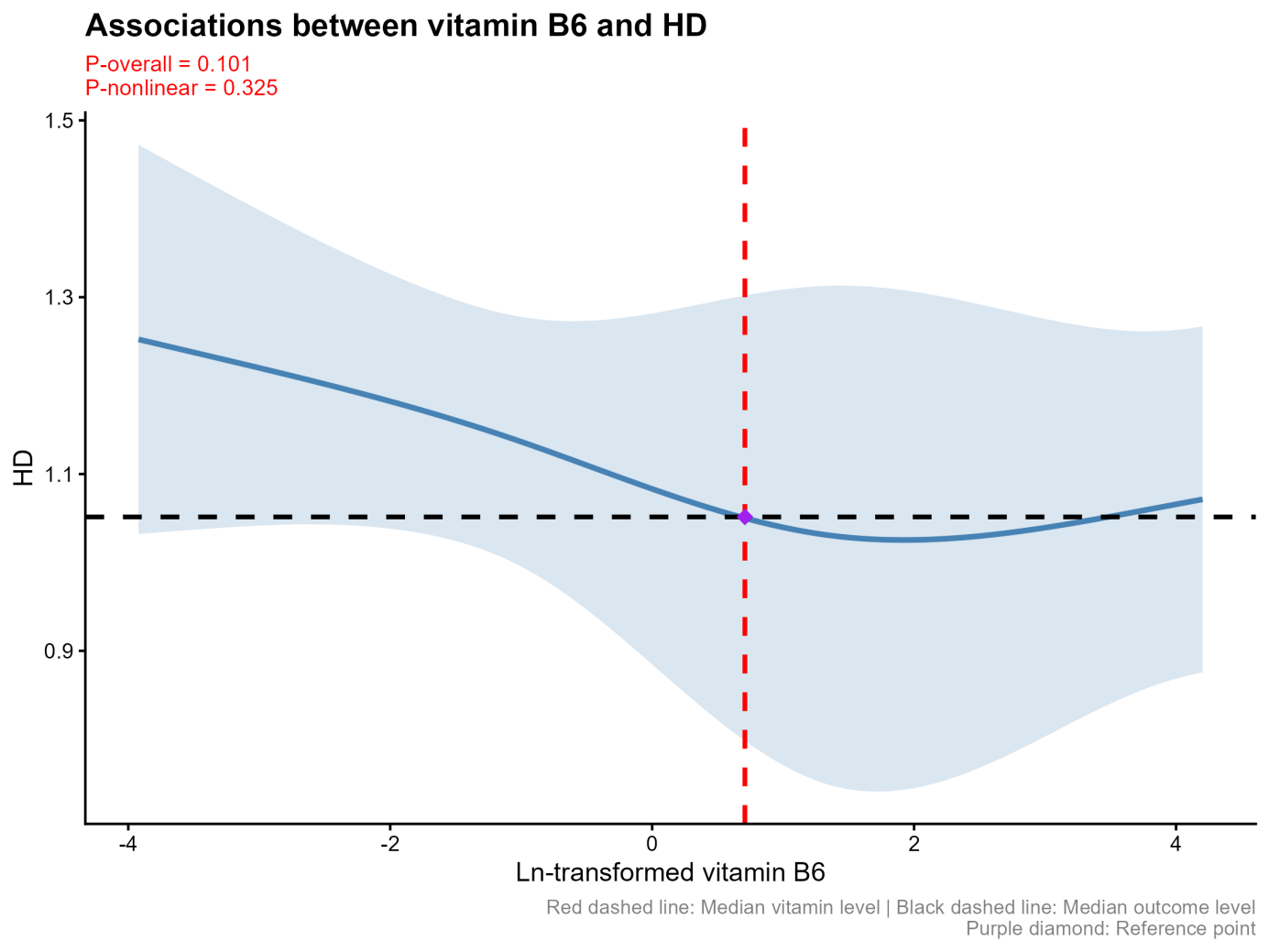
**

Abbreviations: KDM-acceleration, residual-based acceleration of Klemera and Doubal Model biological age (KDM-BA) relative to chronological age; PhenoAge-acceleration, residual-based acceleration of PhenoAge relative to chronological age; HD, homeostatic dysregulation.

Model adjusted for age, sex, race, educational level, marital status, poverty-income ratio, body mass index, smoking status, alcohol consumption, physical activity level, daily energy intake, supplement use, and comorbidity.

Red dashed line: Median vitamin level. Black dashed line: Median outcome level. Purple diamond: Reference point.

**Figure S9. Associations between ln-transformed vitamin B9 intake and biological aging indicators by restricted cubic spline**

**A.KDM-acceleration**

**
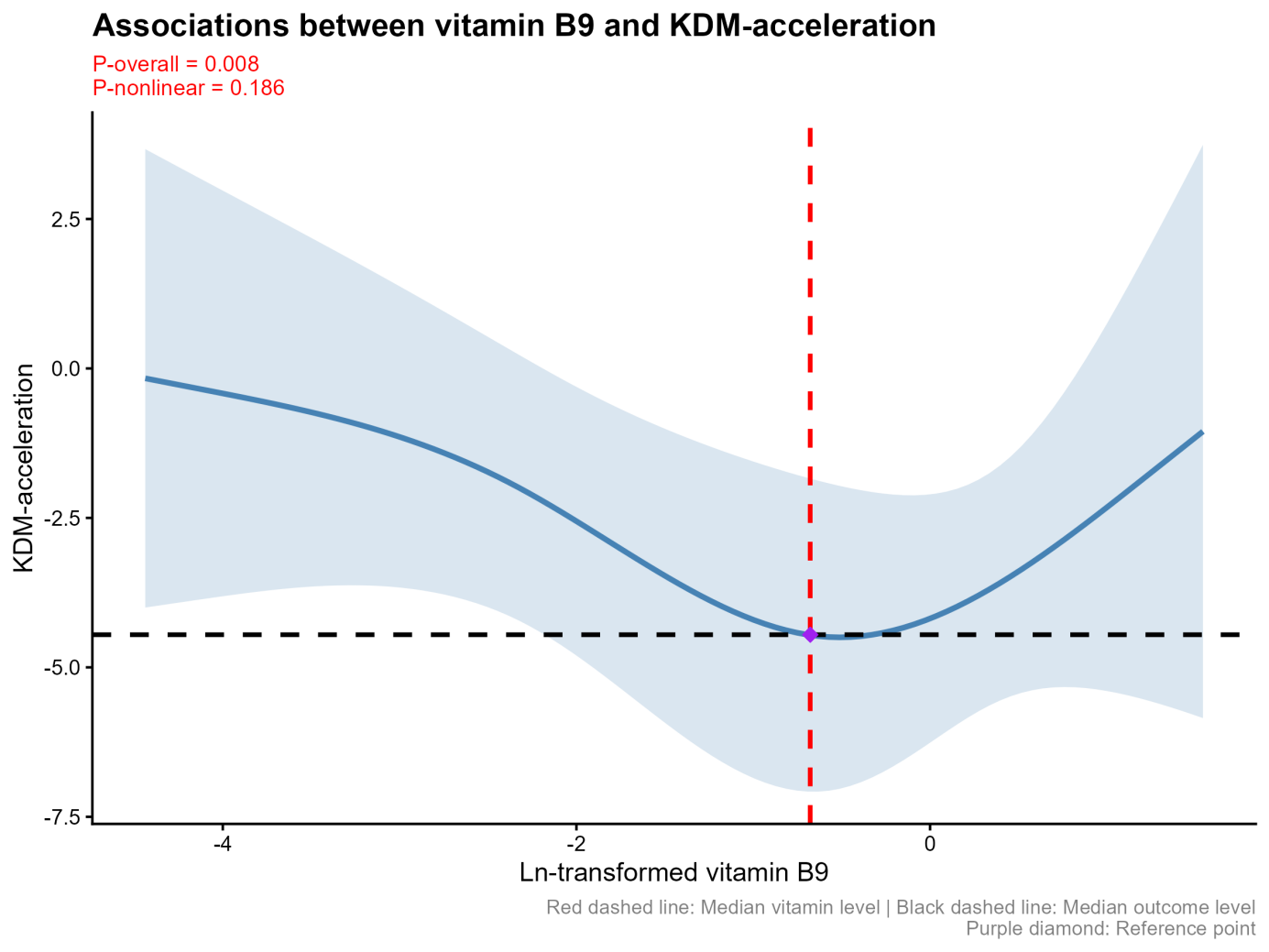
**

**B.PhenoAge-acceleration**

**
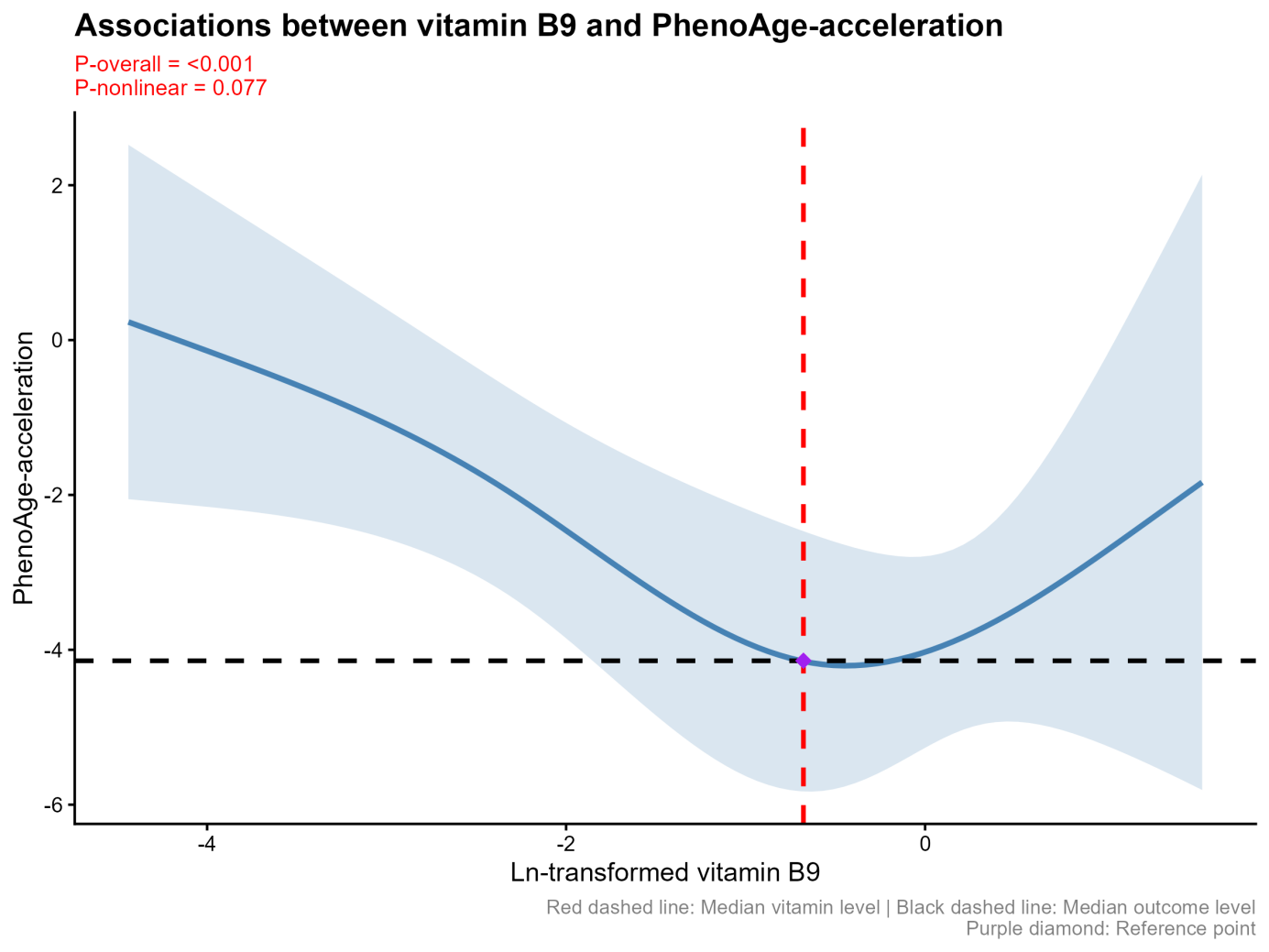
**

**C.HD**

**
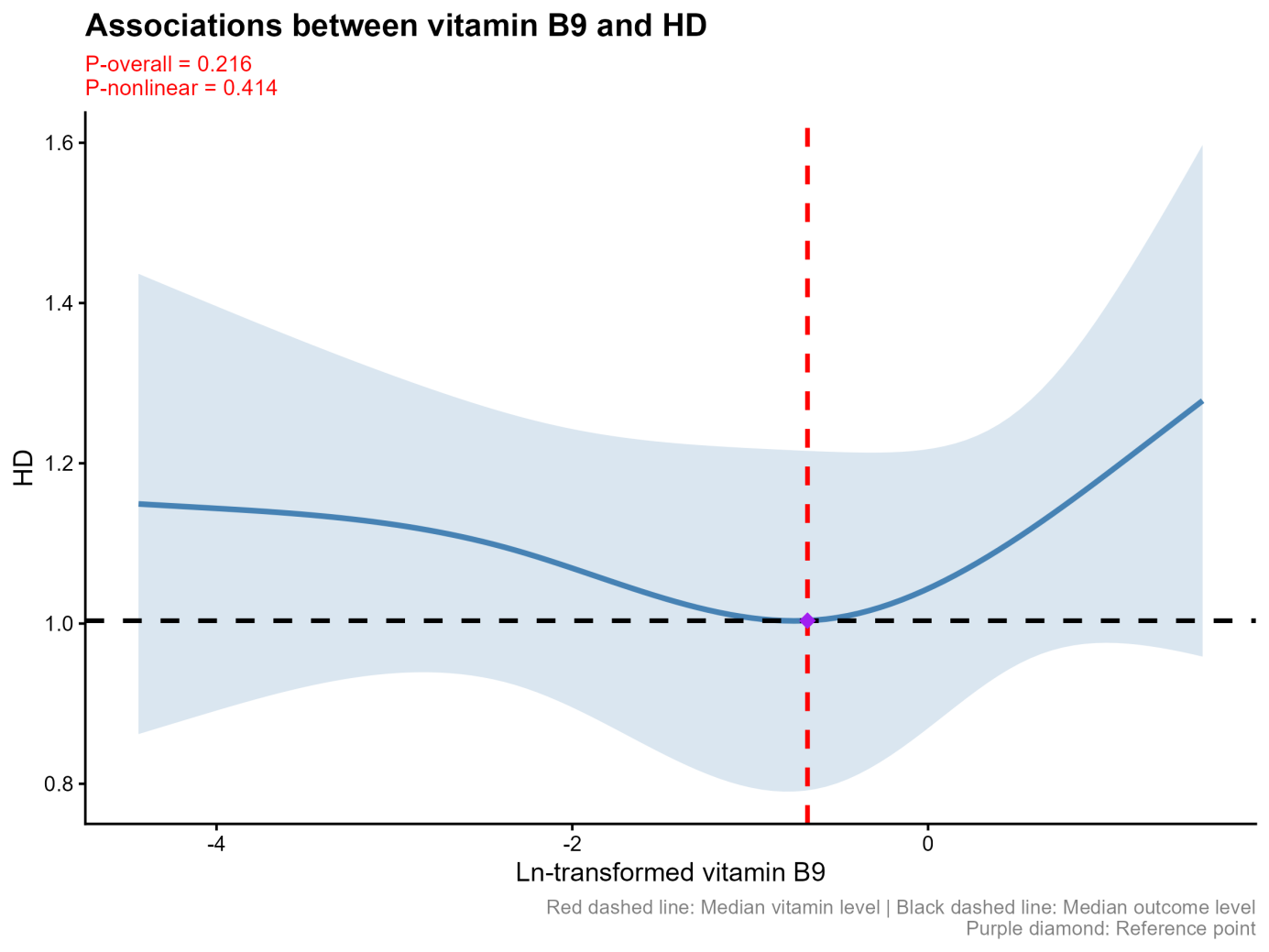
**

Abbreviations: KDM-acceleration, residual-based acceleration of Klemera and Doubal Model biological age (KDM-BA) relative to chronological age; PhenoAge-acceleration, residual-based acceleration of PhenoAge relative to chronological age; HD, homeostatic dysregulation.

Model adjusted for age, sex, race, educational level, marital status, poverty-income ratio, body mass index, smoking status, alcohol consumption, physical activity level, daily energy intake, supplement use, and comorbidity.

Red dashed line: Median vitamin level. Black dashed line: Median outcome level. Purple diamond: Reference point.

**Figure S10. Associations between ln-transformed vitamin B12 intake and biological aging indicators by restricted cubic spline**

**A.KDM-acceleration**

**
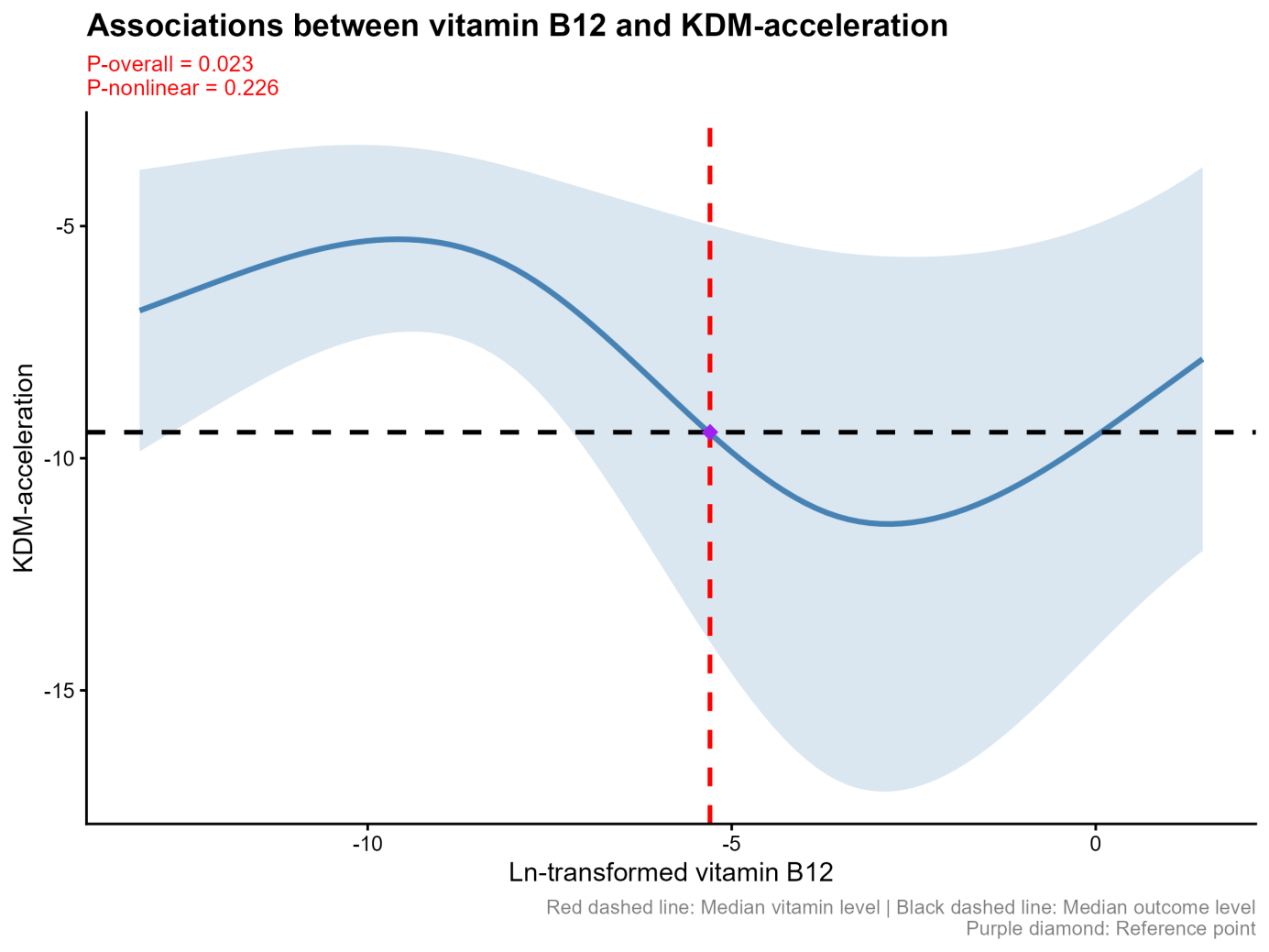
**

**B.PhenoAge-acceleration**

**
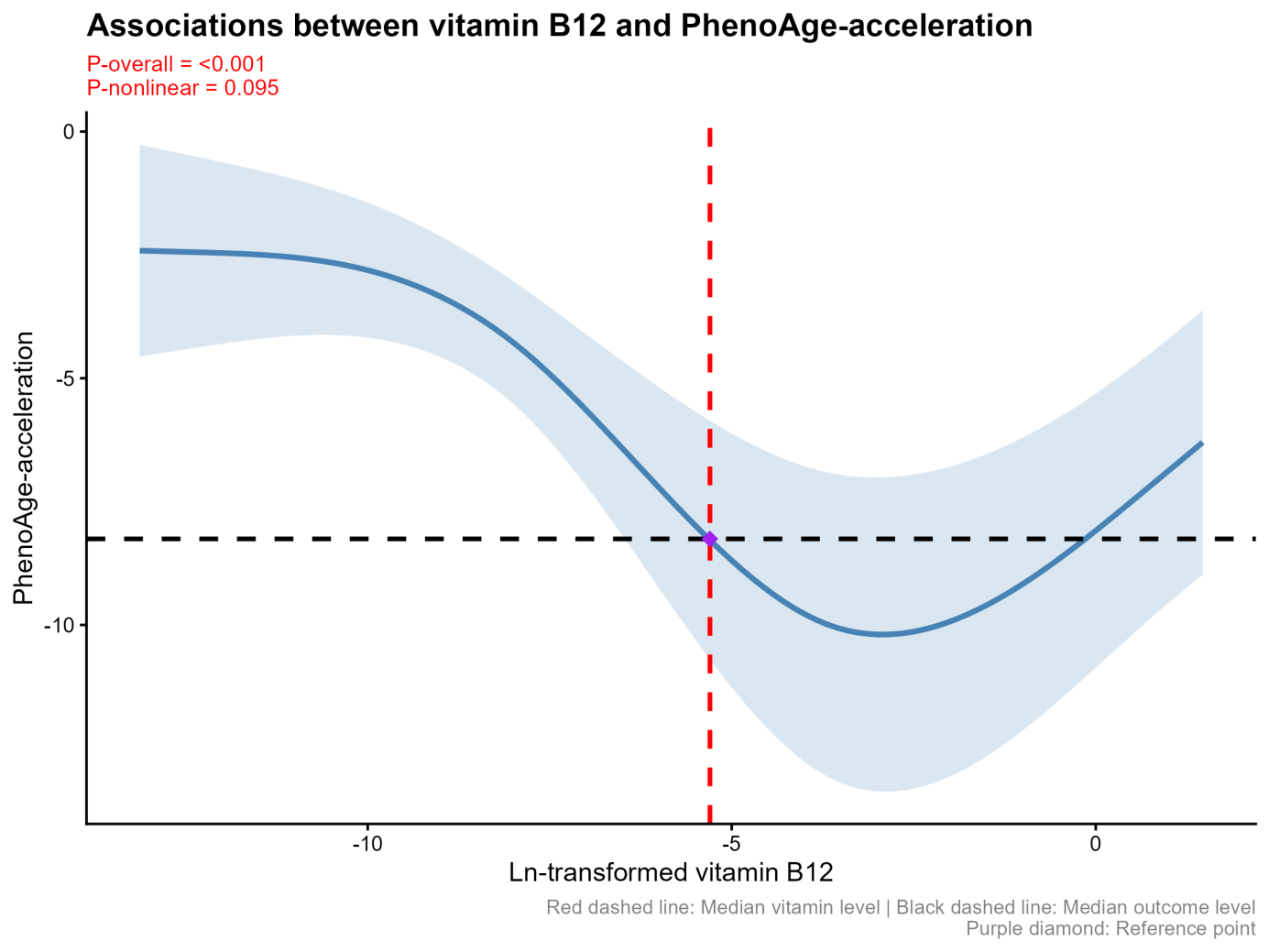
**

**C.HD**

**
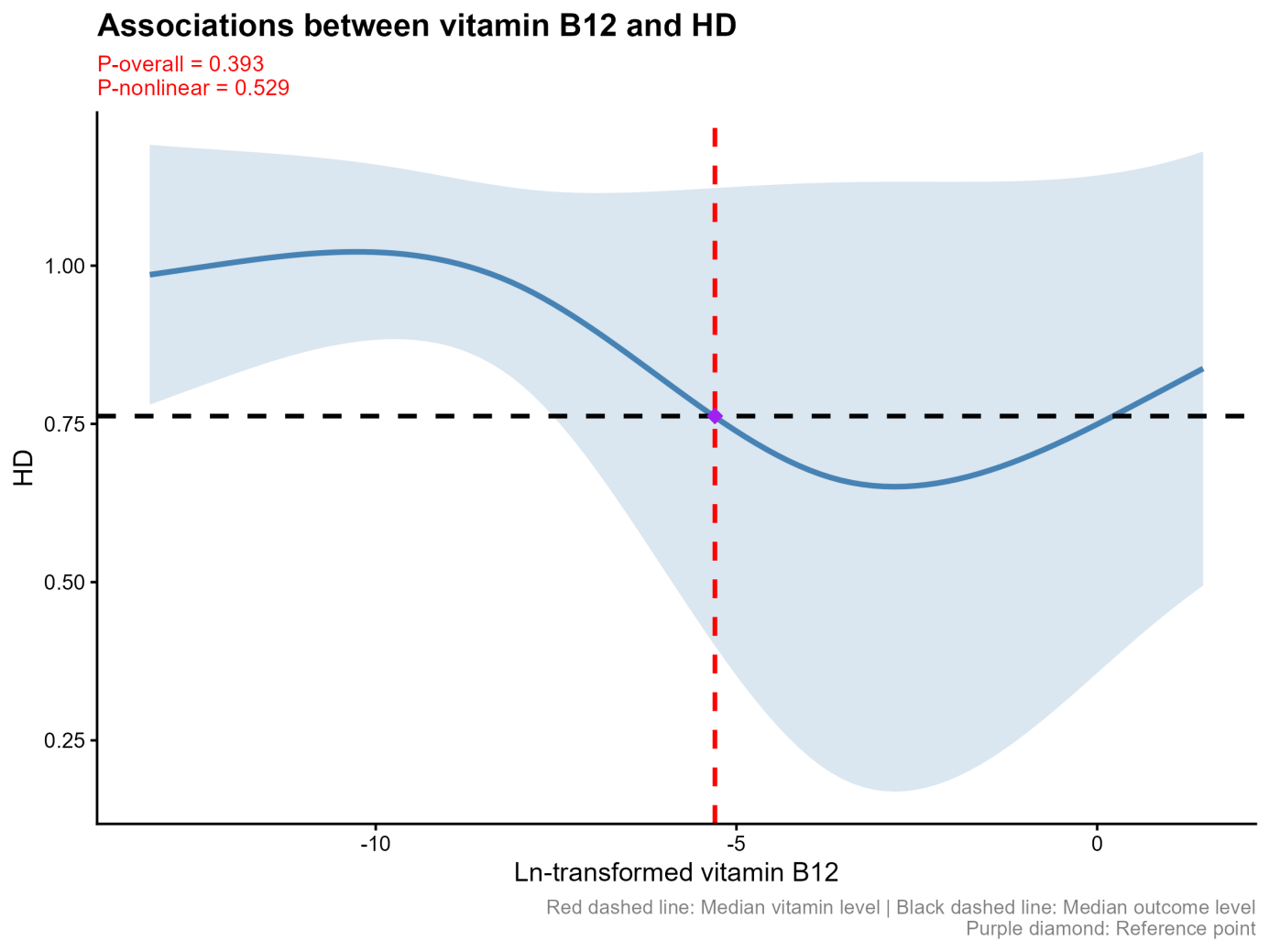
**

Abbreviations: KDM-acceleration, residual-based acceleration of Klemera and Doubal Model biological age (KDM-BA) relative to chronological age; PhenoAge-acceleration, residual-based acceleration of PhenoAge relative to chronological age; HD, homeostatic dysregulation.

Model adjusted for age, sex, race, educational level, marital status, poverty-income ratio, body mass index, smoking status, alcohol consumption, physical activity level, daily energy intake, supplement use, and comorbidity.

Red dashed line: Median vitamin level. Black dashed line: Median outcome level. Purple diamond: Reference point.

**Figure S11. Associations between ln-transformed vitamin C intake and biological aging indicators by restricted cubic spline**

**A.KDM-acceleration**

**
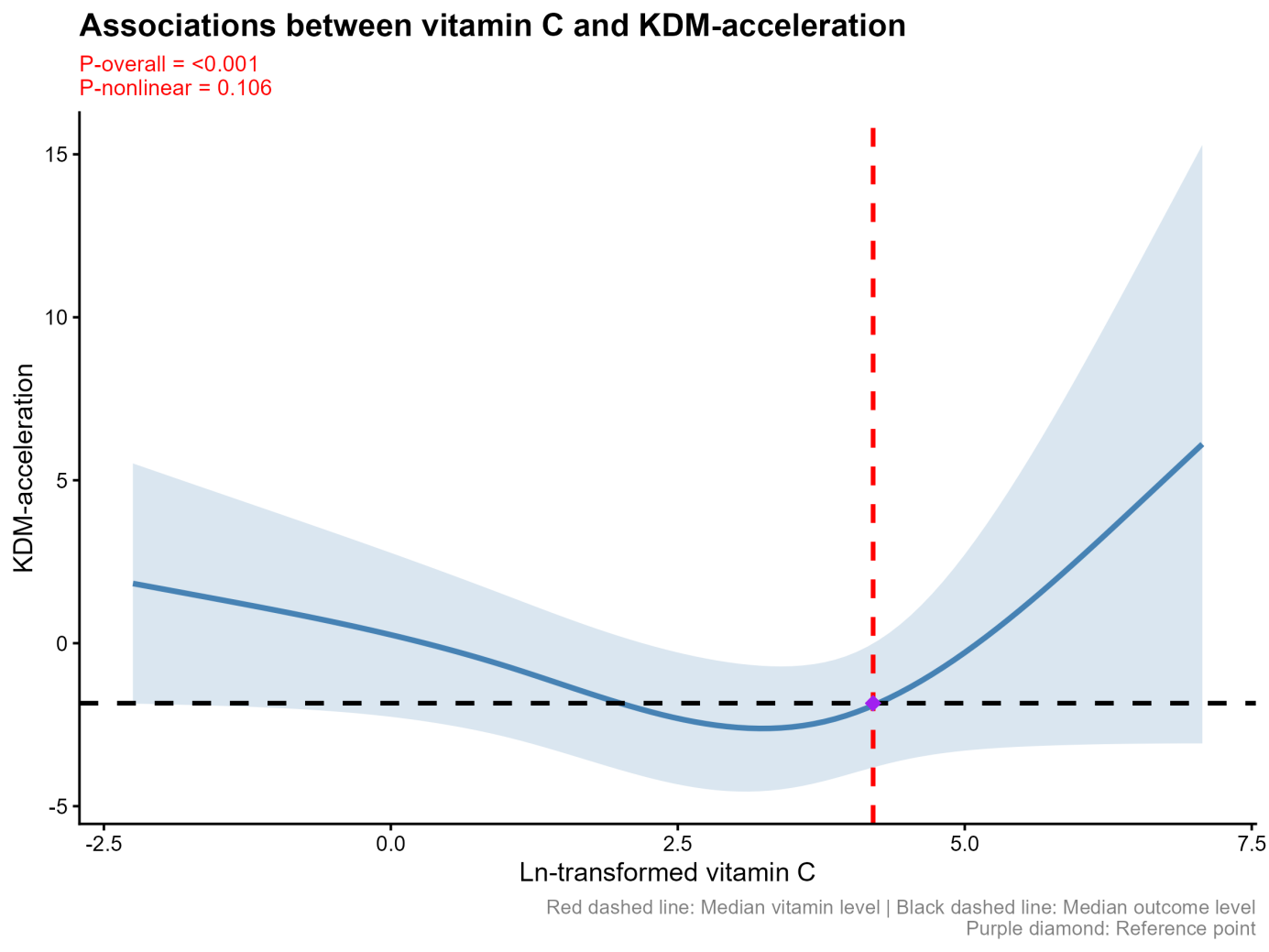
**

**B.PhenoAge-acceleration**

**
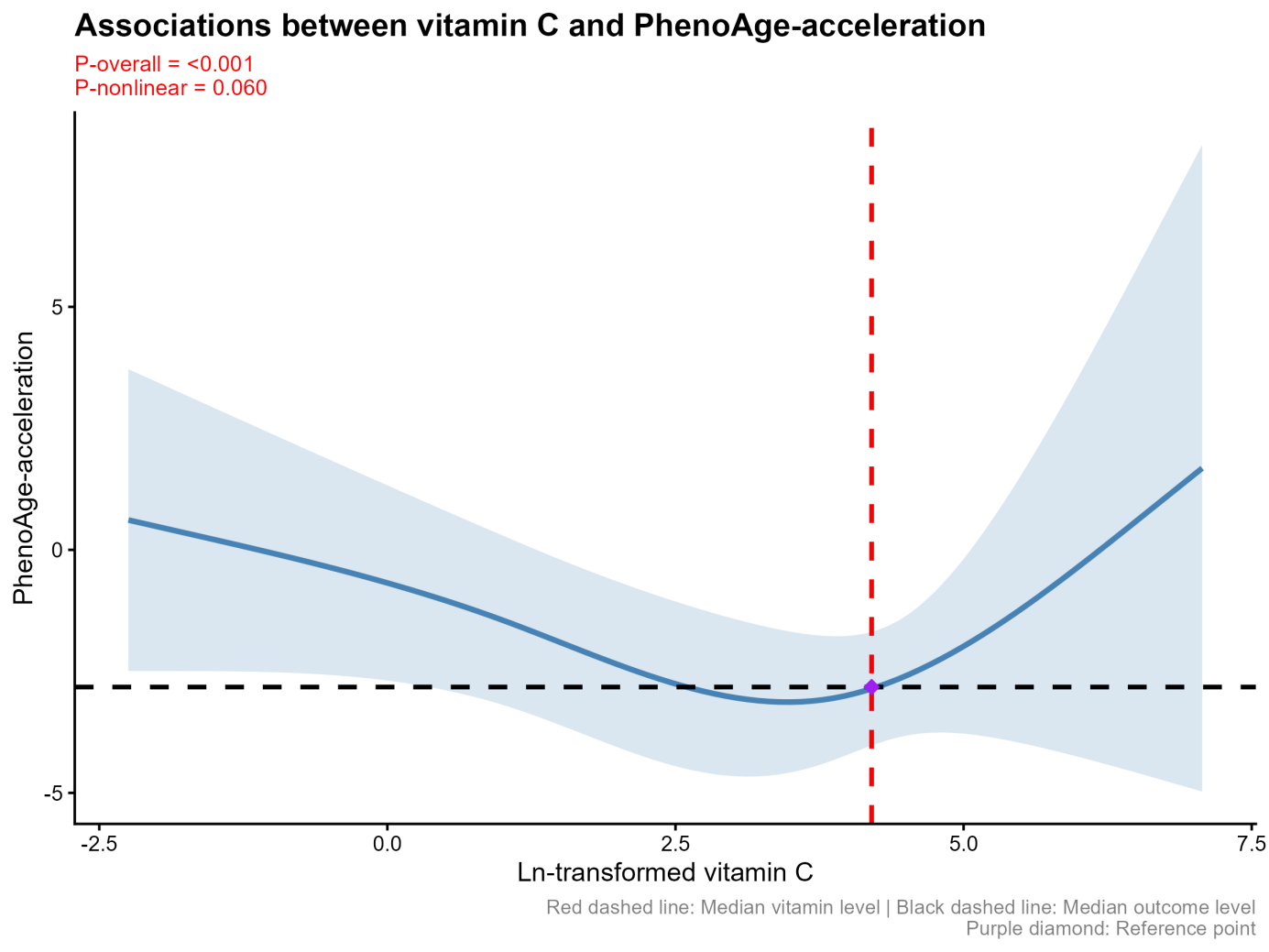
**

**C.HD**

**
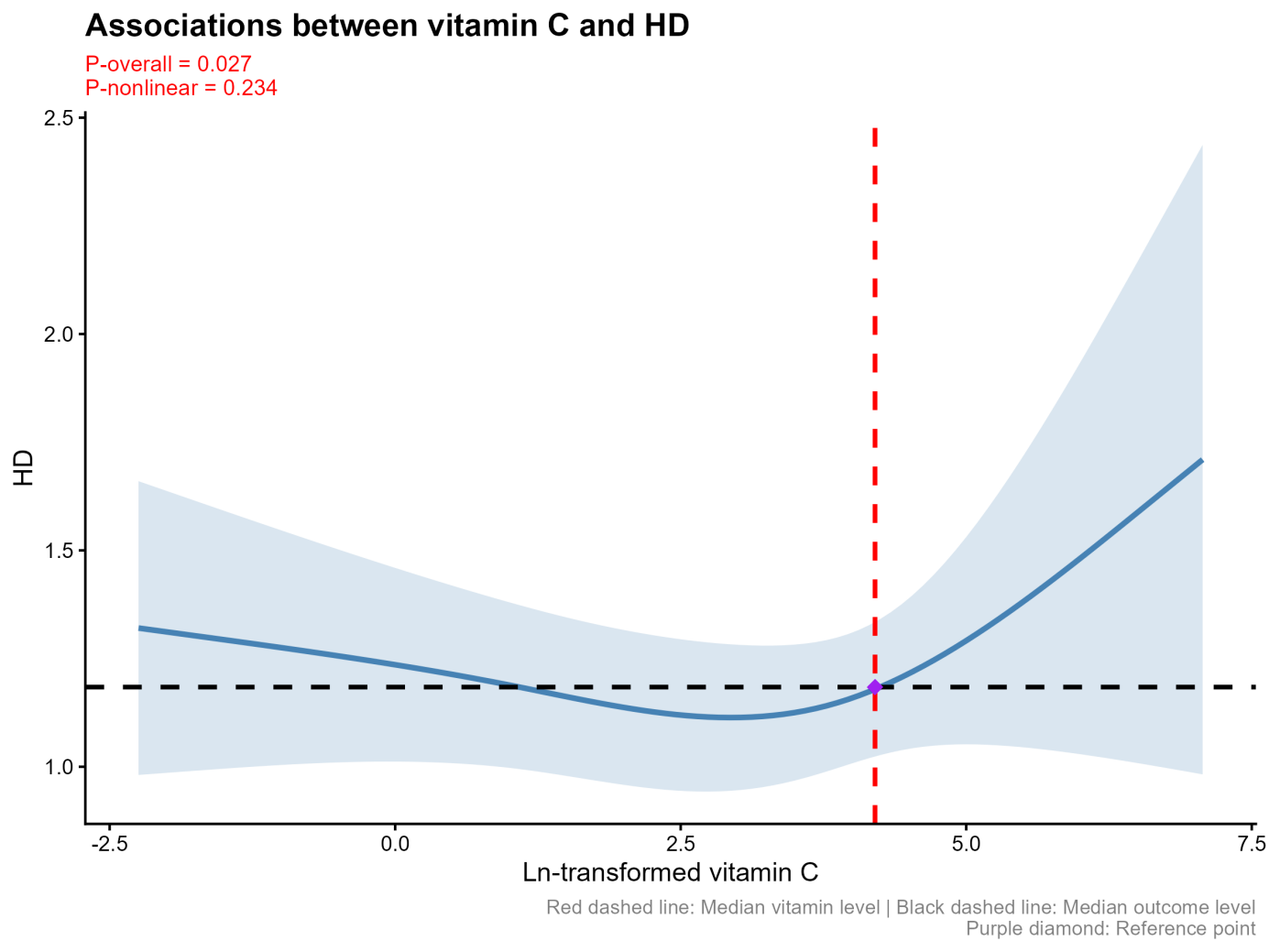
**

Abbreviations: KDM-acceleration, residual-based acceleration of Klemera and Doubal Model biological age (KDM-BA) relative to chronological age; PhenoAge-acceleration, residual-based acceleration of PhenoAge relative to chronological age; HD, homeostatic dysregulation.

Model adjusted for age, sex, race, educational level, marital status, poverty-income ratio, body mass index, smoking status, alcohol consumption, physical activity level, daily energy intake, supplement use, and comorbidity.

Red dashed line: Median vitamin level. Black dashed line: Median outcome level. Purple diamond: Reference point.

**Figure S12. Associations between ln-transformed vitamin D intake and biological aging indicators by restricted cubic spline**

**A.KDM-acceleration**

**
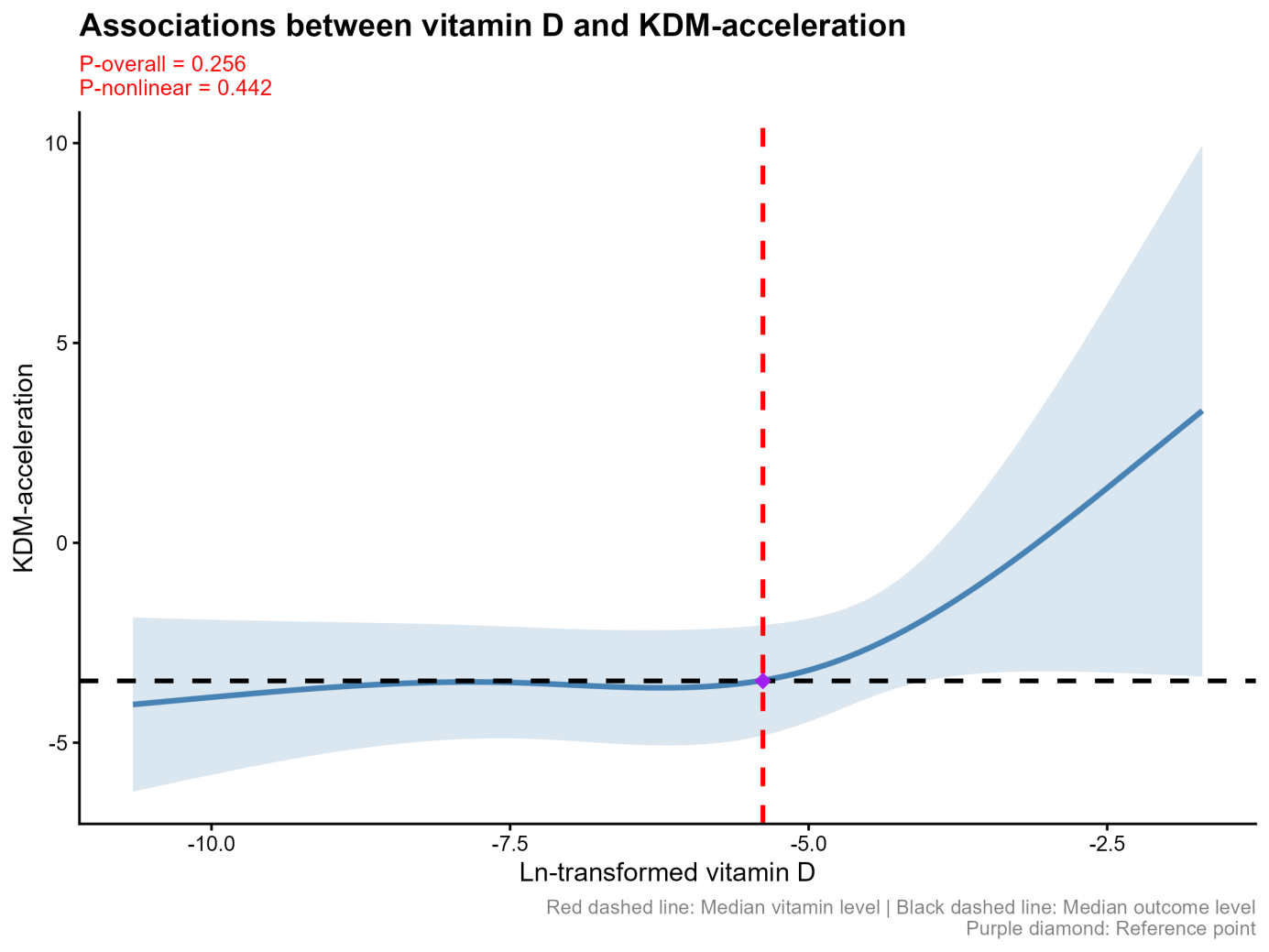
**

**B.PhenoAge-acceleration**

**
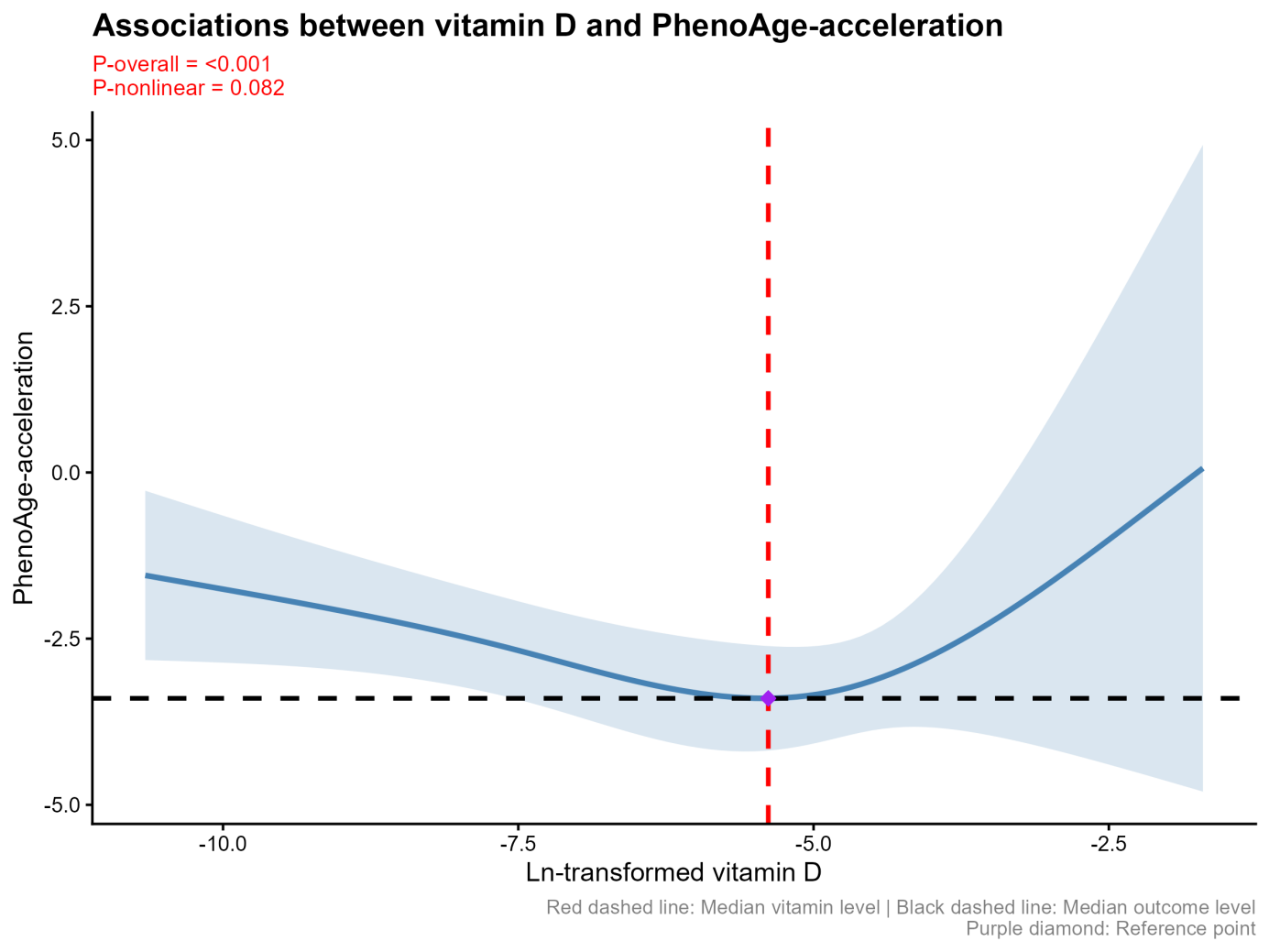
**

**C.HD**

**
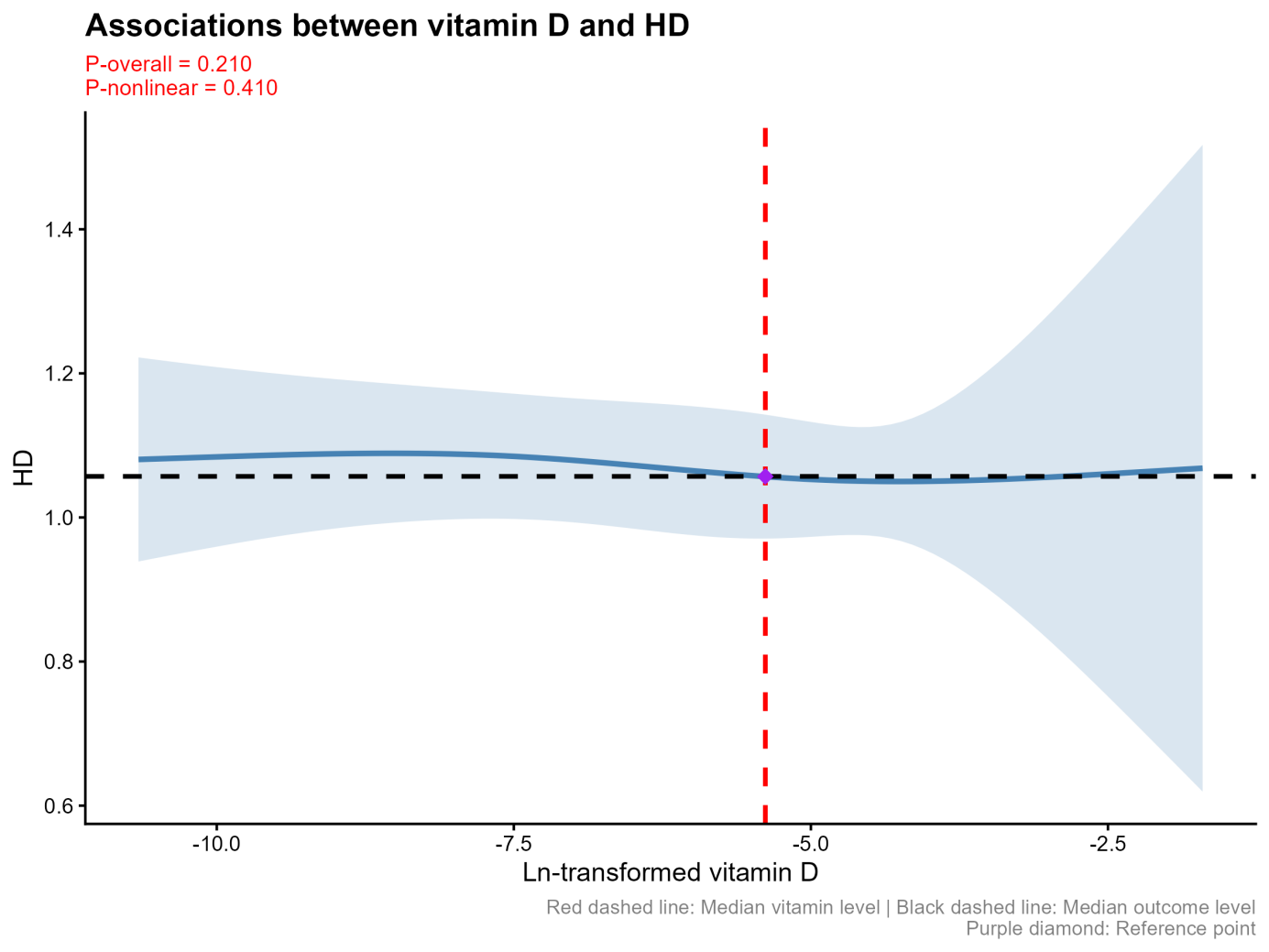
**

Abbreviations: KDM-acceleration, residual-based acceleration of Klemera and Doubal Model biological age (KDM-BA) relative to chronological age; PhenoAge-acceleration, residual-based acceleration of PhenoAge relative to chronological age; HD, homeostatic dysregulation.

Model adjusted for age, sex, race, educational level, marital status, poverty-income ratio, body mass index, smoking status, alcohol consumption, physical activity level, daily energy intake, supplement use, and comorbidity.

Red dashed line: Median vitamin level. Black dashed line: Median outcome level. Purple diamond: Reference point.

**Figure S13. Associations between ln-transformed vitamin E intake and biological aging indicators by restricted cubic spline**

**A.KDM-acceleration**

**
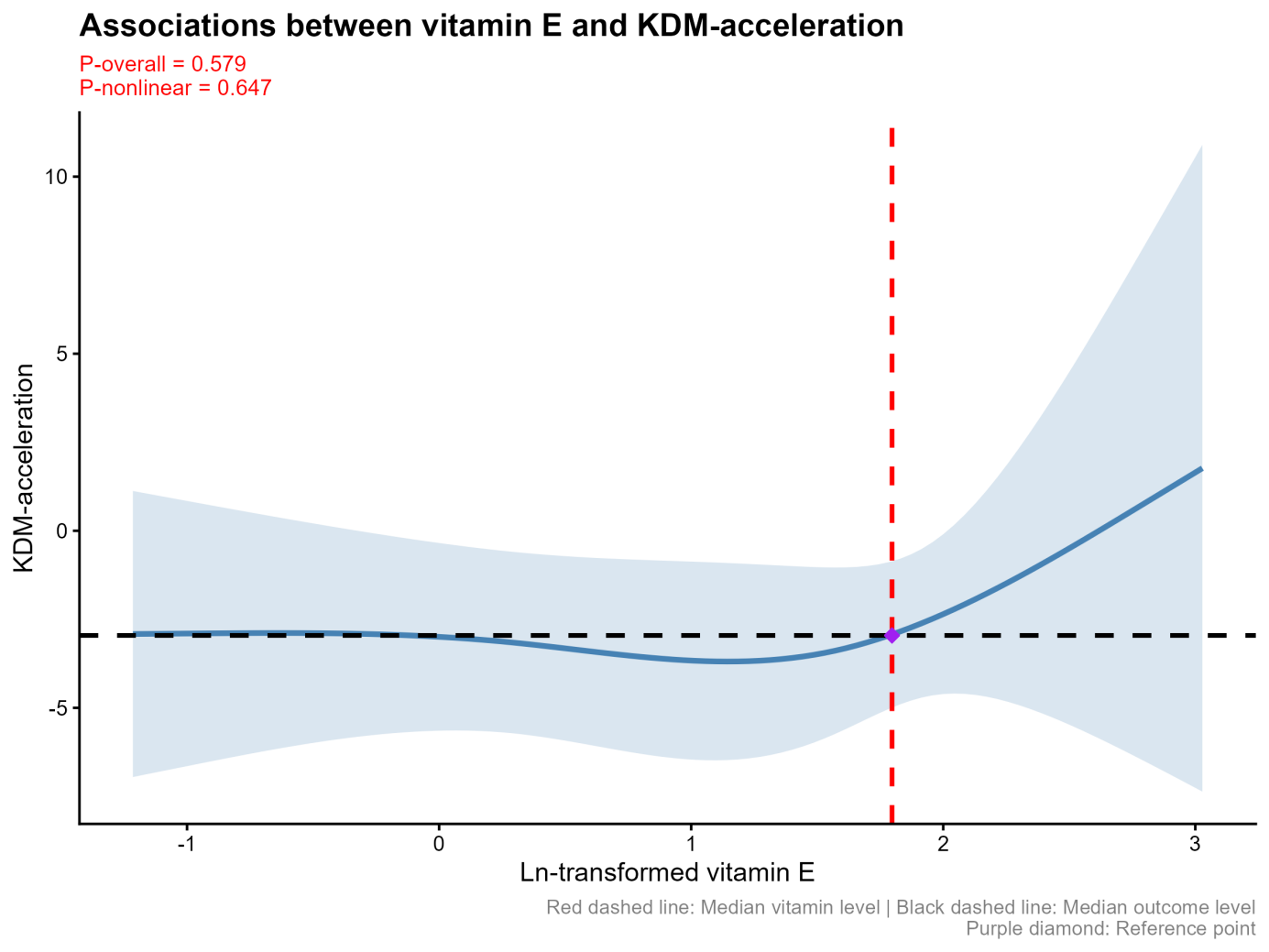
**

**B.PhenoAge-acceleration**

**
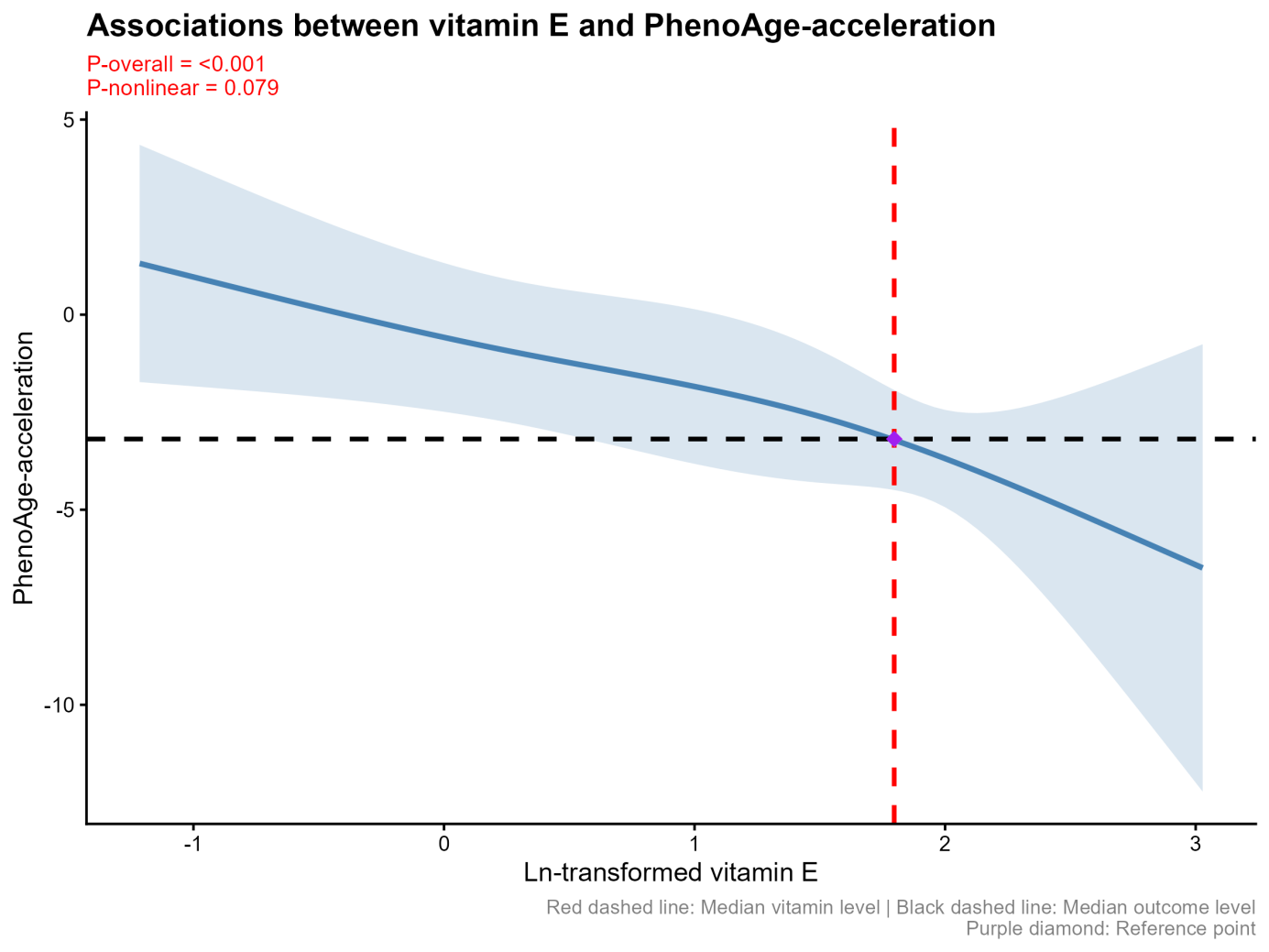
**

**C.HD**

**
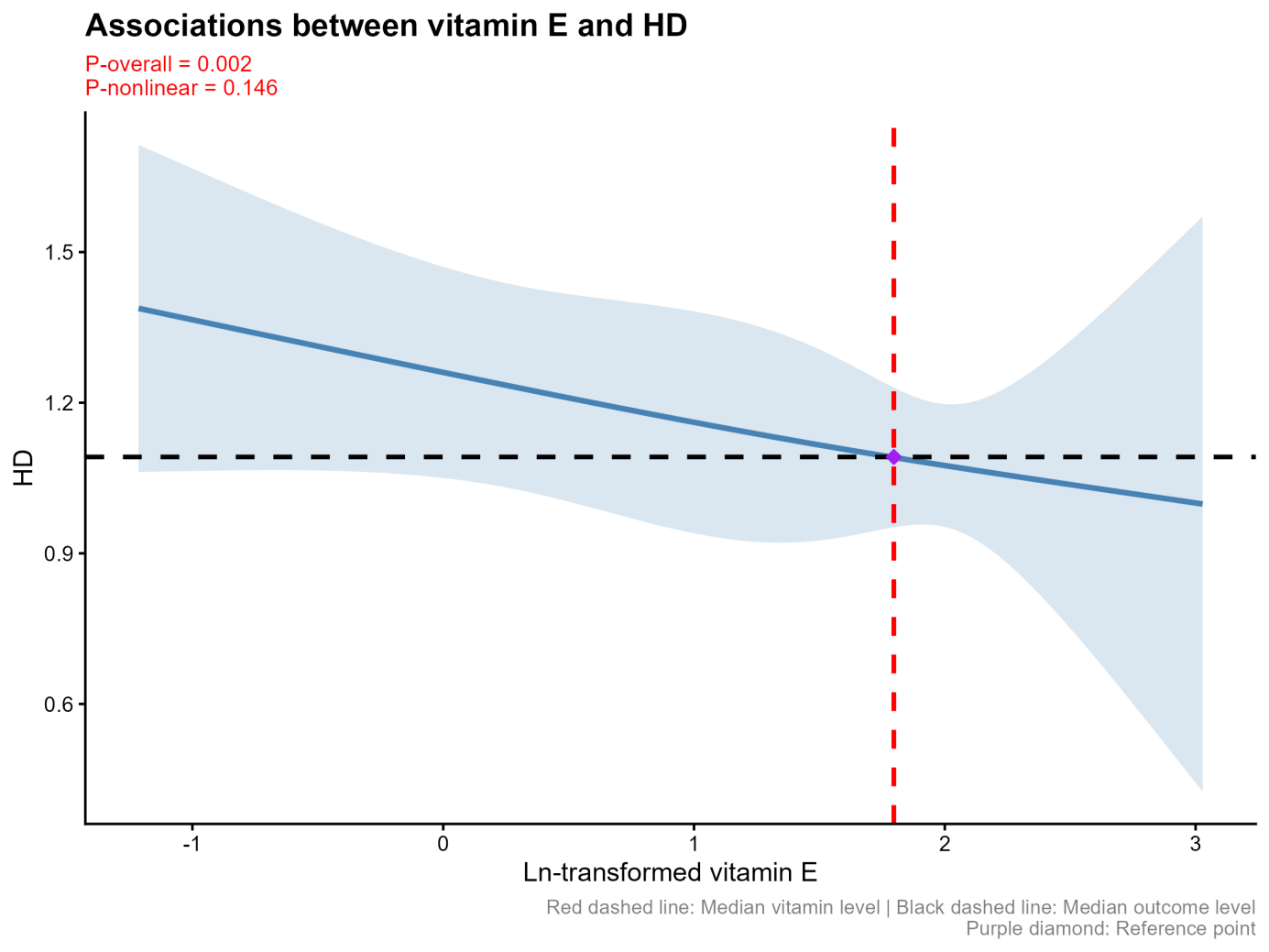
**

Abbreviations: KDM-acceleration, residual-based acceleration of Klemera and Doubal Model biological age (KDM-BA) relative to chronological age; PhenoAge-acceleration, residual-based acceleration of PhenoAge relative to chronological age; HD, homeostatic dysregulation.

Model adjusted for age, sex, race, educational level, marital status, poverty-income ratio, body mass index, smoking status, alcohol consumption, physical activity level, daily energy intake, supplement use, and comorbidity.

Red dashed line: Median vitamin level. Black dashed line: Median outcome level. Purple diamond: Reference point.

**Figure S14. Associations between ln-transformed vitamin K intake and biological aging indicators by restricted cubic spline**

**A.KDM-acceleration**

**
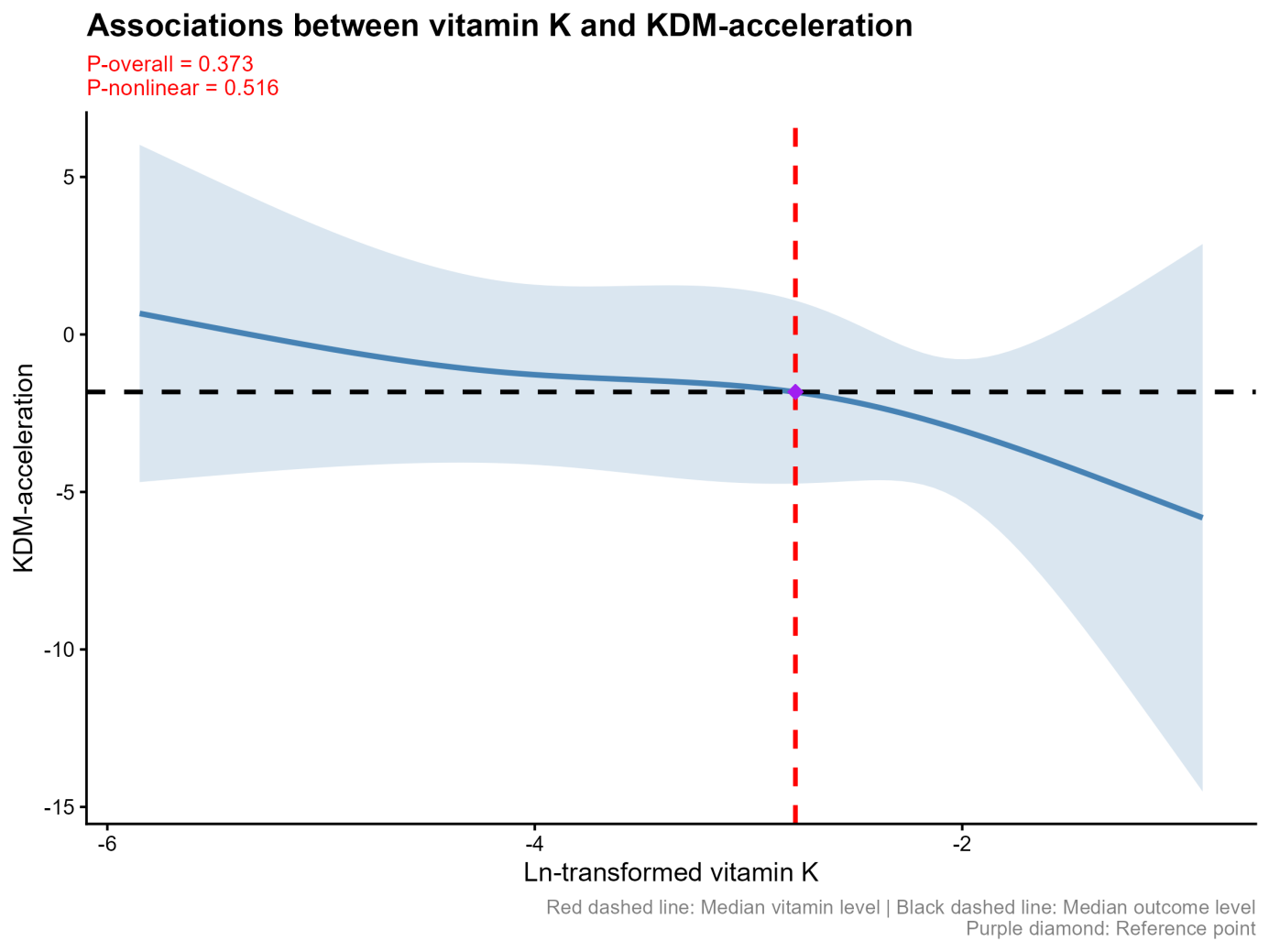
**

**B.PhenoAge-acceleration**

**
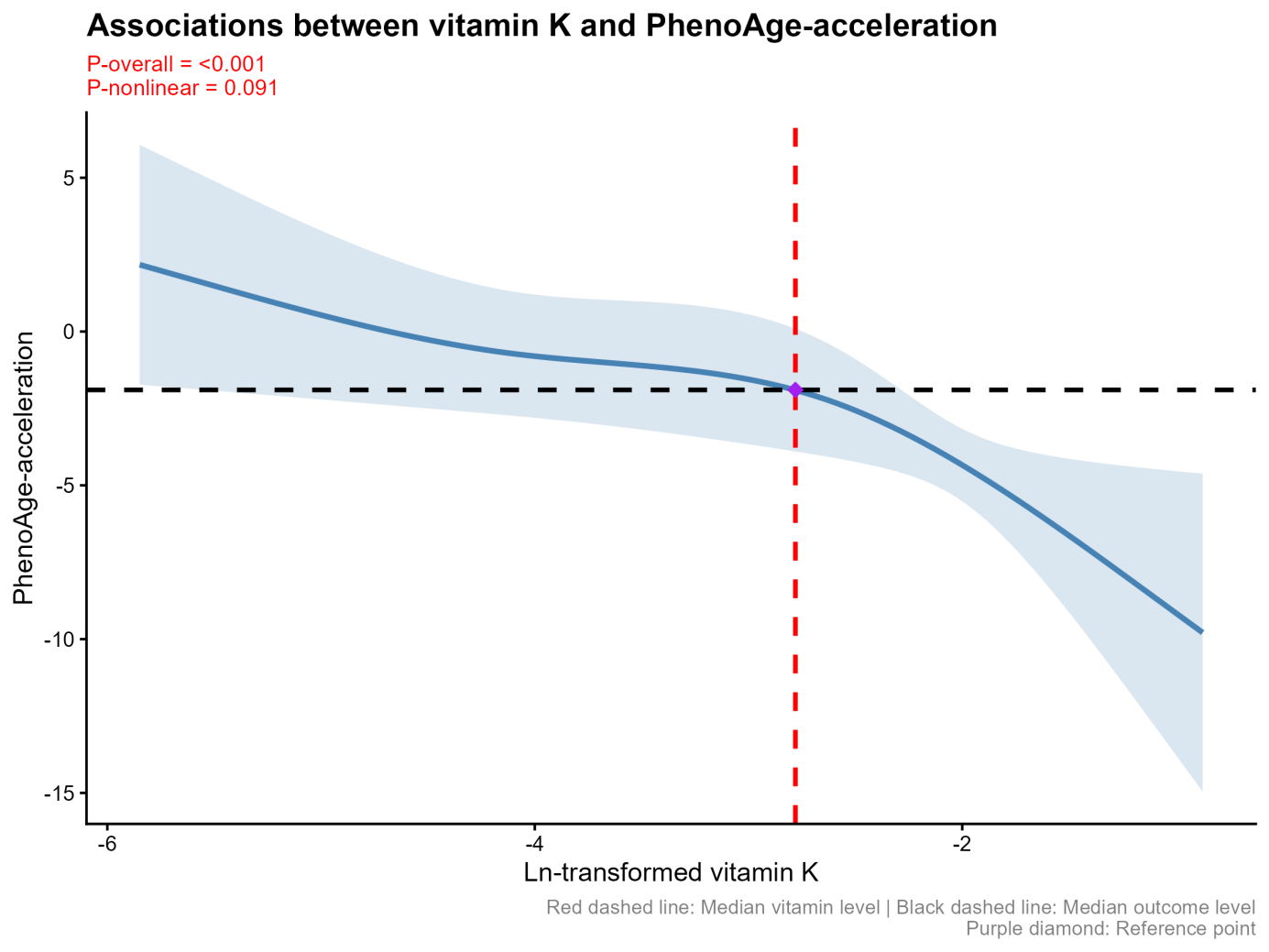
**

**C.HD**

**
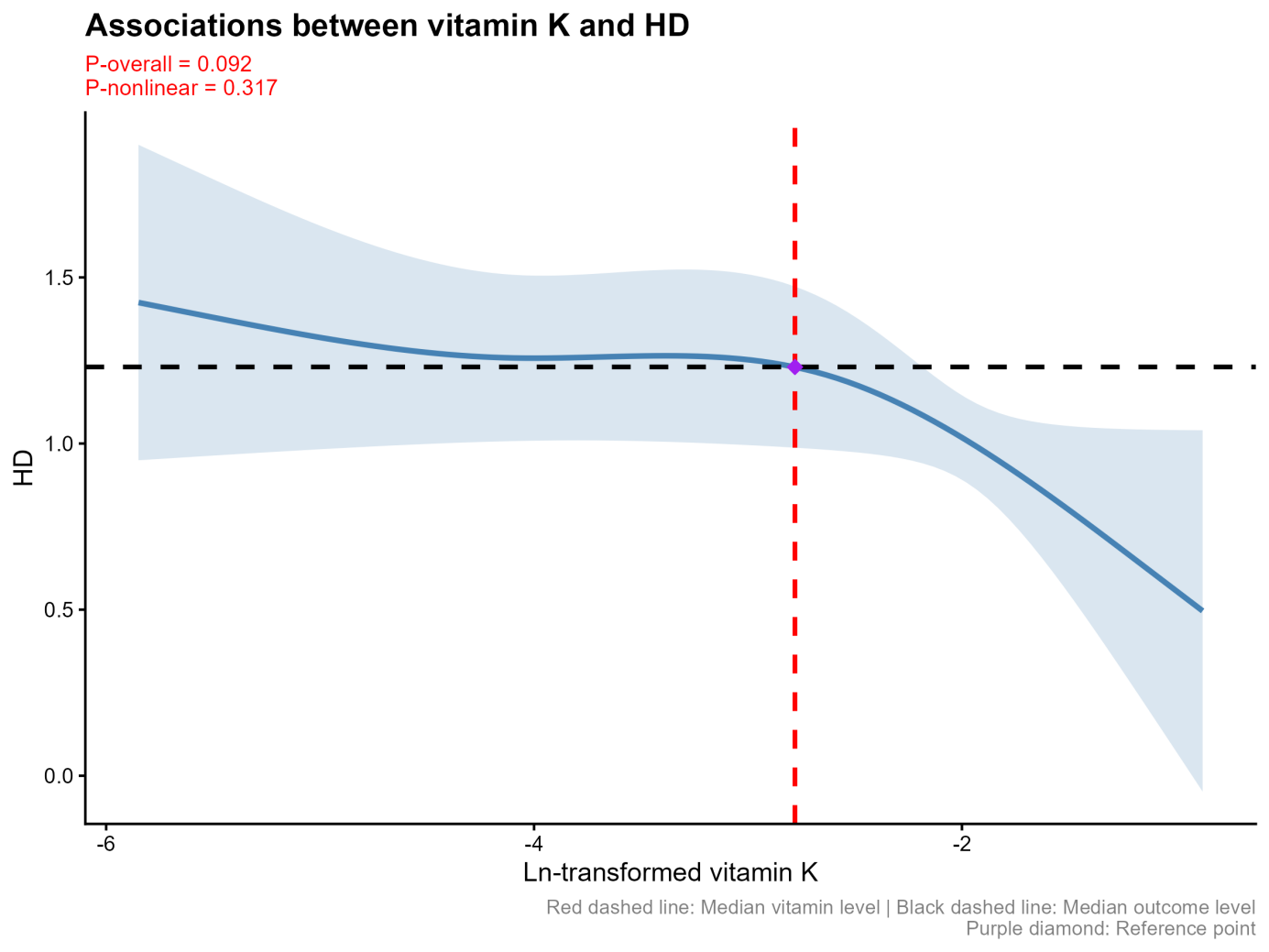
**

Abbreviations: KDM-acceleration, residual-based acceleration of Klemera and Doubal Model biological age (KDM-BA) relative to chronological age; PhenoAge-acceleration, residual-based acceleration of PhenoAge relative to chronological age; HD, homeostatic dysregulation.

Model adjusted for age, sex, race, educational level, marital status, poverty-income ratio, body mass index, smoking status, alcohol consumption, physical activity level, daily energy intake, supplement use, and comorbidity.

Red dashed line: Median vitamin level. Black dashed line: Median outcome level. Purple diamond: Reference point.

**Figure S15. Forest plot of the stratified analysis of associations between ln-transformed total vitamin and biological aging indicators**


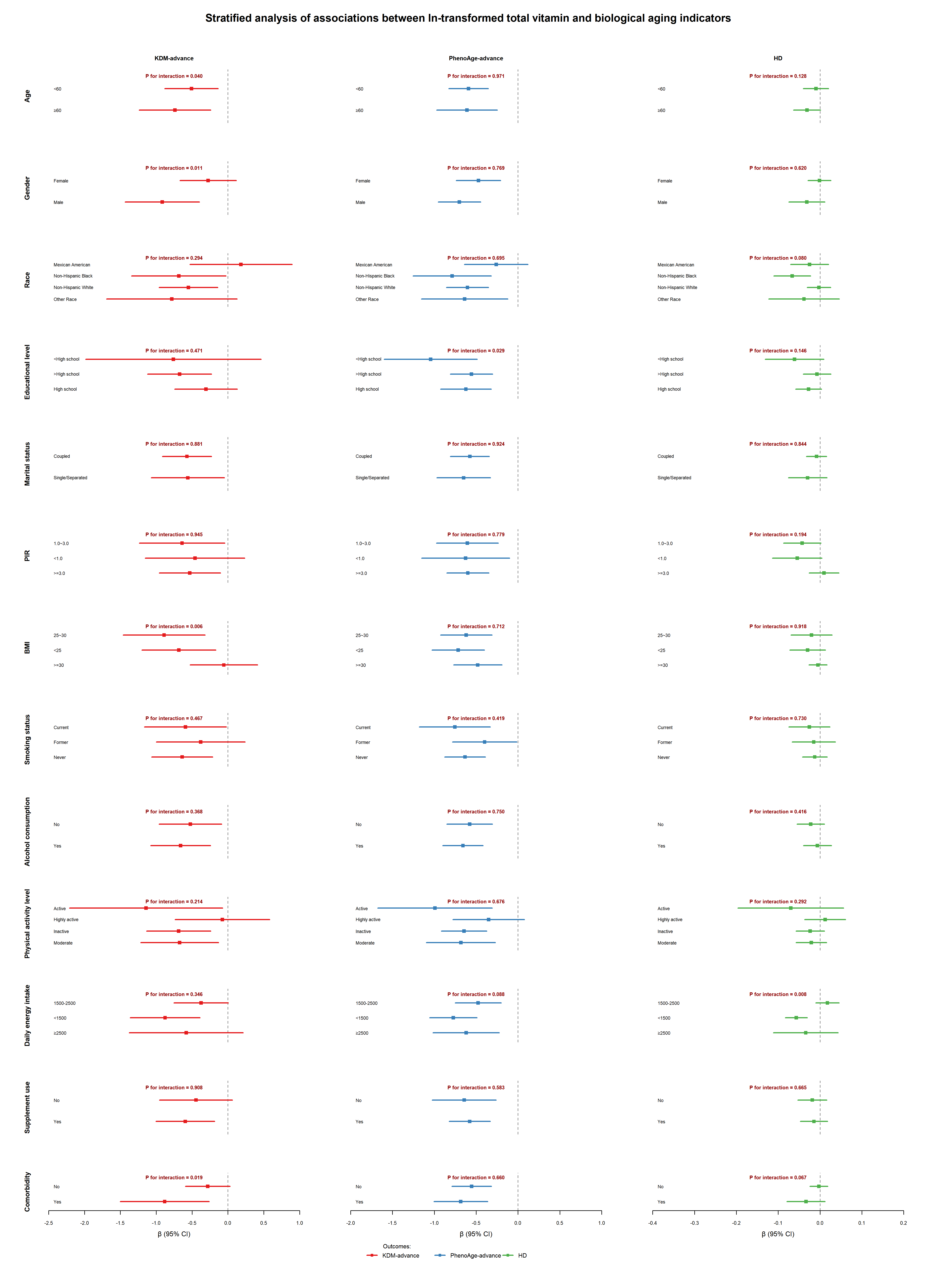


Abbreviations: BMI, body mass index; PIR, poverty-income ratio; KDM-acceleration, residual-based acceleration of Klemera and Doubal Model biological age (KDM-BA) relative to chronological age; PhenoAge-acceleration, residual-based acceleration of PhenoAge relative to chronological age; HD, homeostatic dysregulation.

Vitamin intake was used as continuous variables for analysis.

Model adjusted for age, sex, race, educational level, marital status, PIR, BMI, smoking status, alcohol consumption, physical activity level, daily energy intake, supplement use, and comorbidity.

**Figure S16. Associations between dietary-only vitamin intake and biological aging indicators by restricted cubic spline (sensitivity analysis)**

**A.KDM-acceleration**


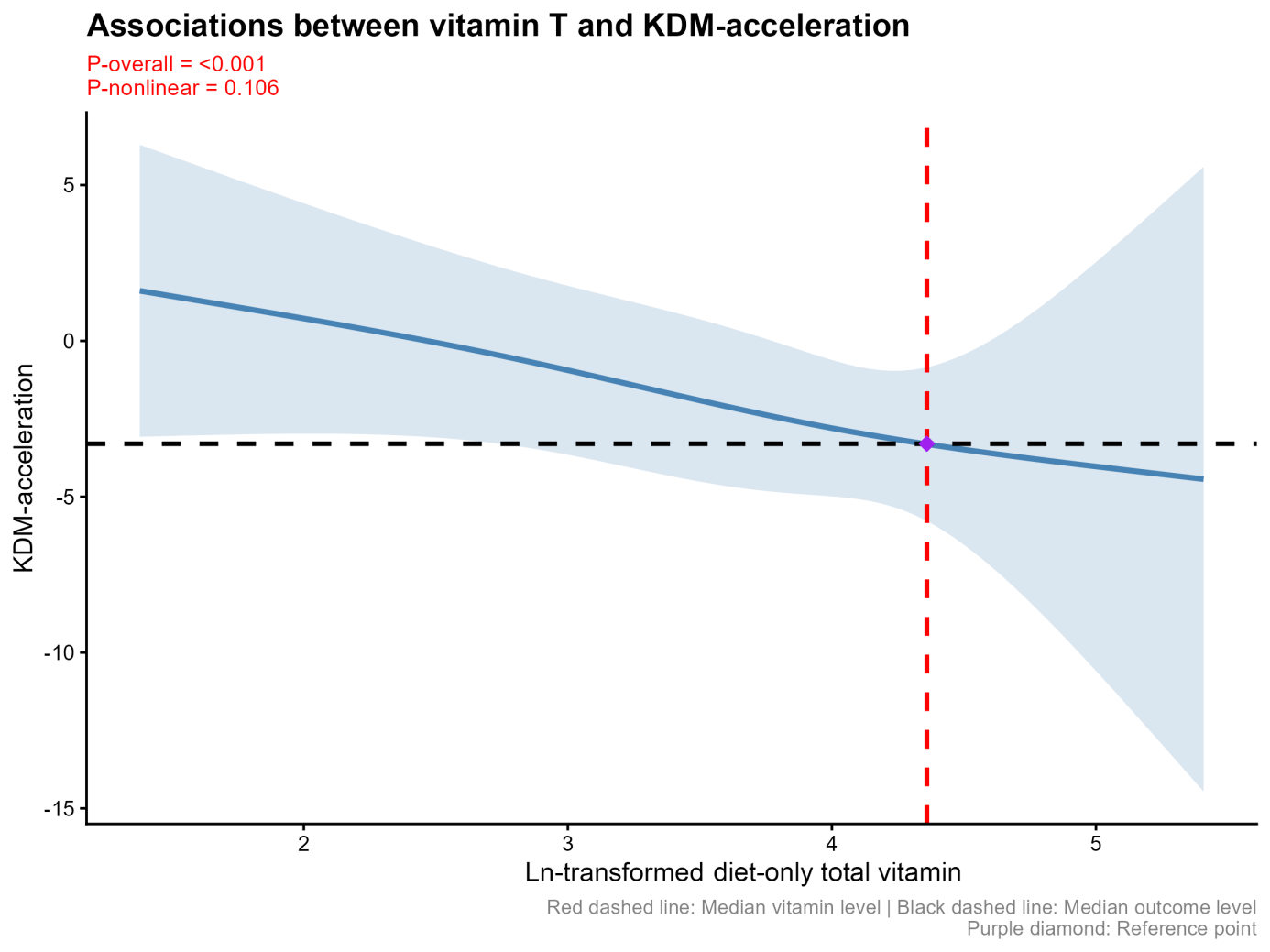


**B.PhenoAge-acceleration**


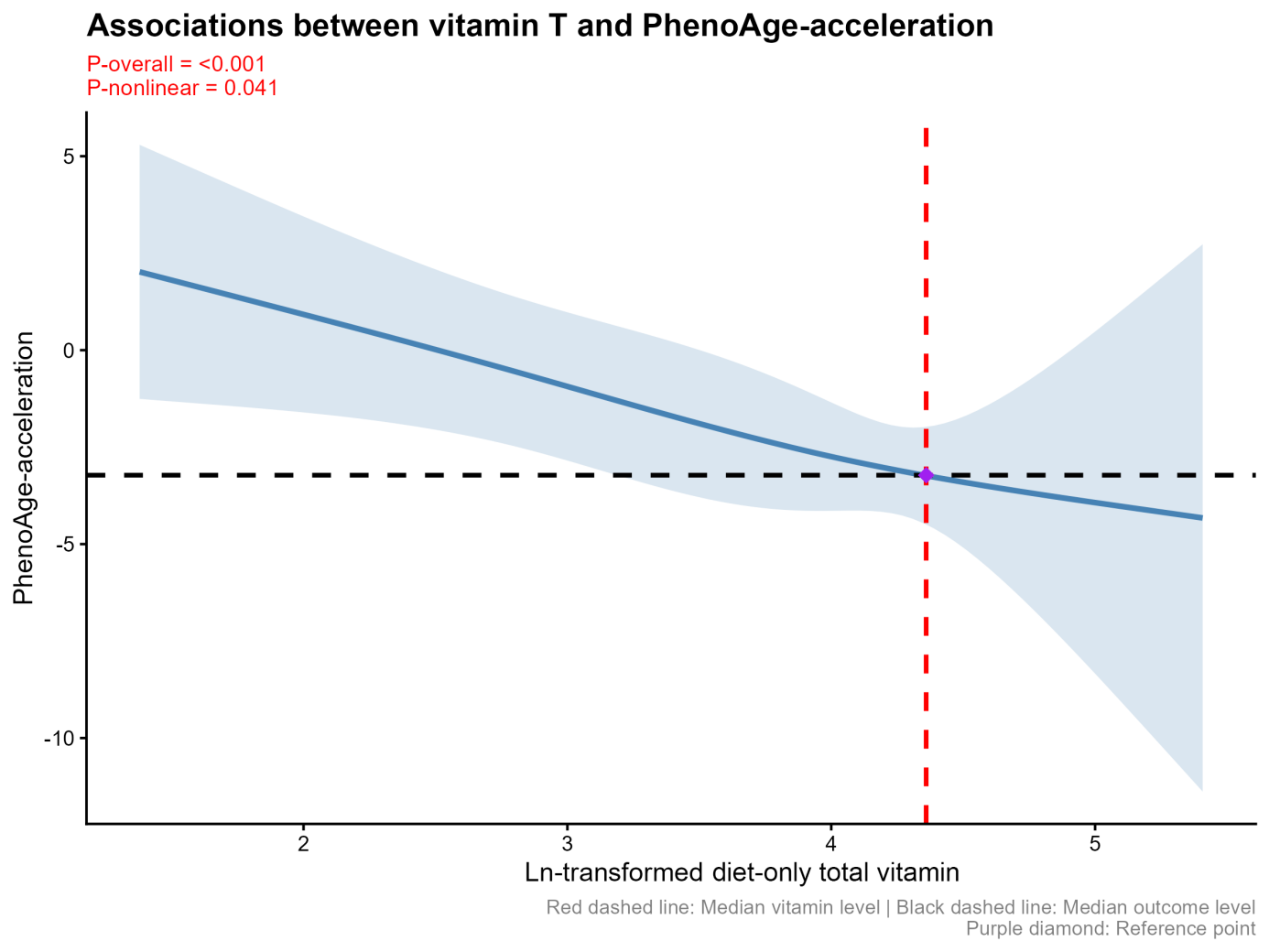


**C.HD**


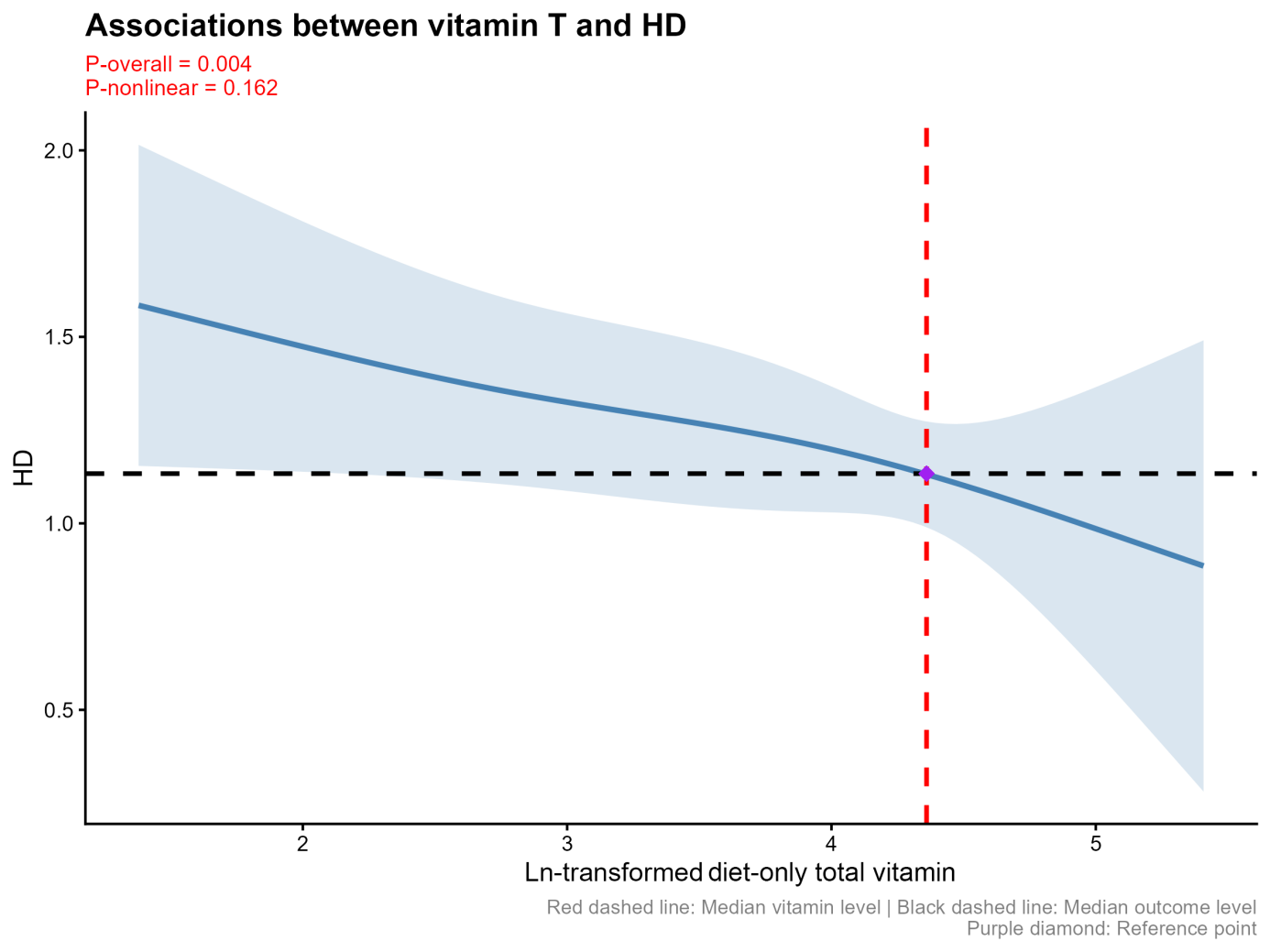


Abbreviations: KDM-acceleration, residual-based acceleration of Klemera and Doubal Model biological age (KDM-BA) relative to chronological age; PhenoAge-acceleration, residual-based acceleration of PhenoAge relative to chronological age; HD, homeostatic dysregulation.

Model adjusted for age, sex, race, educational level, marital status, poverty-income ratio, body mass index, smoking status, alcohol consumption, physical activity level, daily energy intake, supplement use, and comorbidity.

Red dashed line: Median vitamin level. Black dashed line: Median outcome level. Purple diamond: Reference point.

**Figure S17. Weights and individual effects of each dietary-only vitamin on biological aging indicators by quantile g-computation model (sensitivity analysis)**

**A.KDM-acceleration**


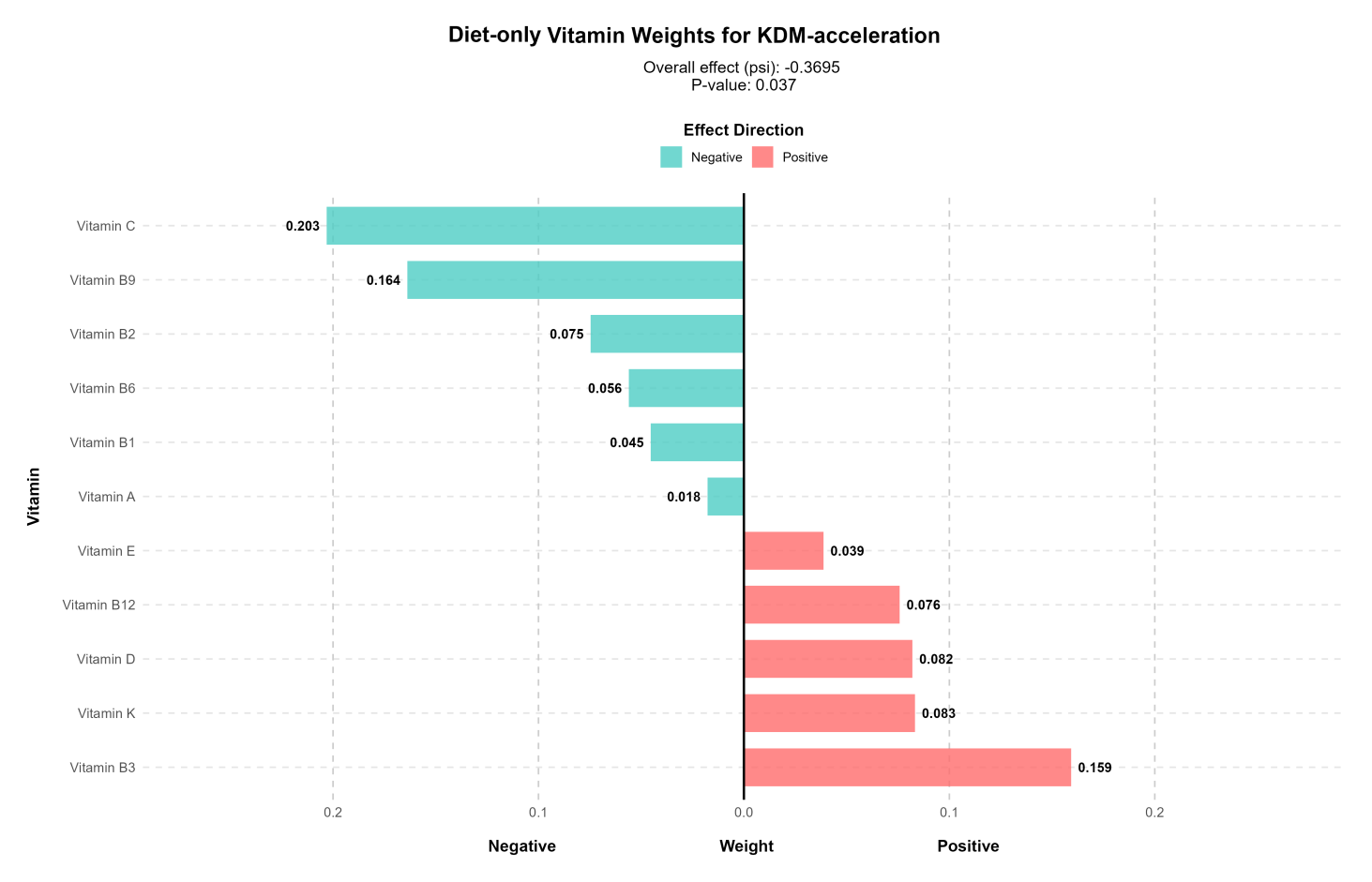


**B.PhenoAge-acceleration**


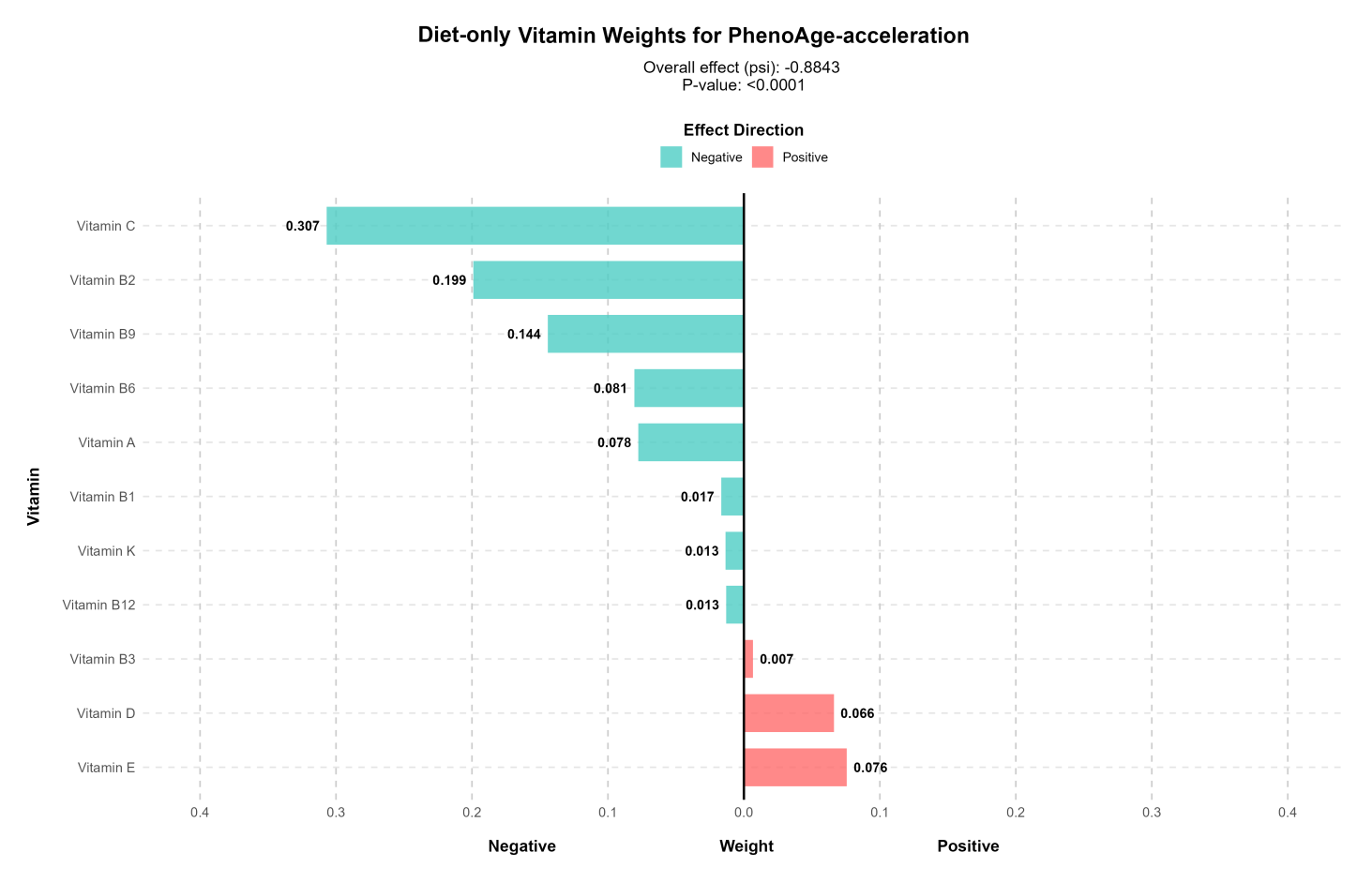


**C.HD**

**
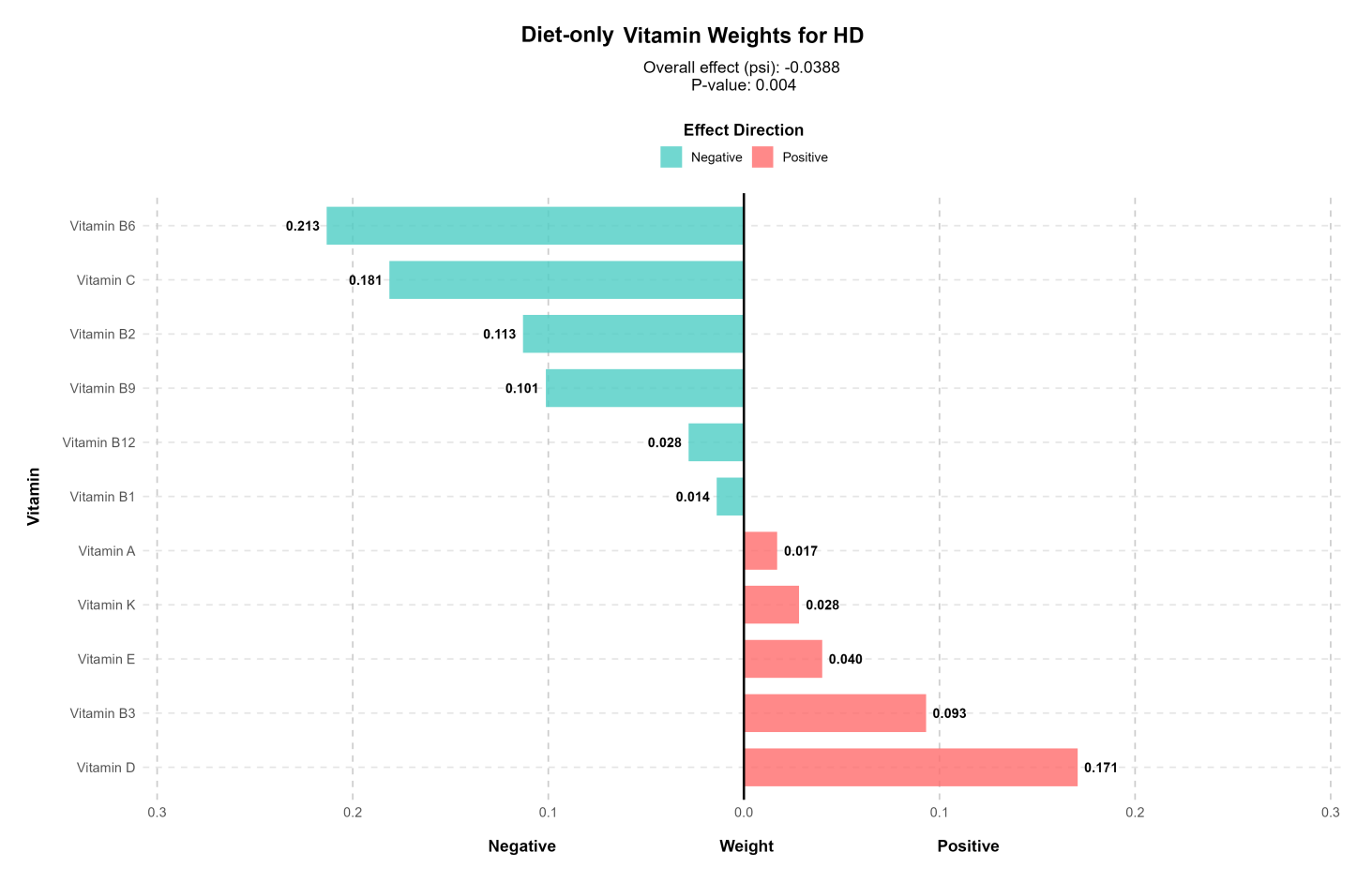
**

Abbreviations: KDM-acceleration, residual-based acceleration of Klemera and Doubal Model biological age (KDM-BA) relative to chronological age; PhenoAge-acceleration, residual-based acceleration of PhenoAge relative to chronological age; HD, homeostatic dysregulation.
